# Multifunctional Isosteric Pyridine Analogs-Based 2-Aminothiazole: Design, Synthesis, and Potential Phosphodiesterase-5 Inhibitory Activity

**DOI:** 10.3390/molecules26040902

**Published:** 2021-02-09

**Authors:** Abdel Haleem M. Hussein, Ahmed A. Khames, Abu-Bakr A. El-Adasy, Ahmed A. Atalla, Mohamed Abdel-Rady, Mohamed I. A. Hassan, Mahrous A. Abou-Salim, Yaseen A. M. M. Elshaier, Assem Barakat

**Affiliations:** 1 Department of Chemistry, Faculty of Science, Al-Azhar University, Assiut 71524, Egypt; abdelhaleemmh@yahoo.com (A.H.M.H.); a.khames@yahoo.com (A.A.K.); a_eladasy@azhar.edu.eg (A.-B.A.E.-A.); ahmedswify54@yahoo.com (A.A.A.); 2 Department of Chemistry, Faculty of Science, Assiut University, Assiut 71516, Egypt; mohamedrady2004@yahoo.com; 3 Department of Pharmacology & Toxicology, Faculty of Pharmacy, Al-Azhar University, Assiut 71524, Egypt; mohamed_aa74@yahoo.com; 4 Department of Pharmaceutical Organic Chemistry, Faculty of Pharmacy, Al-Azhar University, Assiut 71524, Egypt; mahrousabousalim@azhar.edu.eg; 5 Department of Organic and Medicinal Chemistry, Faculty of Pharmacy, University of Sadat City, Menoufiya 32958, Egypt; 6 Department of Chemistry, College of Science, King Saud University, P.O. Box 2455, Riyadh 11451, Saudi Arabia; 7 Department of Chemistry, Faculty of Science, Alexandria University, Alexandria 21321, Egypt

**Keywords:** 2-aminothiazoles, phosphodiesterase 5, sildenafil, erectile dysfunction, docking, OpenEye

## Abstract

The elaboration of new small molecules that target phosphodiesterase enzymes (PDEs), especially those of type 5 (PDE5), is an interesting and emerging topic nowadays. A new series of heterocycle-based aminothiazoles were designed and synthesized from the key intermediate, 3-oxo-*N*-(thiazol-2-yl)butanamide (a PDE5 inhibitor that retains its amidic function), as an essential pharmacophoric moiety. The PDE5 inhibitors prevent the degradation of cyclic guanosine monophosphate, thereby causing severe hypotension as a marked side effect. Hence, an in vivo testing of the target compounds was conducted to verify its relation with arterial blood pressure. Utilizing sildenafil as the reference drug, Compounds **5, 10a**, and **11b** achieved 100% inhibitions of PDE5 without significantly lowering the mean arterial blood pressures (115.95 ± 2.91, 110.3 ± 2.84, and 78.3 ± 2.57, respectively). The molecular docking study revealed that the tested compounds exhibited docking poses that were similar to that of sildenafil (exploiting the amide functionality that interacted with GLN:817:A). The molecular shape and electrostatic similarity revealed a comparable physically achievable electrostatic potential with the reference drug, sildenafil. Therefore, these concomitant results revealed that the tested compounds exerted sildenafil-like inhibitory effects (although without its known drawbacks) on blood circulation, thus suggesting that the tested compounds might represent a cornerstone of beneficial drug candidates for the safe treatment for erectile dysfunction.

## 1. Introduction

**Figure 1:**
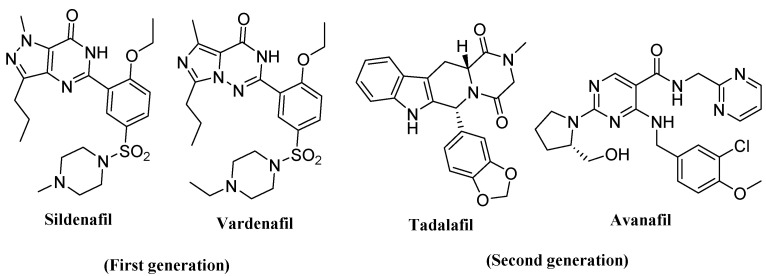
PDE5 inhibitors.

Erectile dysfunction (ED) is the persistent inability to achieve or maintain a penile erection that is sufficient for sexual performance [1]. The first-line treatment of ED patients involves the oral administration of phosphodiesterase type 5 inhibitors (PDE5), such as sildenafil [2], vardenafil [3], tadalafil [3], and the recent avanafil [4] (Figure 1), which hydrolyze cyclic guanosine monophosphate (cGMP) to GMP [5,6]. However, most of the patients experienced many side effects, including headache, blurred vision, facial flushing, and back pain [2,3,4], as well as severe hypotension. Sildenafil produces hypotension effect via its capability in inhibition of phosphodiesterase enzymes leading to the accumulation of cGMP that produces hypotension effect [3,4]. Among the drugs, sildenafil, which is a selective PDE5 inhibitor, possesses the following characteristic pharmacophoric features (Figure 1) (a) The lactam functionality of the pyrimidinone moiety, which forms hydrogen bond with the amino acid, GLN:817:A; (b) the pyrzolo[4,3-d]pyrimidine core, which exhibits a hydrophobic interaction with the amino acids, Val-782, Tyr-612, and Phe-820, in the binding pocket; and (c) the ethoxyphenyl group that fits into the hydrophobic pocket that was formed by the amino acids, Phe-786, Ala-783, Leu-804, and Val-782 [2,7]. Moreover, a second-generation selective PDE5 inhibitor, avanafil, possesses two hydrogen bonds with GLN:817:A and GLN:775:A, as well as a halogen bond with a backbone carbonyl oxygen (ALA:779:A) of an adjacent α-helix. These binding properties contribute to the inhibitory potency and illustrate the feasibility of exploiting the α-helix backbone in the structure-based drug design with the target PDE5-inhibitory molecules.

Conversely, the 2-aminothiazole-based heterocycles have attracted increased interest in the fields of drug design and discovery because of their effectiveness as small molecular drug candidates [8,9]. They are considered as a cornerstone for synthesizing many compounds with diverse pharmacological applications, such as anticancer [8,9,10,11,12], antibacterial [13,14], anti-inflammatory [15,16,17], antileishmanial [18], and influenza neuraminidase inhibitors [19], as well as PDE5 regulators and cyclooxygenases 1 and 2 (COX-1/COX-2) inhibitors [20].

**Figure 2:**
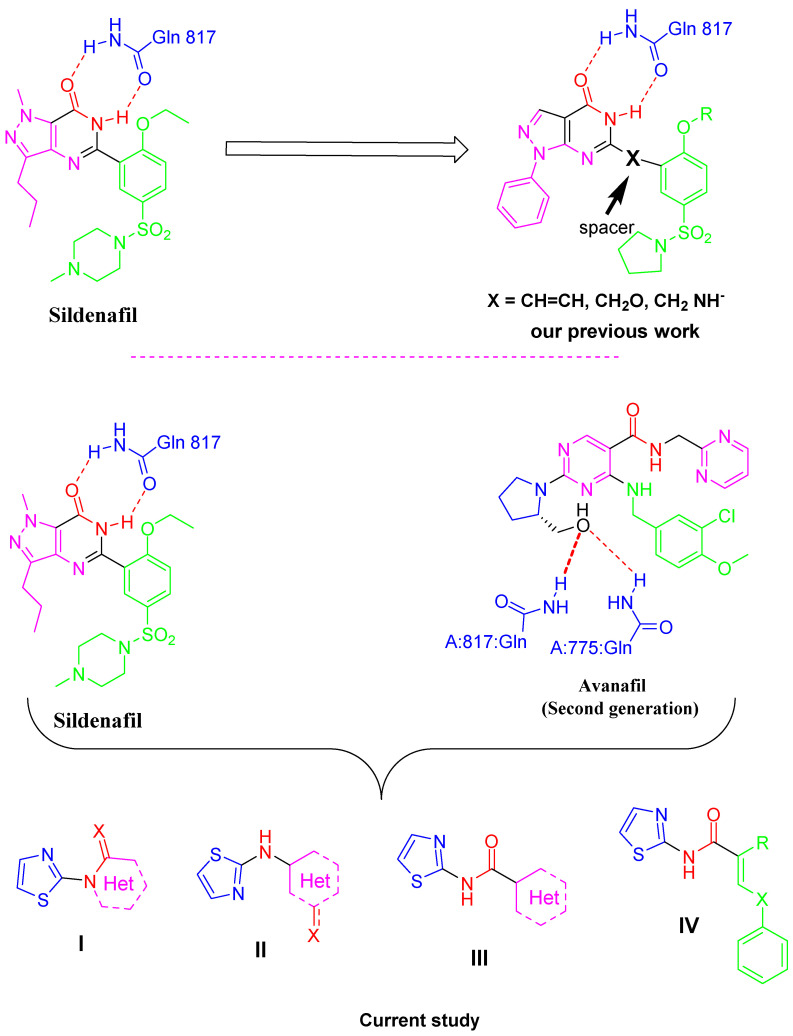
Medicinal chemistry design of current study.

Therefore, the development of new PDE5 inhibitors, which guarantees the adequate safety of the circulatory-system, is still a challenge for many medicinal chemists. The design of these compounds was elaborated by keeping the most important pharmacophores especially amide functionality aiming to get more selective PDE5 inhibitors. Based on our previous studies [7], we envisioned the design and synthesis of new derivatives of PDE5 inhibitors possessing the characteristic features of the first and second generations to treat ED without affecting the blood pressure. Therefore, the design strategy was developed to synthesize different heterocyclic ring-based 2-aminothiazole motifs by exploiting the essential pharmacophoric features of PDE5I [21,22], such as (1) a heterocyclic scaffold, namely the thiazole ring; (2) the amidic part to form essential HBs with GLN:817:A; and (3) the aryl part containing the polar substituents (Figure 2).

## 2. Results

### 2.1. Chemistry

**Scheme 1:**
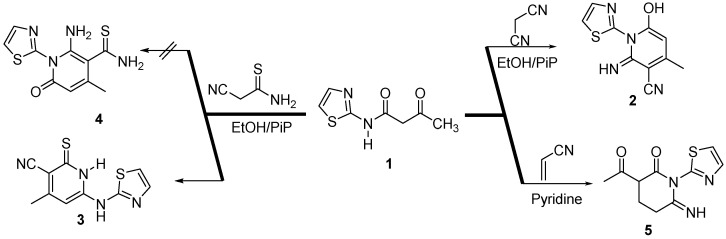
Synthetic routes for the **2**, **3**, and **5**.

The reactions of **1** [23] with different active methylene reagents were investigated. Thus, **1** reacted with malononitrile in ethanolic piperidine to yield the pyridine derivative, **2**, as the only isolated product. Additionally, **1** reacted with cyanothioacetamide under the same experimental conditions to yield the pyridinethione derivative, **3**, in a quantitative yield rather than pyridine (**4**). The structure of **3** was elucidated by different spectroscopic techniques, as follows: the IR data revealed a nitrile functionality at ν = 2247 cm^−1^, ^13^C NMR revealed a peak at δ 119.17 ppm, and ^1^H NMR revealed two NH groups at δ 8.70 and 12.58 ppm. Other elemental analyses were also conducted. Conversely, **1** reacted with acrylonitrile under reflux with pyridine to yield the tetrahydropyridone derivative, **5** (Scheme 1).

**Scheme 2:**
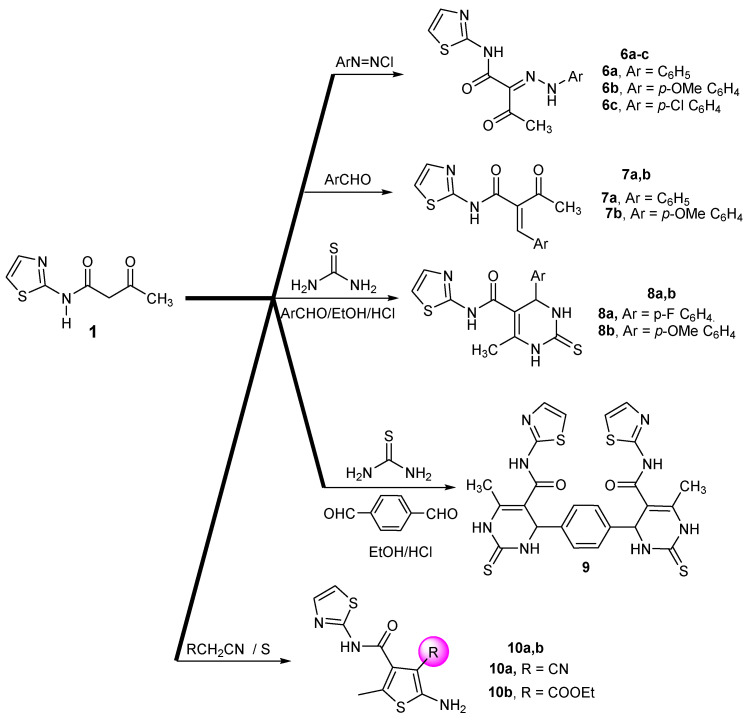
Synthetic routes for the compounds **6**–**10**.

Table 1 shows the aromatic diazonium salt as a route to new azines and condensed azines with potential activities. Therefore, **1** coupled smoothly with the aromatic diazonium salt to yield the corresponding arylhydrazones, **6a**–**c** in good yield [24]. However, the condensation of **1** with the aromatic aldehydes in a basic medium afforded the arylidine derivatives, **7a**,**b** [25]. Moreover, **1** reacted with a mixture of aromatic aldehydes, such as *p*-florobenzaldehyde, *p*-anisaldehyde, and thiourea to yield the expected pyrimidine thiones, **8a**,**b** [26]. Similarly, **1** reacted with a mixture of terphthaldehyde and thiourea to afford the bis-structure, **9**, which was confirmed according to its compatible spectroscopic data. The condensation reaction of **1** with malononitrile/ethylcyanoacetate and elemental sulfur under reflux in ethanol/morpholine afforded the thiophene derivatives, **10a**,**b** (Scheme 2).

**Scheme 3:**
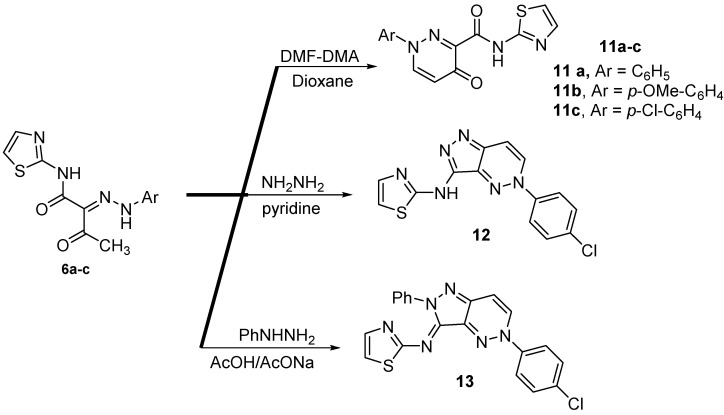
Synthetic routes for the compounds **12** and **13**.

Moreover, compounds **6a**–**c** were condensed with DMF–DMA to yield the pyridazinones, **11a**–**c**, in excellent yield. The reflux of **11c** with hydrazine hydrate in pyridine afforded **12**, while its treatment with phenylhydrazine under solvent-free conditions yielded **13** (Scheme 3).

### 2.2. In Vitro PDE5 Inhibitory Activity Assay

As presented in Table 1, the synthesized compounds were tested for their in vitro PDE5 inhibitory activity employing sildenafil as a reference drug at a concentration of 10^4^ nM [27]. As indicated by the results, the tested compounds were classified into three main groups based on their inhibitory activities: **2**, **5**, **10a**,**b**, and **11b** (82%) completely inhibited PDE5 (100%) compared to sildenafil, while **7a,b** exhibited moderate activities (38% and 62%, respectively). Furthermore, **3**, **6b**,**c**, **8b**, **11c**, **12**, and **13** were tested, and they demonstrated weak inhibitory activities against PDE5.

### 2.3. Pharmacology

The PDE5 inhibitors potentiated the effect of NO by preventing the degradation of cGMP, thereby enhancing vasodilation and increasing blood flow, although with severe hypotension as a marked side effect. Hence, the target compounds were not subjected to in vivo testing to verify the existence of any effect on MABP, and the data of the vasorelaxant effects of the tested compounds are presented in Table 1. The data revealed that the MABP values of all the compounds except **2** are higher than those of nitroprusside and sildenafil. Compound **2** values concerning its effect on MABP are less than that of sildenafil, and more than that of nitroprusside (Table 1).

Compounds **1**, **6**, **11b**, **10a**,**b**, **5**, and **6c** significantly increased MABP in the control rabbits (Table 1). Compounds **9**, **8b**, **12**, **7a**,**b** did not exert any significant difference from those of the control, but their MABP values were still significantly higher than that of sildenafil (Table 1). Compounds **9**, **8b**, **12**, **7a**,**b** did not reveal any significant difference from the control regarding their effects on MABP, but there was a significant difference from the effect of sildenafil, where their values are markedly higher (Table 1). Compounds **11b**, **13**, **2**, **12**, and **3** significantly lowered MABP compared with the control, but their values were still higher than that of sildenafil, although non-significantly (Table 1). Compounds **2** and **11b** exhibited great correlation in their inhibitory actions on PDE5 and their effects on MABP.

Surprisingly, **5**, **10a**,**b**, and **11b** completely inhibited PDE5 without severe hypotension, while **2** inhibited PDE5 (100%), although severe hypotension was observed. Regarding compounds **2**, **5**, **10a**, and **11b**, all of them inhibit phosphodiesterase 5 enzyme by 100%. Compound **2** illustrated lowering in the mean arterial blood pressure (MABP) to a level less than that produced by sildenafil. Compound **11b** reduced the MABP to a level higher than that produced by sildenafil. Finally, compounds **10a**, and **5** increased the MABP, both of them can be used in erectile dysfunction accompanied with hypotension. Accordingly, these compounds in addition to their effects on PDE5, have another action on blood pressure. The contrasting effect of compound **2** could be due to it rigidity and the ability of its amide bond and or pyridine moiety to form stronger HB. Generally, these results suggest the promising therapeutic roles of **5**, **10a**,**b**, and **11b** in treating ED without hypotension (the serious side effect that limits the utilization of PDE5 inhibitors). This merit might be related to the pharmacophoric structural features, especially that in which the thiazole moiety was endowed with either pyridazine or the thiophene heterocycles through an amide bridge, thus confirming their shape and electrostatic similarities with sildenafil.

Interestingly, the common properties of the most active compounds are due to the following features: (a) the amide that is attached to thiazole; (b) the heterocyclic rings that are tethered to thiazole, such as the pyrimidine ring (responsible for the unfavorable results), pyridazine or pyridine moieties (responsible for the good interactions); and (c) the unsubstituted aryl group. Tethering the aryl ring with an electron-donating group (EDG) is better than with an electron-withdrawing group. Hence, the tethering of the thiazole moiety with a multifunctional heterocyclic ring (piperidine, pyridazine, or thiophene) through an amide bridge along with an EDG aryl group could improve the inhibitory activity without the dangerous effect on the blood pressure.

### 2.4. Molecular Modeling

#### 2.4.1. Lead Optimization by Scaffold Hopping

Then molecular shape and electrostatic similarity were determined by the OpenEye EON scientific software by replacing all the molecules with the reference drug (sildenafil) to ensure physically realistic electrostatic potential comparisons [28]. The results revealed that the designed compounds possessed similar shapes and electrostatic potentials to sildenafil as shown in Figures S1–S3 (supplemental information). This conclusion was resolved based on the high EON-ET-Combo score, which is a beneficial score that considers the shape and electrostatic matches (the closest in the biological activity, as presented in Table 2).

#### 2.4.2. Structure-Based Lead Discovery

The designed synthesized compounds were docked into PDE5 with PDB ID, 2H42 [29]. To validate our study, the docking protocol commenced with sildenafil (the standard ligand that was co-crystallized with 2H42). The designed compounds were first subjected to a filtering process employing the OpenEye filter application [30], which functions by calculating the properties, such as MW, XlogP, XlogS, PSA, hydrogen-bond donor and acceptor counts, rotatable bonds, ring size, and number, etc. Moreover, the filtering process assigns a graph-based protonation state for consistency and speed and offers ADME filters, such as Lipinski, Egan, Veber, and Martin. The compounds with impossible bonding and inappropriate elements were removed. Thereafter, the library was processed by the OpenEye Omega application in which the 3D conformations of the molecules were generated. Next, the OpenEye docking was performed with the FRED application [31,32].

**Figure 3:**
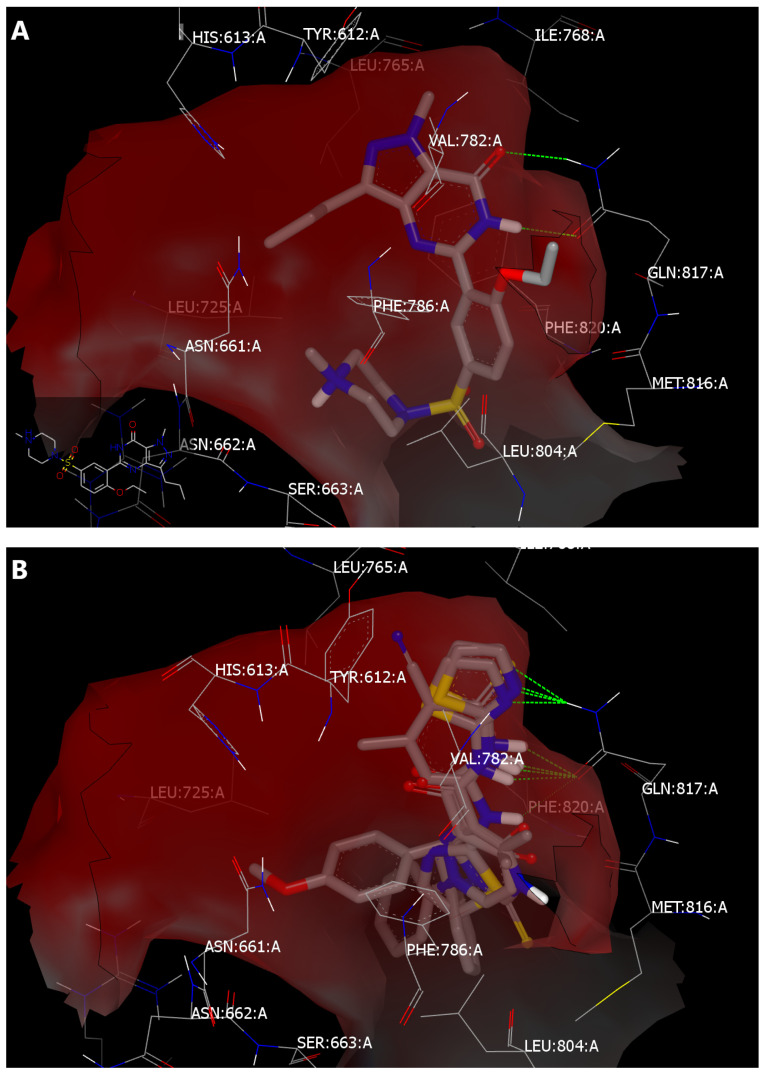

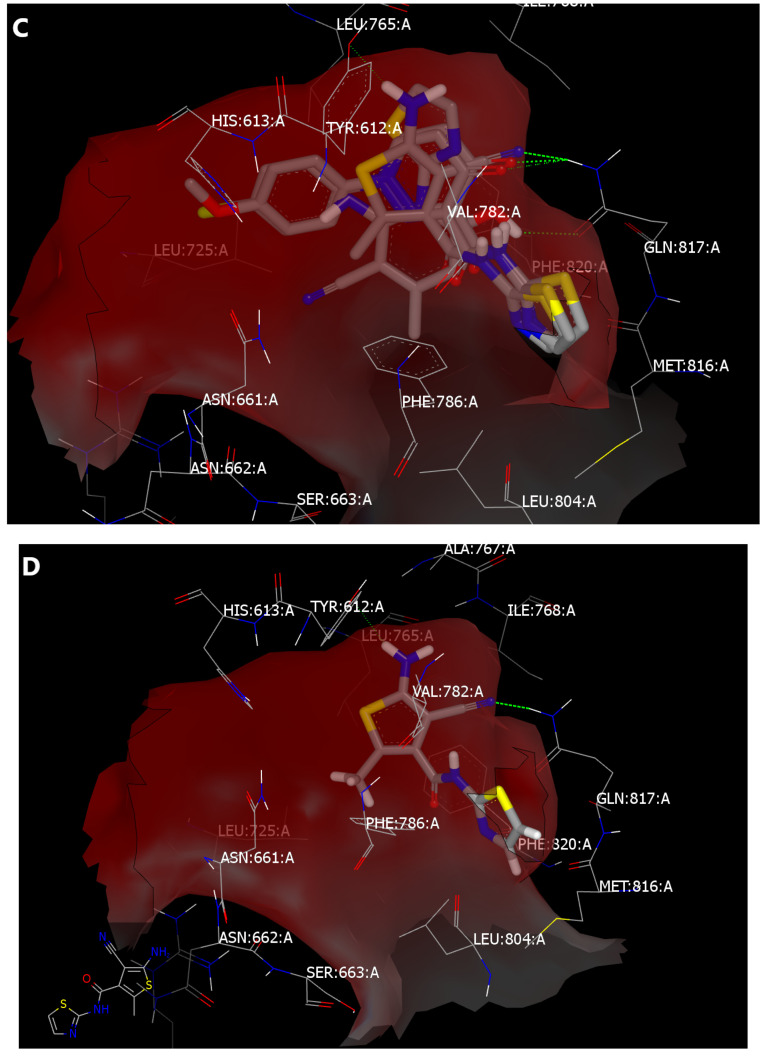

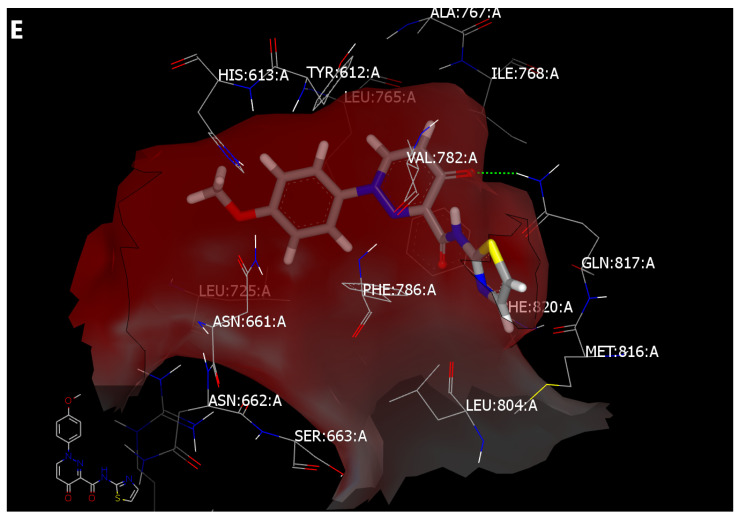
(**A**) Sildenafil docked with PDB ID: 2H42 with formation of two HBs and hydrophobic–hydrophobic interaction; (**B**) overlay of compounds **3**, **7a**, **8b**, and **11a** in the same pane of sildenafil with formation of two HBs with GLN:817:A; (**C**) overlay of compounds **2**, **10a**, **11b**,**c** with the formation of one HB with GLN:817:A; (**D**) compound **10a** forms two HBs with GLN:817:A and TYR:612:A in addition to hydrophobic–hydrophobic interaction; (**E**) compound **11b** forms one HB with GLN:817:A in addition to hydrophobic–hydrophobic interaction.

The FRED-3.5.0.4 2020-released OpenEye docking application employs chemgauss4 as the state-of-the-art scoring function, which is the total score of both the favorable (shape, hydrogen bonding, and metal chelation) and unfavorable (desolvation and clash) interactions. The comparative FRED chemgauss4 scores of the top-scoring synthesized compounds are depicted in Table 3 (the lower the score, the better the binding affinity) and are shown in Figures S4–S15 (supplemental information). The standard drug, sildenafil, featured hydrophobic–hydrophobic interactions, and it possesses two hydrogen bonds with GLN:817:A through the pyrimidine NH (bond length = 1.92 Å) and carbonyl (bond length = 2.16Å) groups (Figure 3A). This mode of interaction is similar to its reported docking [29]. Generally, **3**, **7a**, **8b**, and **11a** overlaid in that same pane of sildenafil with the formation of two hydrogen bonds with GLN:817:A, while **2**, **10a**, **11b**, and **11c** each formed a hydrogen bond with GLN:817:A (Figure 3B,C, respectively). Compound **7b** possessed a hydrogen bond with GLN:817:A, while **10b** and **8a** did not, although they all displayed in their various pane. Interestingly, the tested biologically active compounds, especially those that achieved 100% inhibition of PDE5, interacted with the receptor in a similar mode as did sildenafil (they formed HB of higher energy with GLN:817:A). Regarding **10a**, it interacted with the receptor through the formations of two HBs: one was formed from the interaction of the nitrile group with GLN:817:A (bond length = 1.59 Å), while the second one with lower energy was formed between the amino group and TYR:612:A (bond length = 2.46 Å) (Figure 3D). Regarding **11b**, it formed HB between the pyridazine carbonyl group and the amino group of GLN:817:A (bond length = 1.84 Å) (Figure 3E). The detailed docking results are reported in the Supplementary Materials.

Although **5** was not processed for docking because it was filtered out in the Omega process because of some differences in its physicochemical properties, it was subjected to laboratory testing, where it revealed 100% inhibition of PDE5.

## 3. Materials and Methods

### 3.1. General

All the melting points were uncorrected. The infrared (IR) spectra (KBr) were recorded on a Fourier-transform IR (FTIR) 5300 spectrometer (Nicolet™ iS™ 10, Madison, WI, USA) (ν, cm^−1^). The one-dimensional (1D) nuclear magnetic resonance (NMR) (1D NMR): ^1^H NMR, ^13^C NMR, the distortionless enhancement by polarization transfer (DEPT) 135, and the nuclear Overhauser effect (NOE) ^13^C NMR and the two-dimensional (2D) (2D NMR): the homonuclear correlation spectroscopy (HH COSY) and heteronuclear COSY (CH COSY) spectra were recorded in DMSO-*d*_6_ and CDCl_3_ at 400 and 500 MHz on a JEOL (JEOL, Tokyo, Japan) and a Brucker (Avance lll, Zürich, Swetzerland) NMR spectrometer (δ, ppm) employing tetramethylsilane (TMS) as an internal standard, respectively. The mass spectra were obtained on a JEOL JMS600 H Root mass spectrometer (JEOL, Tokyo, Japan) at 70 eV. The elemental analysis was conducted by the Microanalytical Research Center, Faculty of Science, Cairo University, and the Microanalytical Research Center, Assiut University Broker Company, Switzerland Center. The spectral data are shown in Figures S16–S51 (supplemental information).

### 3.2. Syntheses

#### 3.2.1. 6-Hydroxy-2-imino-4-methyl-1-(thiazol-2-yl)-1,2-dihydro-pyridine-3-carbonitrile (**2**)

A mixture of 3-oxobutanamide (**1**) (1.84 g, 10 mmol) and an appropriate active methylene reagent (malononitrile) (0.66 g, 10 mmol) in ethanol (30 mL) was treated with a few drops of piperidine and refluxed for 12 h, after which it was left to cool. The resultant solid product was filtered out and recrystallized from ethanol to obtain **2** (78%) as pale-yellow crystals. m.p. = 210 °C; IR (KBr): ν = 3312 (O–H), 3058 (ArC–H), 2921 (AliC–H), 2215 (C≡N), 1673 (C=O) cm^−1^; ^1^H NMR (CDCl_3_) δ: 1.64 (s, 1H, NH), 2.31 (s, 3H, CH_3_), 5.91 (s, 1H, CH–pyridine), 7.35 (d, 1H, CH–thiazole, *J* = 3.2 Hz), 7.70 (d, 1H, CH–thiazole, *J* = 4 Hz), 8.76 (br, 1H, OH) ppm; ^13^C NMR δ: 20.91, 104.60, 116.79, 119.26, 135.67, 152.22, 155.30, 157.06, 159.61 ppm; DEPT 135 NMR δ: (+)20.87 (q), 104.54 (d), 119.21 (d), 135.61 (d); MS (relative intensity) *m*/*z*: 233.15 (M+, 26%). Anal. Calcd. for C_10_H_8_N_4_OS (232.26): C, 51.71; H, 3.47; N, 24.12; S, 13.81%. Found: C, 51.69; H, 3.46; N, 24.14; S, 13.82%.

#### 3.2.2. 4-Methyl-6-(thiazol-2-ylamino)-2-thioxo-1,2-dihydropyridine-3-carbonitrile (**3**)

A mixture of **1** (1.84 g, 10 mmol) and cyanothioacetamide (1 g, 10 mmol) in ethanol (30 mL) was treated with a few drops of piperidine and refluxed for 6 h, after which it was left to cool before it was poured into crushed ice and acidified with HCl. The solid product was collected and recrystallized from ethanol to yield **3** (67%) as dark-red crystals. m.p. = 170 °C; IR (KBr): ν = 2247.63 (C≡N) cm^−1^; ^1^H NMR (CDCl_3_) δ: 2.55 (s, 3H, CH_3_), 5.96 (s, 1H, CH Pyridine); 7.55 (d, 1H, CH thiazole, *J* = 4 Hz); 7.70 (d, 1H, CH thiazole, *J* = 4 Hz); 8.70 (s, 1H, NH), 12.58 (s, 1H, NH) ppm; ^13^C NMR δ: 12.26, 75.05, 104.62, 116.54, 119.17, 135.69, 152.42, 155.29, 157.34, 159.67 ppm; DEPT 135 ^13^C NMR δ: (+)12.34, 104.52, 119.41, 135.80. Anal. Calcd. for C_10_H_8_N_4_S_2_ (248.33): C, 48.37; H, 3.25; N, 22.56; S, 25.82%. Found: C, 48.34; H, 3.27; N, 22.58; S, 25.86%.

#### 3.2.3. 3-Acetyl-6-imino-1-(thiazol-2-yl)piperidin-2-one (**5**)

A mixture of **1** (1.84 g, 10 mmol) and acrylonitrile (0.53 g, 10 mmol) in pyridine (30 mL) was heated under reflux for 6 h, after which the reaction mixture was allowed to cool before it was poured onto crushed ice and acidified with a few drops of HCl. The obtained solid product was filtered off and recrystallized from the aqueous ethanol to yield **5** (63%) as yellow crystals. m.p. = 170 °C; ^1^H NMR (CDCl_3_) δ: 2.12 (s, 3H, CH_3_), 2.17–2.39 (m, 2H, CH_2_–pyridine), 2.84 (t, 2H, CH_2_–pyridine, *J* = 6.4 Hz), 4.35 (t, 1H, CH–pyridine, *J* = 6.4 Hz), 6.80 (d, 1H, CH–thiazole, *J* = 4 Hz), 7.17 (d, 1H, CH–thiazole, *J* = 5.2 Hz), 9.69 (s, 1H, NH) ppm; ^13^C NMR δ: 12.68, 26.95, 27.44, 63.44, 110.48, 119.21, 126.25, 167.66, 177.58, 205.05, ppm; DEPT 135 ^13^C NMR δ: (+)12.76, (−)27.03, (−)27.51, (+)63.44, 110.57, 126.35; MS (relative intensity) *m*/*z*: 238 (M + 1, 3.7%). Anal. Calcd. for C_10_H_11_N_3_O_2_S (237.28): C, 50.62; H, 4.67; N, 17.71; S, 13.51%. Found: C, 50.66; H, 4.63; N, 17.96; S, 13.52%.

#### 3.2.4. Typical Procedure for Preparing **6a**–**c**

The aromatic amine (10 mmol) was dissolved in a mixture of HCl (29 mmol) and 12 mL of water. The solution was cooled to 0 °C in an ice bath, after which a solution of 11 mmol sodium nitrite was added into 2.4 mL of water with stirring, followed by cooling at a rate so that the temperature of the solution did not exceed 5 °C. Next, the solution was added only until slightly excess nitrous acid was obtained. The resulting solution (benzenediazonium chloride) must be stored in the ice bath and utilized fairly quickly. Thereafter, the resulting solution (aryldiazonium salt) was added into a cold solution of **1** (10 mmol) in ethanol (50 mL) containing sodium hydroxide (6 g), and the mixture was stirred for 1 h at 0 °C. The solid product was formed, filtered off, washed severally with water, and recrystallized from the solvent to afford **6a**–**c**.

#### 3.2.5. 1 3-Oxo-2-(2-phenylhydrazono)-*N*-(thiazol-2-yl)butanamide (**6a**)

Compound **6a** was obtained as yellow crystals from aqueous ethanol. Yield, 85%; m.p. = 210 °C; ^1^H NMR (CDCl_3_) δ: 2.57 (s, 3H, CH_3_), 7.01 (d, ^1^H, CH–thiazole, *J* = 3.2 Hz), 7.19–7.23 (m, 5H, ArH), 7.53 (d, 1H, CH–thiazole, *J* = 3.6 Hz), 12.63 (s, 1H, NH), 14.33 (s, 1H, NH) ppm; ^13^C NMR δ: 25.88, 114.12, 116.21, 124.50, 126.03, 129.70, 138.52, 141.30, 156.23, 161.51, 198.84 ppm; DEPT 135 ^13^C NMR δ: (+)25.86, 114.09, 116.18, 126.02, 129.64, 138.49. Anal. Calcd. for C_13_H_12_N_4_O_2_S (288.32): C, 54.15; H, 4.20; N, 19.43; S, 11.12%. Found: C, 54.17; H, 4.19; N, 19.45; S, 11.13%.

#### 3.2.6. 2 2-[2-(4-Methoxyphenyl)hydrazono]-3-oxo-*N*-(thiazol-2-yl)-butanamide(**6b**)

Compound **6b** was obtained as yellow crystals from aqueous ethanol. Yield, 87%; m.p. = 318 °C; IR (KBr): ν = 3426, 3120 (2N–H); 3018 (ArC–H); 2959 (Al C–H); 1647, 1621 (2C=O) cm^−1^; ^1^H NMR (CDCl_3_) δ: 2.54 (s, 3H, CH_3_), 3.81 (s, 3H, OCH_3_), 6.92 (d, 2H, ArH, *J* = 2 Hz), 6.94 (d, 1H, ArH, *J* = 2 Hz), 6.99 (d, 2H, CH–thiazole, *J* = 4 Hz), 7.36 (d, 1H, ArH, *J* = 2.8 Hz), 7.38 (d, 1H, ArH, *J* = 2.8 Hz), 7.52 (d, 1H, CH–thiazole, *J* = 3.2 Hz), 12.67 (s, 1H, NH), 14.42 (s, 1H, NH) ppm; ^13^C NMR δ: 25.78, 55.59, 13.92, 114.94, 117.56, 123.73, 134.97, 138.47, 156.34, 158.10, 161.74, 198.53 ppm; DEPT 135 ^13^C NMR δ: (+)25.75, 55.59, 113.92, 114.93, 117.54, 138.46. Anal. Calcd. for C_14_H_14_N_4_O_3_S (318.35): C, 52.82; H, 4.43; N, 17.60; S, 10.07%. Found: C, 52.84; H, 4.44; N, 17.61; S, 10.09%.

#### 3.2.7. 3 2-(2-(4-Chlorophenyl)hydrazono)-3-oxo-*N*-(thiazol-2-yl)-butanamide (**6c**)

Compound **6c** was obtained as yellow crystals from an aqueous ethanol. Yield, 89%; m.p. = 180 °C; IR (KBr): ν = 3418 (N–H), 3016 (ArC–H), 2923 (Al C–H), 1653 (2C=O) cm^−1^; ^1^H NMR (CDCl_3_) δ: 2.55 (s, 3H, CH_3_), 7.00 (d, 1H, CH–thiazole, *J* = 4 Hz), 7.33–7.38 (m, 4H, ArH), 7.52 (d, 1H, CH–thiazol, *J* = 3.6 Hz), 12.58 (s, 1H, NH), 14.33 (s, 1H, NH) ppm; ^13^C NMR δ: 25.88, 114.24, 117.26, 124.83, 129.80, 131.17, 138.57, 139.90, 156.05, 161.36, 198.71 ppm; DEPT 135 ^13^C NMR δ: (+)25.86, 114.22, 117.25, 129.79, 138.56. Anal. Calcd. for C_13_H_11_ClN_4_O2S (322.77): C, 48.37; H, 3.44; N, 17.36; Cl, 10.98; S, 9.93%. Found: C, 48.37; H, 3.44; N, 17.36; Cl, 10.99; S, 9.91%.

#### 3.2.8. Typical Procedure for Preparing **7a**,**b**

A few drops of piperidine (0.5 mL) and appropriate aromatic aldehyde (10 mmol) were added to a solution of **1** (1.84 g, 10 mmol) in ethanol (30 mL). The reaction mixture was heated under reflux for 6 h, and the formed product was collected by filtration, after which it was recrystallized from the proper solvent to yield **7a**,**b**.

#### 3.2.9. 1 2-Benzylidene-3-oxo-*N*-(thiazol-2-yl)butanamide (**7a**)

Compound **7a** was obtained from **1** and benzaldehyde as yellow crystals from benzene. Yield, 82%; m.p. = 197 °C; ^1^H NMR (DMSO-*d*_6_) δ: 2.11 (s, 3H, CH_3_), 7.01 (d, 1H, CH–thiazole, *J* = 7.2 Hz), 7.34–7.56 (m, 1H, Ar–H), 7.60–7.63 (m, 4H–Ar–H), 7.80 (d, 1H, CH–thiazole, *J* = 7.6 Hz), 9.04 (s, 1H, CH–aliphatic), 12.46 (s, 1H, NH) ppm; ^13^C NMR δ: 25.77, 109.66, 114.12, 116.21, 124.49, 126.03, 129.70, 138.52, 141.30, 156.17, 161.51, 198.86 ppm; DEPT 135 ^13^C NMR δ: (+)25.76, 109.66, 114.13, 116.18, 126.02, 129.70, 138.56. Anal. Calcd. for C_14_H_12_N_2_O_2_S (272.32): C, 61.75; H, 4.41; N, 10.29; S, 11.77%. Found: C, 61.73; H, 4.46; N, 10.27; S, 11.80%.

#### 3.2.10. 2 2-(4-Methoxybenzylidene)-3-oxo-*N*-(thiazol-2-yl)butanamide (**7b**)

Compound **7b** was obtained from **1** and p-methoxy-benzaldehyde as brown crystals from benzene. Yield, 56%; m.p. = 190 °C; ^1^H NMR (DMSO-*d*_6_) δ: 2.40 (s, 3H, CH_3_), 3.72 (s, 3H, OCH_3_), 6.96 (d, 2H, ArH, *J* = 8.8 Hz), 7.28 (d, 1H, CH–thiazole, *J* = 2.8 Hz), 7.46–7.50 (m, 3H, 1H–CH thiazole, 2H–ArH), 7.73 (s,1H, Ali–CH), 12.51 (s, 1H, NH) ppm; ^13^C NMR δ: 26.01, 55.48, 113.96, 114.68, 125.34, 131.19, 133.85, 137.96, 141.43, 157.64, 161.46, 166.05, 196.28 ppm; DEPT 135 ^13^C NMR δ: (+)25.93, 55.39, 113.89, 114.60, 131.84, 137.88, 141.36. Anal. Calcd. for C_15_H_14_N_2_O_3_S (302.35): C, 59.59; H, 4.67; N, 9.27; S, 10.61%. Found: C, 59.58; H, 4.65; N, 9.28; S, 10.62%.

#### 3.2.11. Typical Procedure for Preparing **8a**,**b**

A few drops of HCl (0.5 mL), thiourea (0.76 g, 10 mmol), and aromatic aldehyde (10 mmol) were added to a solution of **1** (1.84 g, 10 mmol) in ethanol (30 mL) and heated under reflux for 6 h. Thereafter, the solid product, which was produced while it was hot, was collected by filtration and recrystallized from the proper solvent to afford **8a**,**b**.

4-(4-Fluorophenyl)-6-methyl-*N*-(thiazol-2-yl)-2-thioxo-1,2,3,4-tetra-hydropyrimidine-5-carboxamide (**8a**)

Compound **8a** was obtained from **1**, p-florobenzaldehyde, and thiourea as white crystals from aqueous ethanol. Yield, 67%; m.p. = 187 °C; IR (KBr): ν = 3407 (NH); 3002 (ArC–H); 2924 (Al C-H); 1659 (C=O) cm^–1^; 1H NMR (DMSO-*d*_6_) δ: 2.15 (s, 3H, CH3), 5.54 (s, 1H, 4H-pyrimidine), 7.14–7.43 (m, 6H, 2CH–thiazole, 4–ArH), 9.60 (s, 1H, NH), 10.20 (s, 1H, NH), 11.96 (s, 1H, NH) ppm; ^13^C NMR δ: 16.88, 53.73, 104.10, 113.46, 115.45, 115.67, 128.71, 128.80, 139.06, 139.66, 160.50, 162.94, 173.88 ppm; DEPT 135 ^13^C NMR δ: (+)16.78, 53.63, 115.37, 115.57, 128.62, 128.70. Anal. Calcd. for C_15_H_13_FN_4_OS_2_ (348.42): C, 51.71; H, 3.76; N, 16.08; F, 5.45; S, 18.41%. Found: C, 51.70; H, 3.78; N, 16.06; F, 5.44; S, 18.43%.

4-(4-Methoxyphenyl)-6-methyl-*N*-(thiazol-2-yl)-2-thioxo-1,2,3,4-tetrahydropyrimidine-5-carboxamide (**8b**)

Compound **8b** was obtained from **1**, p-methoxybenzaldehyde, and thiourea as orange crystals from aqueous ethanol. Yield, 59%; m.p. = 290 °C; IR (KBr): ν = 3435 (NH); 3015 (ArC–H); 2999 (Ali–C–H); 1661 (C=O) cm^−1^; ^1^H NMR (DMSO-*d*_6_) δ: 2.14 (s, 3H, CH_3_); 3.68 (s, 3H, OCH_3_); 5.50 (s, 1H, 4H–pyrimidine); 6.87–7.43(m, 6H, 2CH–thiazole, 4ArH); 9.53 (s, 1H, NH); 10.13 (s, 1H, NH); 11.92 (s, 1H, NH) ppm; ^13^C NMR δ: 16.83, 53.88, 55.16, 104.03, 114.01, 114.07, 127.93, 127.99, 131.84, 135.00, 137.44, 139.28, 158.96, 164.63, 173.63 ppm; DEPT 135 ^13^C NMR δ: (+)17.22, 54.72, 55.69, 114.22, 114.68, 128.37, 128.73; MS (relative intensity) *m*/*z*: 362.12 (M + 2, 0.9%). Anal. Calcd. for C_16_H_16_N_4_O_2_S_2_ (360.45): C, 53.31; H, 4.47; N, 15.54; S, 17.79%. Found: C, 53.30; H, 4.45; N, 15.56; S, 17.77%.

#### 3.2.12. 4,4′-(1,4-.Phenylene)bis(6-methyl-*N*-(thiazol-2-yl)-2-thioxo-1,2,3,4-tetrahydropyrimidine-5-carboxamide) (**9**)

HCl (2 mL), terphthaldehyde (0.68 g, 5 mmol) and thiourea (0.76 g, 10 mmol) were added to a solution of **1** (1.84 g, 10 mmol) in ethanol (30 mL). The reaction mixture was heated under reflux for 12 h, after which the solid product, which was formed while it was hot, was collected by filtration and recrystallized from dioxane/ethanol to afford **9** (68%) as yellow crystals; m.p. = >300 °C; ^1^H NMR (DMSO-*d*_6_) δ: 2.10 (s, 6H, 2CH_3_), 5.45 (s, 1H, CH–pyrimidine), 5.47 (s, 1H, CH–pyrimidine), 7.13–7.26 (m, 2H, CH–thiazole), 7.25–7.42 (m, 4H, Ar–H), 7.46–7.48 (m, 2H, CH–thiazole), 9.49 (s, 1H, NH), 9.54 (s, 1H, NH), 10.14 (s, 2H, 2NH), 11.91 (s, 2H, 2NH) ppm; ^1^H NMR (DMSO-*d*_6_ + D_2_O) δ: 2.09 (s, 6H, 2CH_3_), 5.41 (s, 1H, CH–pyrimidine), 5.43(s, 1H, CH–pyrimidine), 7.09–7.45 (m, 8H, 4CH–thiazole, 4Ar–H) ppm; ^13^C NMR δ: 16.83, 54.01, 104.17, 113.54, 126.81, 135.76, 139.10, 142.54, 158.14, 164.50, 174.01 ppm; DEPT 135 ^13^C NMR δ: (+)16.73, 54.16, 113.43, 126.72, 137.60. Anal. Calcd. for C_24_H_22_N_8_O_2_S_4_ (582.74): C, 49.47; H, 3.81; N, 19.23; S, 22.01%. Found: C, 49.42; H, 3.84; N, 19.18; S, 22.05%.

#### 3.2.13. Typical Procedure for Preparing **10a**,**b**

A mixture of **1** (10 mmol), an appropriate active methylene reagent, malononitrile or ethylcyanoacetate (10 mmol), and sulfur in ethanol (20 mL) in the presence of a catalytic amount of morpholine was heated under reflux for 5 h at 55–70 °C, after which it was allowed to cool. The obtained solid product was filtered off and recrystallized from the proper solvent to afford **10a**,**b**.

5-Amino-4-cyano-2-methyl-*N*-(thiazol-2-yl)thiophene-3-carboxamide (**10a**)

Compound **10a** was obtained from **1** and malononitrile as dark brown crystals from the aqueous ethanol. Yield, 73%; m.p. = 215 °C; IR (KBr): ν = 2246.75 (C≡N) cm^−1^; ^1^H NMR (DMSO-d_6_) δ: 2.17 (s, 3H, CH_3_), 4.21 (s, 2H, NH_2_, exchangeable), 7.85 (d, 1H, CH–thiazole, *J* = 4 Hz), 7.90 (d, 1H, CH–thiazole, *J* = 3.6 Hz), 9.69 (s, 1H, NH, exchangeable) ppm; ^13^C NMR δ: 20.63, 103.77, 116.70, 123.56, 139.76, 153.17, 154.98, 155.80, 160.08, 164.87 ppm; DEPT 135 ^13^C NMR δ: (+)20.58, 123.53, 139.72; MS (relative intensity) *m*/*z*: 264.93 (M+, 12%). Anal. Calcd. for C_10_H_8_N_4_OS_2_ (264.33): C, 45.44; H, 3.05; N, 21.20; S, 24.26%. Found: C, 45.47; H, 3.04; N, 21.23; S, 24.25%.

Ethyl 2-amino-5-methyl-4-(thiazol-2-ylcarbamoyl)thiophene-3-carboxylate (**10b**)

Compound **10b** was obtained from **1** and ethylcyanacetate as brown crystals from the aqueous ethanol. Yield, 65%; m.p. = 198 °C; IR (KBr): ν = 3358 (NH), 3021 (ArC–H), 2981 (Ali–C–H), 1706 (C=O) cm^−1^; ^1^H NMR (DMSO-d_6_) δ: 1.24 (t, 3H, CH_3_, *J* = 6.8 Hz), 2.60 (s, 3H, CH_3_), 4.21 (q, 2H, CH_2_, *J* = 6.8 Hz), 7.05 (d, 1H, CH–thiazole, *J* = 4 Hz), 7.40 (d, 1H, CH–thiazole, *J* = 4 Hz), 7.82 (s, 2H, NH_2_), 12.16 (s, 1H, NH) ppm; ^13^C NMR δ: 14.34, 16.33, 59.41, 106.25, 112.01, 112.03, 132.14, 143.69, 162.02, 164.03, 165.00, 166.07 ppm; DEPT 135 ^13^C NMR δ: (+)14.35, 16.36, (−)59.42, (+)112.01, 132.14; NOE NMR δ: 12.47–18.29 (m, 2CH_3_), 57.98 (t, CH_2_), 106.27 (s), 111.01 (d, 1H, CH–thiazole), 113.03 (s), 132.99 (d, 1H, CH–thiazole), 143.67 (s), 162.13 (s), 164.08 (s), 165.03 (s), 166.10 (s); MS (relative intensity) *m*/*z*: 312.08 (M+, 1.4%). Anal. Calcd. for C_12_H_13_N_3_O_3_S_2_ (311.38): C, 46.29; H, 4.21; N, 13.49; S, 20.60%. Found: C, 46.30; H, 4.23; N, 13.47; S, 20.61%.

#### 3.2.14. Typical Procedure for Preparing **11a**–**c**

A mixture of **6a**–**c** (10 mmol) and dimethylformamide–dimethylacetal (DMF–DMA) (10 mmol) was heated under reflux for 5 min in dioxane. Thereafter, the solid that was formed upon cooling was collected and recrystallized from the proper solvents to afford (**11a**–**c**).

4-Oxo-1-phenyl-*N*-(thiazol-2-yl)-1,4-dihydropyridazine-3-carboxamide (**11a**)

Compound **11a** was obtained as brown crystals from aqueous ethanol. Yield, 65%; m.p. = 230 °C; IR (KBr): ν = 3429 (N–H), 3090 (ArC–H), 1687 (2C=O) cm^−1^; ^1^H NMR (DMSO-*d*_6_) δ: 7.01 (d, 1H, CH–pyridazine, *J* = 7.2 Hz), 7.34 (d, 1H, CH–thiazole, *J* = 1.6 Hz), 7.50–7.63 (m, 4H, ArH), 7.80 (d, 2H, ArH, *J* = 7.6 Hz), 9.04 (d, 1H, CH–pyridazine, *J* = 7.6 Hz), 13.61 (s, 1H, NH) ppm; ^13^C NMR δ: 114.89, 121.43, 121.59, 128.98, 129.80, 138.31, 142.24, 142.93, 144.46, 156.70, 158.96, 169.97 ppm; DEPT 135 ^13^C NMR δ: (+)114.84, 121.38, 121.54, 128.93, 129.75, 138.26, 142.19. Anal. Calcd. for C_14_H_10_N_4_O_2_S (298.32): C, 56.37; H, 3.38; N, 18.78; S, 10.75%. Found: C, 56.35; H, 3.36; N, 18.79; S, 10.76%.

1-(4-Methoxyphenyl)-4-oxo-*N*-(thiazol-2-yl)-1,4-dihydro-pyridazine-3-carboxamide (**11b**)

Compound **11b** was obtained as brown crystals from the aqueous ethanol. Yield, 65%; m.p. = 237 °C; IR (KBr): ν = 3420 (NH), 3089 (ArC–H), 2931 (Ali–C–H), 1689 (2C=O) cm^−1^; ^1^H NMR (DMSO-*d*_6_) δ: 3.82 (s, 3H, OCH_3_), 7.00 (d, 1H, CH–pyridazine, *J* = 8 Hz), 7.13 (d, 2H, ArH, *J* = 8.8 Hz), 7.34 (d, 1H, CH–thiazole, *J* = 3.6 Hz), 7.55 (d, 1H, CH–thiazole, *J* = 4 Hz), 7.70 (d, 2H, ArH, *J* = 8.8 Hz), 8.96 (d, 1H, CH–pyridazine, *J* = 7.6 Hz), 13.70 (s, 1H, NH) ppm; ^13^C NMR δ: 55.69, 114.73, 114.84, 121.54, 123.17, 136.42, 138.29, 142.34, 143.97, 156.70, 158.94, 159.52, 169.80 ppm; DEPT 135 ^13^C NMR δ: (+)55.69, 114.70, 114.83, 121.51, 123.15, 138.27, 142.34. Anal. Calcd. for C_15_H_12_N_4_O_3_S (328.35): C, 54.87; H, 3.68; N, 17.06; S, 9.77%. Found: C, 54.88; H, 3.66; N, 17.08; S, 9.75%.

1-(4-Chlorophenyl)-4-oxo-*N*-(thiazol-2-yl)-1,4-dihydro-pyridazine-3-carboxamide (**11c**)

Compound **11c** was obtained as brown crystals from the aqueous ethanol. Yield, 67%; m.p. = 295 °C; IR (KBr): ν = 3431 (NH), 3099 (ArC–H), 1688 (2C=O) cm^−1^; ^1^H NMR (DMSO-*d*_6_) δ: 7.012 (d, 1H, CH–pyridazine, *J* = 7.6 Hz), 7.35 (d, 1H, CH–thiazole, *J* = 3.2 Hz), 7.56 (d, 1H, CH–thiazole, *J* = 2.8 Hz), 7.68 (d, 2H, ArH, *J* = 8.4 Hz), 7.84 (d, 2H, ArH, *J* = 8.4 Hz), 9.03 (d, 1H, CH–pyridazine, *J* = 8 Hz), 13.53 (s, 1H, NH) ppm; ^13^C NMR δ: 114.91, 121.28, 123.28, 129.70, 133.37, 138.31, 141.66, 142.08, 144.64, 156.70, 158.89, 169.92 ppm; DEPT 135 ^13^C NMR δ: (+)114.88, 121.23, 123.27, 129.67, 138.26, 142.06. Anal. Calcd. for C_14_H_9_ClN_4_O_2_S (332.76): C, 50.53; H, 2.73; N, 16.84; Cl, 10.65; S, 9.64%. Found: C, 50.56; H, 2.72; N, 16.85; Cl, 10.64; S, 9.65%.

#### 3.2.15. *N*-(5-(4-Chlorophenyl)-5*H*-pyrazolo[4,3-c]pyridazin-3-yl)-thiazol-2-amine (**12**)

A mixture of **11c** (3.32 g, 10 mmol) and hydrazine hydrate (0.6 g, 15 mmol) was fused for 5 min in a round-bottomed flask. Thereafter, pyridine (30 mL) was added, and the mixture was heated under reflux for 12 h. Afterward, it was allowed to cool. The obtained solid product was filtered off and recrystallized from the aqueous ethanol to afford **12** (68%) as yellow crystals. m.p = 157 °C; IR (KBr): ν = 3267 (N–H); 3015 (ArC–H) cm^−1^; ^1^H NMR (DMSO-*d*_6_) δ: 7.07 (d, 1H, CH–thiazole, *J* = 1.2 Hz) 7.24 (d, 1H, CH–pyridazine, *J* = 2.4 Hz), 7.37 (d, 2H, ArH, *J* = 8.4 Hz), 7.52 (d, 1H, CH–pyridazine, *J* = 2.8 Hz), 7.56 (d, 2H, ArH, *J* = 8.4 Hz), 7.96 (d, 1H, CH–thiazole, *J* = 1.6 Hz), 13.04 (s, 1H, NH) ppm; ^13^C NMR δ: 106.49, 109.86, 113.69, 116.44, 125.26, 125.92, 129.24, 137.86, 142.08, 144.72, 158.30, 162.99 ppm; DEPT 135 ^13^C NMR δ: (+)106.30, 109.66, 113.53, 116.26, 129.06, 137.68; MS; *m*/*z* = 329.76 (M + 1). Anal. Calcd. for C_14_H_9_ClN_6_S (328.78): C, 51.14; H, 2.76; N, 25.56; Cl, 10.78; S, 9.75%. Found: C, 51.16; H, 2.77; N, 25.54; Cl, 10.77; S, 9.76%.

#### 3.2.16. (Z)-*N*-(5-(4-Chlorophenyl)-2-phenyl-2*H*-pyrazolo[4,3-c]-pyridazin-3(5*H*)-ylidene)thiazol-2-amine (**13**)

A mixture of **11c** (3.32 g, 10 mmol) and phenylhydrazine (1.08 g, 10 mmol) was fused for 5 min, after which acetic acid (30 mL) containing 2 g of sodium acetate was added before the mixture was heated under reflux for 12 h. Afterward, it was allowed to cool before it was poured into ice-cold water. The obtained solid product was filtered off and recrystallized from the aqueous ethanol to afford **13** (54%) as pink crystals. m.p. = 320 °C; IR (KBr): ν = 3046 (ArC–H) cm^−1^; ^1^H NMR (DMSO-*d*_6_ + CDCl_3_) δ: 7.15–7.19 (m, 1H, ArH), 7.37–7.42 (m, 3H; 1H–CH pyridazine, 2H–CH thiazole), 7.55 (d, 2H, ArH, *J* = 8.8 Hz), 7.79 (d, 2H, ArH, *J* = 8.8 Hz), 8.06–8.12 (m, 4H, Ar–H), 8.52 (d, 1H, CH–pyridazine, *J* = 8 Hz) ppm; ^13^C NMR δ: 111.22, 113.94, 115.78, 118.88, 122.76, 125.13, 128.53, 129.50, 133.32, 133.60, 134.06, 138.95, 141.68, 141.83, 147.44, 157.25 ppm; DEPT 135 ^13^C NMR δ: (+)111.26, 113.99, 118.91, 122.79, 125.19, 128.57, 129.54, 133.62, 147.46; MS (relative intensity) *m*/*z*: 405.88 (M + 1, 9.1%). Anal. Calcd. for C_20_H_13_ClN_6_S (404.88): C, 59.33; H, 3.24; N, 20.76; Cl, 8.76; S, 7.92%. Found: C, 59.31; H, 3.26; N, 20.74; Cl, 8.78; S, 7.93%.

### 3.3. Pharmacological Assessment

#### 3.3.1. PDE5 Enzyme Activity Assay Procedure

The standard enzymatic reaction mixture (total volume, 200 µL) contained 100 mL of mTris-HCl buffer (pH 8.3), 10 mM MgCl_2_, and 10 mM KCl at 37 °C. Alfa-casein (2 mg) was utilized as a carrier for the precipitation of protein at a low concentration (PDE-A1) of enzyme sample (final protein concentration, 0.5 mg/mL). A concentration (104 nM) of the agents under study (the sildenafil analogs) was prepared in DMSO and pre-incubated in the enzymatic mixture for 5 min at room temperature. The reaction was initiated by the addition of the substrate, cGMP (5 µM) for 30 min at 35 °C. The reaction was terminated by the transfer of the reaction mixture tubes into a boiling water bath for 3 min. Thereafter, the sample was centrifuged and filtered through a nylon-66 filter (0.2 mm, Rainin Corporation). The clear filtrate obtained might be utilized directly for the high-performance liquid chromatography assay or stored at −20 °C. A blank containing protein, which was denaturized in the boiling water bath for 3 min, was performed with and without cGMP. Both the incubation time and enzyme concentration were adjusted to ensure that no more than 25% of the substrate was hydrolyzed under the assay conditions. The chromatographic system was G1315D DAD (Agilent Technologies 1200 Series, Santa Carla, California, USA). The utilized column was Zorbax Eclipse with rapid resolutions (4.69 mm × 150 mm, 3.5l M particle size). The mobile phase employed for the separation (isocratic elution) consisted of 200 mM ammonium acetate (pH 6.0) with 2% acetonitrile (*v*/*v*). The flow rate was 1.5 cm^3^·min^−1^ with diode-array detection (DAD) at 254 nm, and the injection volume was 30 mm^3^. The peak identities were confirmed by the co-elution with the standards. All the assays were performed in duplicate [20,27].

#### 3.3.2. ABP Measurement

Regardless of the sex factor, white New Zealand rabbits weighing 2–2.5 kg were obtained from the animal house of the Faculty of Medicine, Assiut University. The animals were housed for one week to adapt to the environmental conditions. The rabbits were fed with a standard diet and allowed free access to water. The experiment was performed according to the accepted guidelines for animal care. Sixty-four normotensive white New Zealand rabbits (2–2.5 kg) of either sex were utilized for this assay (four rabbits in each group) to evaluate the possible vasorelaxant effects of the selected compounds. The external jugular vein was also tightly cannulated for the administration of the tested compound in a dose of 0.5 mg/kg, followed by heparinized saline (0.90% (*w/v*) NaCl). The effect on the MABP appeared within one minute. The normotensive rabbits were anesthetized with pentobarbital sodium (30 mg/kg, IV). Thereafter, they were laid on their backs with their legs fixed and their heads pinned. Next, their tracheas were exposed and cut before they were firmly cannulated. Following the separation, the two muscle bundles, the sternomastoid and the sternothyroid, which are the common carotid arteries in the neck between the lateral bundles of muscle (longus capitis) and the trachea, were exposed and carefully separated from the nerves, veins, and connective tissue. A heparinized cannula was carefully inserted and firmly bound with a fine thread. Thereafter, the cannula was connected to a pressure transducer utilizing a universal oscillograph (Harvard apparatus, ser. No. K10542) for recording the blood pressure. The external jugular vein was also tightly cannulated for the administration of the tested compounds, followed by heparinized saline (0.90% (*w*/*v*) NaCl) [20].

MABP was calculated by the following formula: MABP = DBP + 1/3 (SBP − DBP), where DBP is the diastolic blood pressure, SBP is the systolic blood pressure.

The statistical analysis was conducted with the GraphPad Prism 5.0 software (GraphPad, San Diego, CA, USA). The data were represented as the mean ± SE. Further, multiple comparisons were conducted by the one-way ANOVA employing the Tukey–Kramer test as the multiple comparison post-ANOVA test. *p* < 0.05 was selected as the statistical significance.

### 3.4. Molecular Modeling

#### 3.4.1. Lead Optimization by Scaffold Hopping

The molecular shape and electrostatic similarity were determined by the OpenEye EON scientific software by replacing the whole molecules with the reference drug (sildenafil) to ensure the achievement of physically realistic electrostatic potential comparisons (OpenEye, 2020b). EON significantly contributes to lead generation and library design.

#### 3.4.2. Structure-Based Lead Discovery

The designed and synthesized compounds were docked into PDE5 (PDB ID: 2H42) [29] by an OpenEye-applications-2020-Spring released software. To validate our study, the docking protocol commenced with sildenafil as the standard ligand that was co-crystallized with 2H42. The designed compounds were first subjected to a filtering process utilizing the OpenEye filter-4.0.0.4 application [33], which calculates MW, XlogP, XlogS, PSA, the hydrogen-bond donor and acceptor counts, rotatable bonds, ring size and number, etc. Moreover, the filtering process was assigned a graph-based protonation state for consistency and speed; it affords ADME filters, such as Lipinski, Egan, Veber, and Martin. The compounds with impossible bonding and inappropriate elements were removed. Thereafter, the library was processed through the OpenEye Omega2-4.0.0.4 application in which the 3D conformations of the molecules were generated with an extension of the OBinary file. Next, the OpenEye docking (OEDocking) was performed by the FRED application [31,33]. The FRED-3.5.0.4 2020 spring released OpenEye docking application employs chemgauss4 as a state-of-the-art scoring function, which includes the total scores of the favorable (shape, hydrogen bonding, and metal chelation) and unfavorable (desolvation and clash) interactions [34,35,36,37]. Finally, the results were displayed by the OpenEye VIDA-4.4.0 application [33].

## 4. Conclusions

In this study, new heterocycle-based 2-aminothiazole derivatives with amide functionalities were designed, synthesized, and biologically evaluated for their activity against PDE5. The amide group contributed to the formation of HB with the essential GLN:817:A. Compounds **2**, **5**, **10a**, and **11b** exhibited 100% PDE5 inhibition compared to sildenafil utilizing the same dose. The effects of the compounds on MABP were also examined, and **10a** and **11b** exerted significant inhibitory effects without severe hypotension. Compound **11b** slightly produced hypotension effect while compounds **5** and **10a** increased the mean arterial blood pressure and may be attributed to the degree of selectivity toward different subtype and isoform of phosphodiesterase or maybe compounds **5** and **10a** have an additional molecular mechanism beside the inhibition of phosphodiesterase enzyme in the blood vessel. This pushes us urgently to investigate the exact molecular mechanism of these two compounds in the blood vessels in the next work. These positive results are a cornerstone for the designs of more potent derivatives as PDE5 inhibitors for the safe treatment of ED.

## Figures and Tables

**Table 1:** Effect of tested compounds on PDE5 (10^4^ nM) and MABP.

Compound No.	PDE5 (Inhibition %)	MABP
**1**	13	103.1 ± 1.85 ^#^
**2**	100	64.7 ± 2.98 ^###^
**3**	2	79.3 ± 2.57 ^##^
**5**	100	115.95 ± 2.91 ^###^
**6a**	20	110.1 ± 3.5 ^##^
**6c**	5	108.8 ± 3.9 ^#^
**7a**	38	87.2 ± 2.68 *
**7b**	62	90.4 ± 2.84 **
**8b**	23	88.1 ± 1.62 **
**9**	10	90.1 ± 1.62 **
**10a**	100	110.3 ± 2.84 ^#^
**10b**	82	105.3 ± 2.84 ^#^
**11a**	8	120.1 ± 2.96 ^###^
**11b**	100	78.3 ± 2.57 ^##^
**12**	7	90.3 ± 1.43 **
**11c**	13	81.3 ± 2.57 ^##^
**13**	8	81.10 ± 3.78 ^#^
Control	-	93.10 ± 1.55
Nitroprusside	-	52.10 ± 1.11 ^###^
Sildenafil	100	72.50 ± 2.92 ^##^

Data were expressed as means ± SE (n = 4). Multiple comparisons were done using one-way ANOVA followed by Tukey–Kramer as post-ANOVA test. **^#^**: significantly different from control group at *p* < 0.05. **^##^**: significantly different from control group at *p* < 0.05. **^###^**: significantly different from control group at *p* < 0.05. *: significantly different from sildenafil group at *p* < 0.05. **: significantly different from sildenafil group at *p* < 0.05.

**Table 2:** The OpenEyes’s EON scores of target compounds against sildenafil as a reference drug.

Compound No.	EON ET Combo	EON Rank
Sildenafil	0.494	1
**9**	0.370	2
**5**	0.298	3
**6a**	0.290	4
**12**	0.247	5
**8a**	0.235	6
**13**	0.234	7
**6b**	0.203	8
**11b**	0.198	9
**11a**	0.186	10
**8b**	0.181	11
**6c**	0.167	12
**7a**	0.147	13
**7b**	0.136	14
**2**	0.129	15
**11c**	0.115	16
**10a**	0.107	17
**10b**	0.106	18
**3**	0.041	19

**Table 3:** FRED Chemgauss4 scores for the docked compounds.

Comp. No	FRED Chemgauss4 Score
**12**	−12.9335
**3**	−12.3931
**11b**	−11.3665
**8b**	−11.1262
**11c**	−11.0972
**11a**	−10.9461
**7a**	−10.9088
**10b**	−10.1529
**8a**	−9.7921
**10a**	−9.594
**7b**	−9.0632
**2**	−8.4895

## Data Availability

The data presented in this study are available in Supplementary Material.

## Supplementary Materials

The following are available online, Molecular modeling investigation along with the copies of spectral data for the synthesized compounds are provided in the supplementary materials.

## Sample Availability

Samples of the compounds **1**–**13** are available from the authors.
