# Supplementary material for: Multifunctional Isosteric Pyridine Analogs-Based 2-Aminothiazole: Design, Synthesis, and Potential Phosphodiesterase-5 Inhibitory Activity
Source: Molecules. 2021 Feb 9;26(4):902. doi: 10.3390/molecules26040902 (PMC7915674; doi:10.3390/molecules26040902)
Supplement: Supplementary file 1 [file molecules-26-00902-s001.pdf]

Supporting Information

Multifunctional Isosteric Pyridine Analogs-Based 2-Aminothiazole: Design, Synthesis, and Potential Phosphodiesterase-5 Inhibitory Activity

Abdel Haleem M. Hussein [1], Ahmed A. Khames [1], Abu-Bakr A. El-Adasy [1], Ahmed A. Atalla [1], Mohamed Abdel-Rady [2], Mohamed I. A. Hassan [3], Mahrous A. Abou-Salim [4], Yaseen A. A. M. Elshaier [5,*] and Assem Barakat [6,7,*]

[1] Department of Chemistry, Faculty of Science, Al-Azhar University, Assiut 71524, Egypt. abdelhaleemmh@yahoo.com (A.H.M.H.); a.khames@yahoo.com (A.A.K); a_eladasy@azhar.edu.eg (A-B.A.E-A.); ahmedswify54@yahoo.com (A.A.A.)

[2] Department of Chemistry, Faculty of Science, Assiut University, Assiut 71516, Egypt. mohamedrady2004@yahoo.com (M.A-R.)

[3] Department of Pharmacology & Toxicology, Faculty of Pharmacy, Al-Azhar University, Assiut 71524, Egypt. mohamed_aa74@yahoo.com (M.I.A.H.)

[4] Department of Pharmaceutical Organic Chemistry, Faculty of Pharmacy, Al-Azhar University, Assiut 71524, Egypt. mahrousabousalim@azhar.edu.eg (M.A.A.)

[5] Department of Organic and Medicinal Chemistry, Faculty of Pharmacy, University of Sadat City, Menoufiya 32958, Egypt.

[6] Department of Chemistry, College of Science, King Saud University, P. O. Box 2455, Riyadh 11451, Saudi Arabia

[7] Department of Chemistry, Faculty of Science, Alexandria University, Alexandria 21321, Egypt.

* Correspondence: yaseenorganic@yahoo.com & yaseen.elshaier@fop.usc.edu.eg (Y.A.M.M.E.); ambarakat@ksu.edu.sa (A.B.)

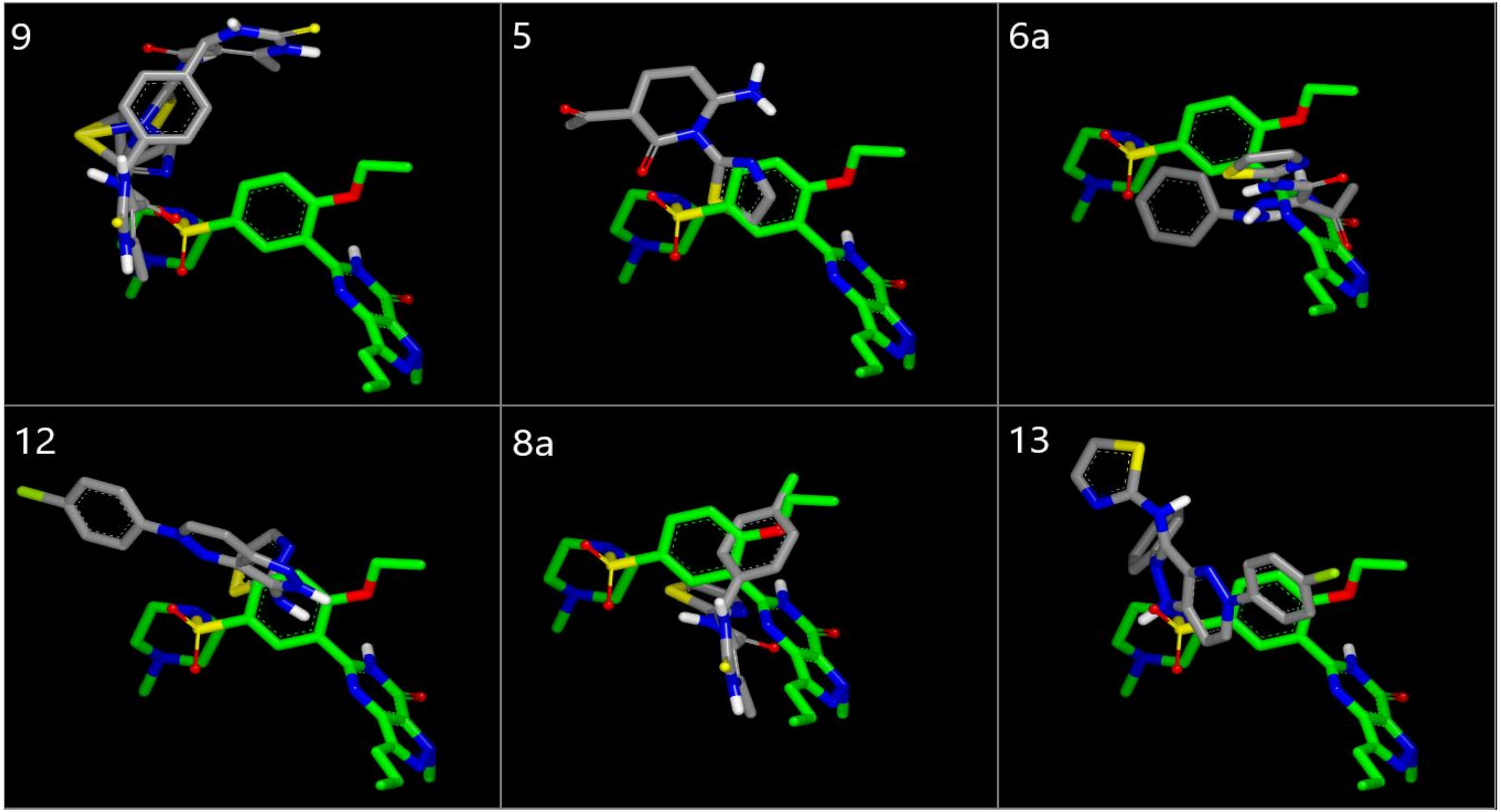

Figure S1. Scaffold hopping lead optimization (EON view); Shapes and electrostatic potentials of compounds **5, 6a, 8a, 9, 12** and **13** to the reference drug sildenafil.

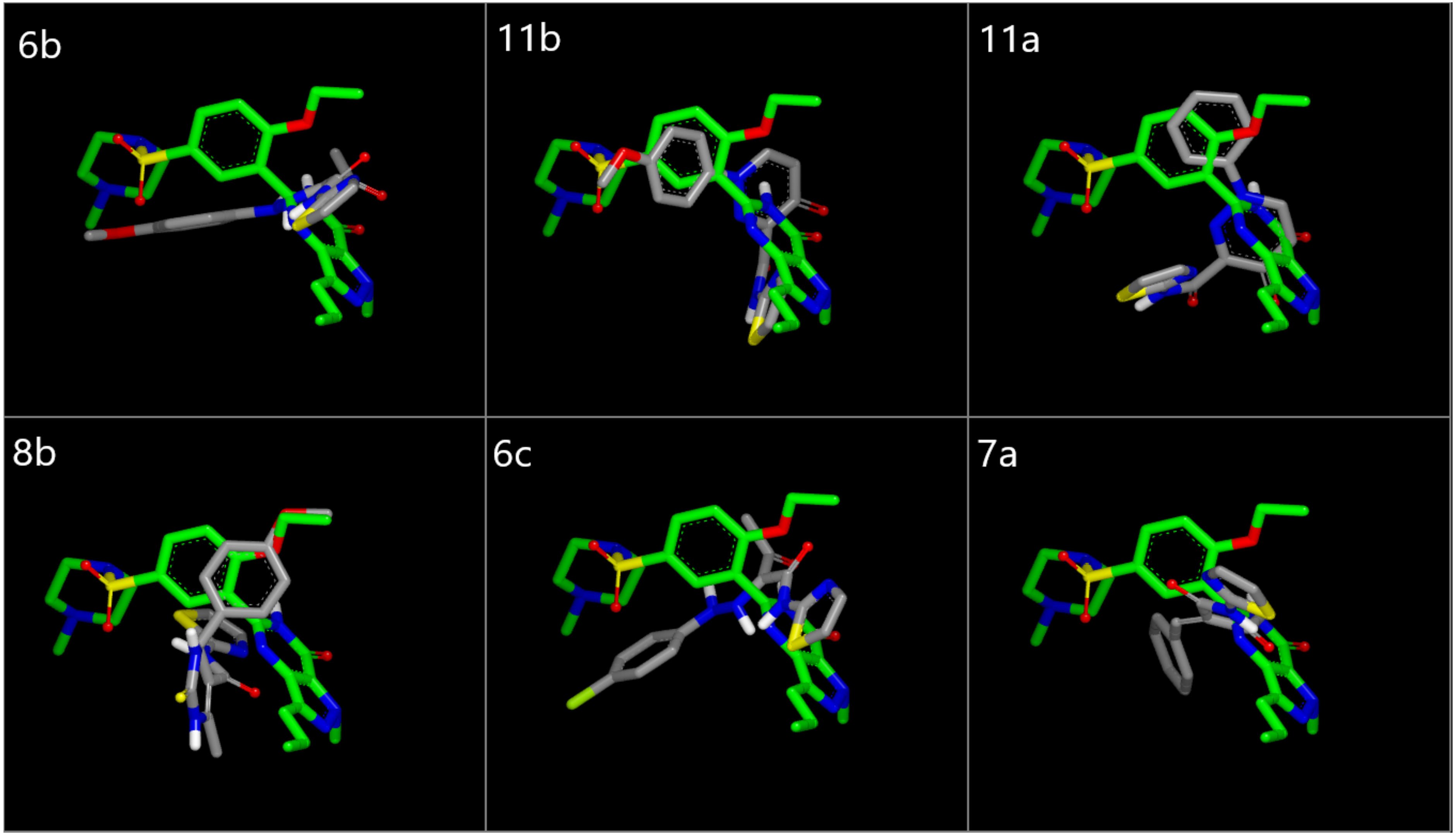

Figure S2. Scaffold hopping lead optimization (EON view); Shapes and electrostatic potentials of compounds **6b, 6c, 7a, 8b, 11a** and **11b** to the reference drug sildenafil.

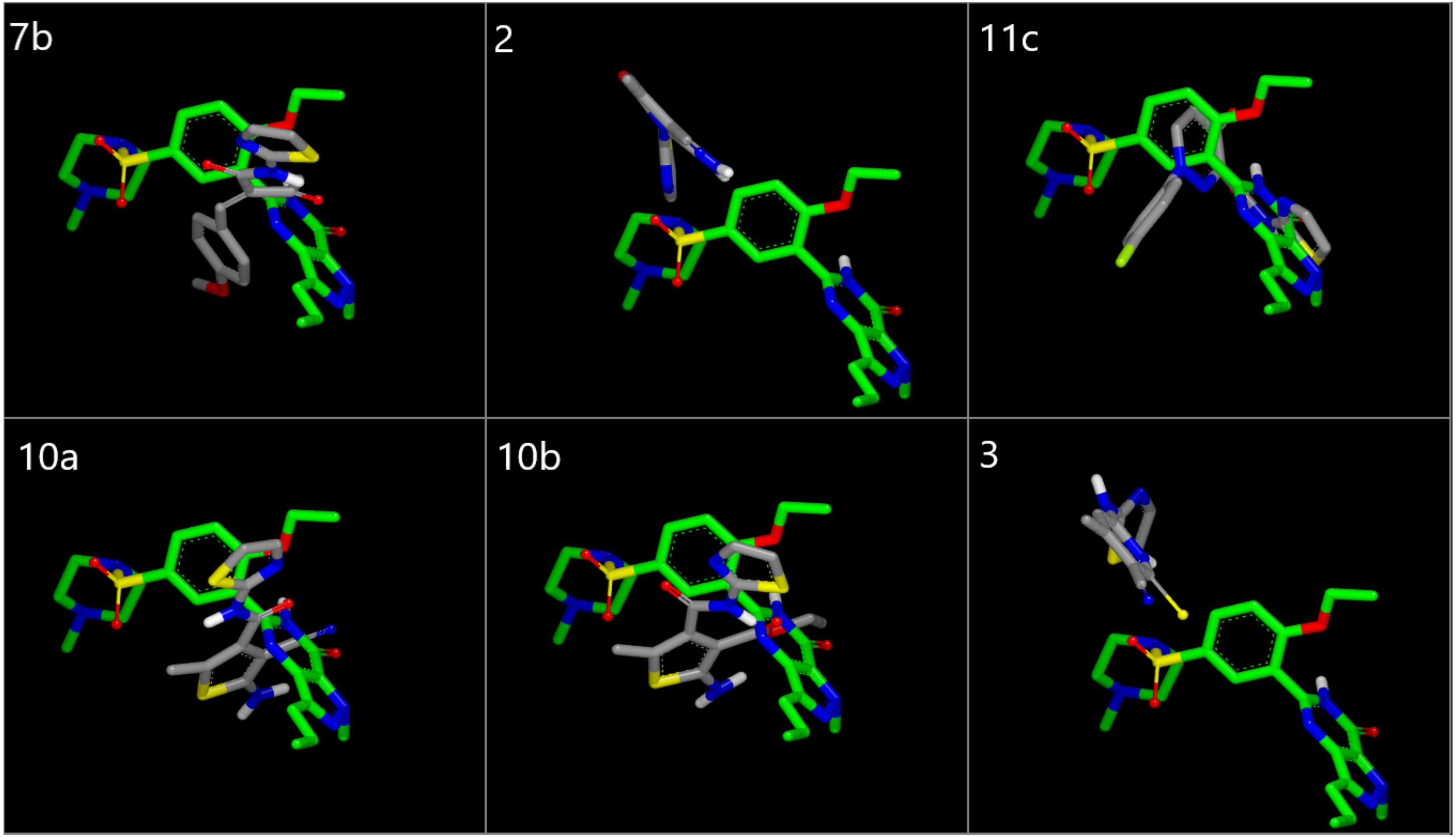

Figure S3. Scaffold hopping lead optimization (EON view); Shapes and electrostatic potentials of compounds **2, 3, 7b, 10a, 10b** and **11c** to the reference drug sildenafil.

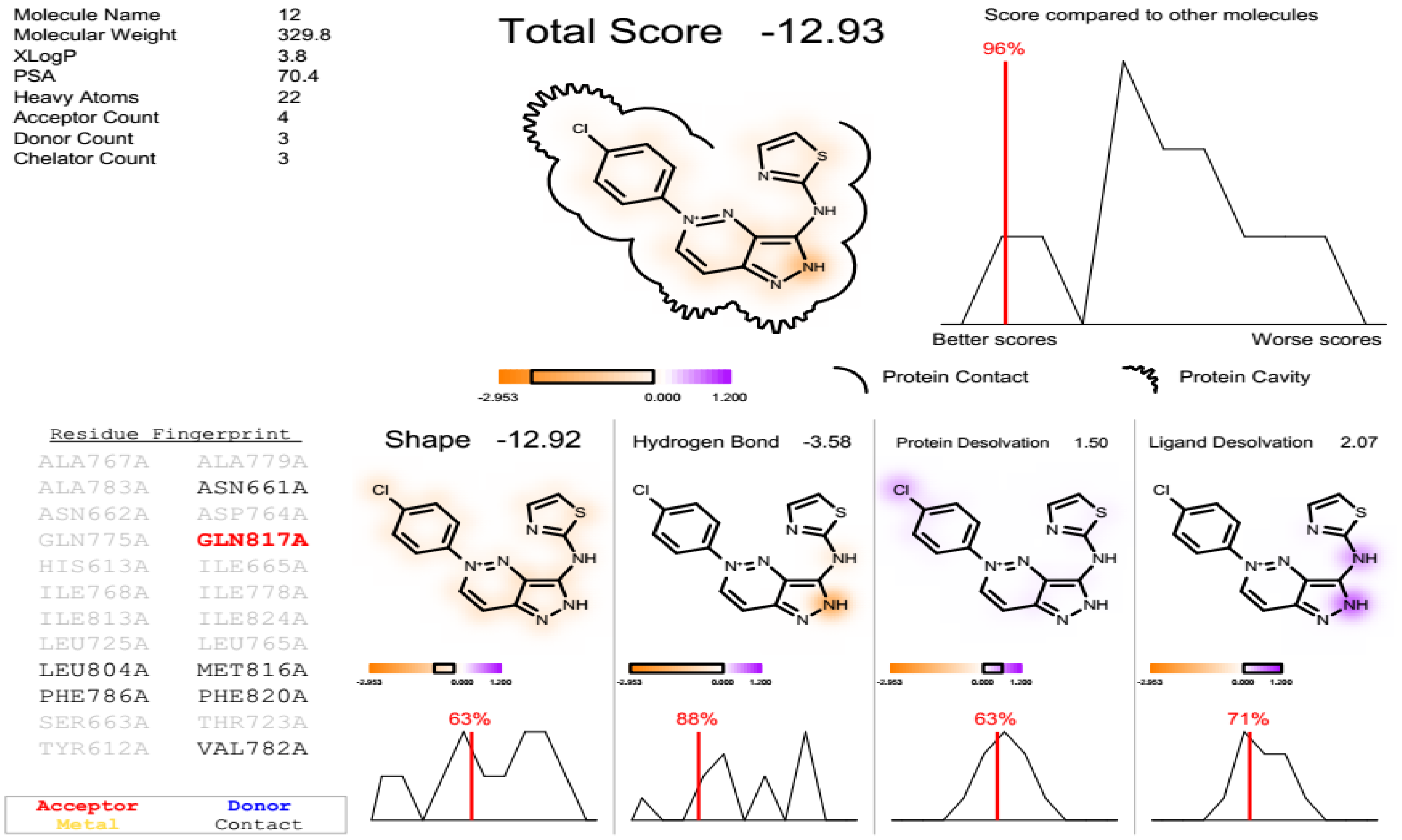

Figure S4. Molecular modeling study: Fred view of compound **12**.

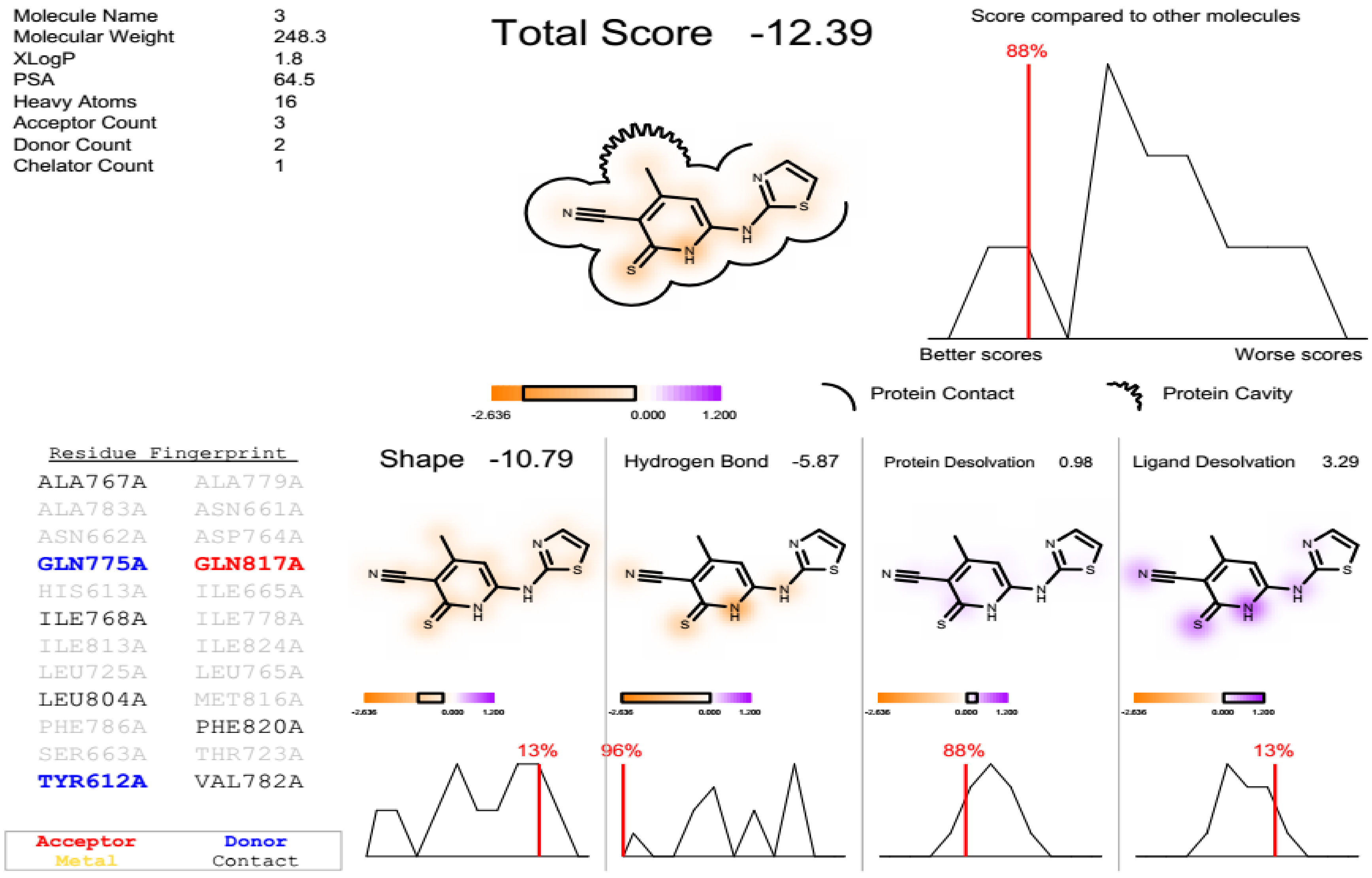

Figure S5. Molecular modeling study: Fred view of compound **3**.

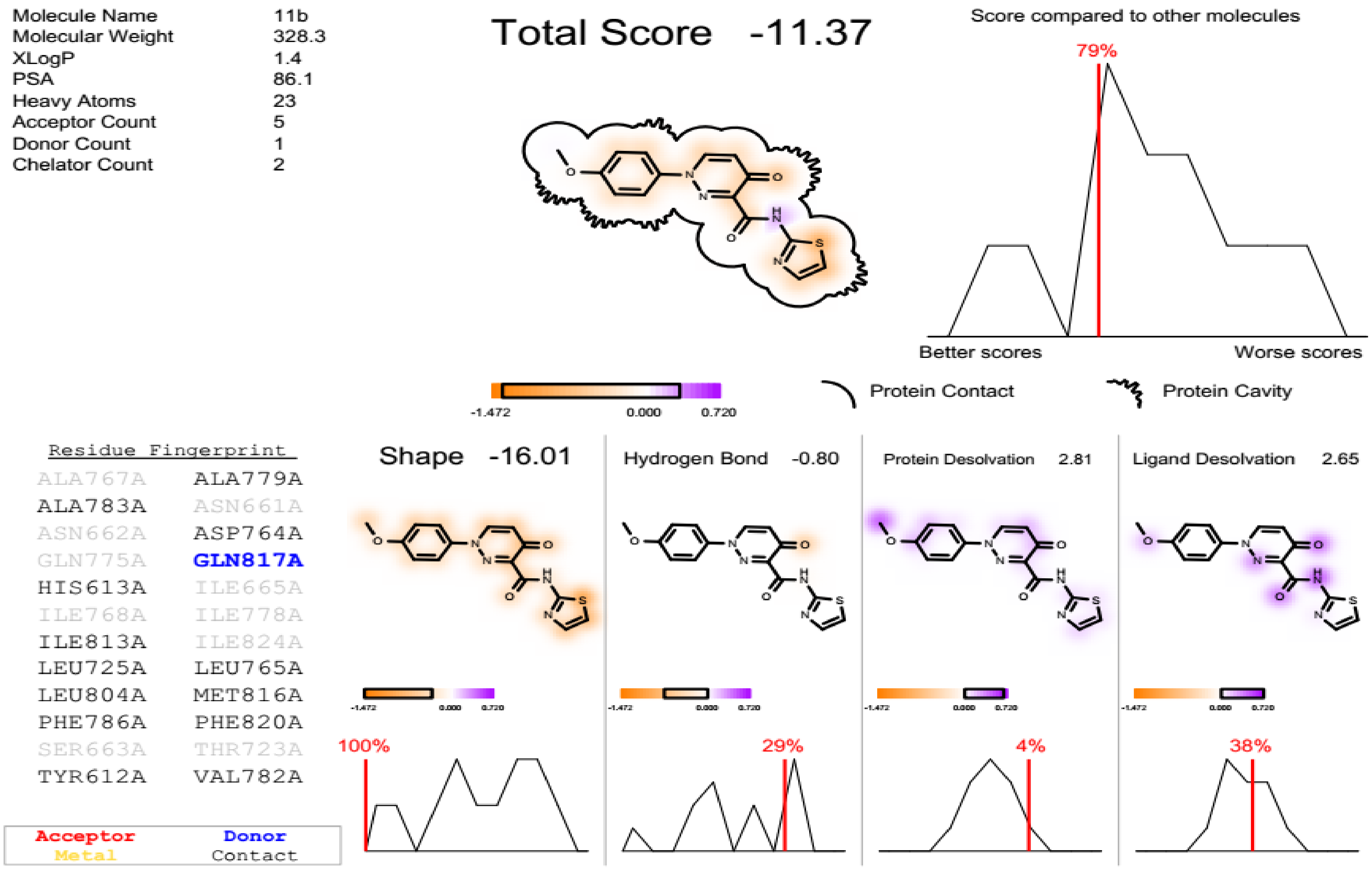

Figure S6. Molecular modeling study: Fred view of compound **11b**.

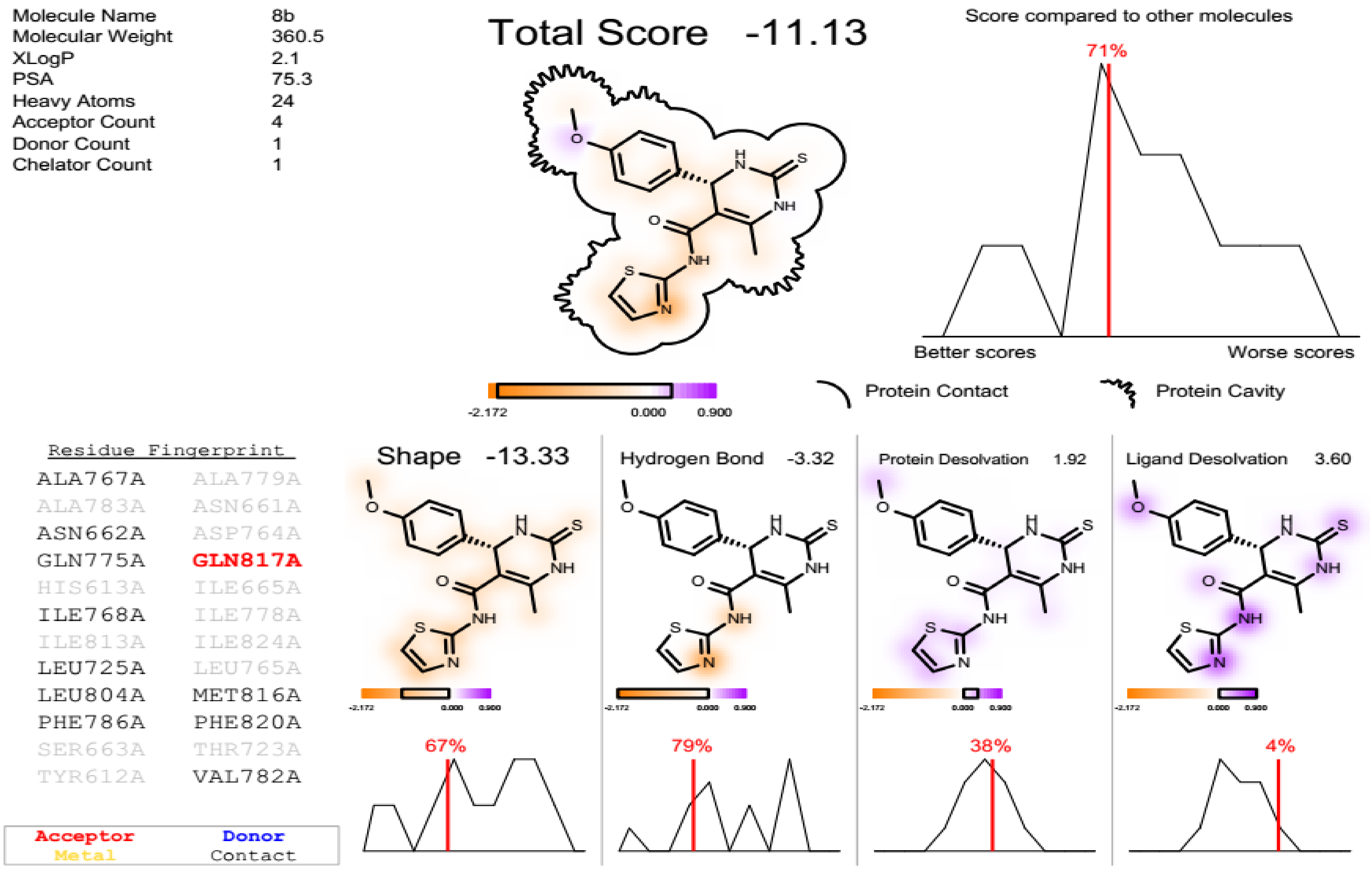

Figure S7. Molecular modeling study: Fred view of compound **8b**.

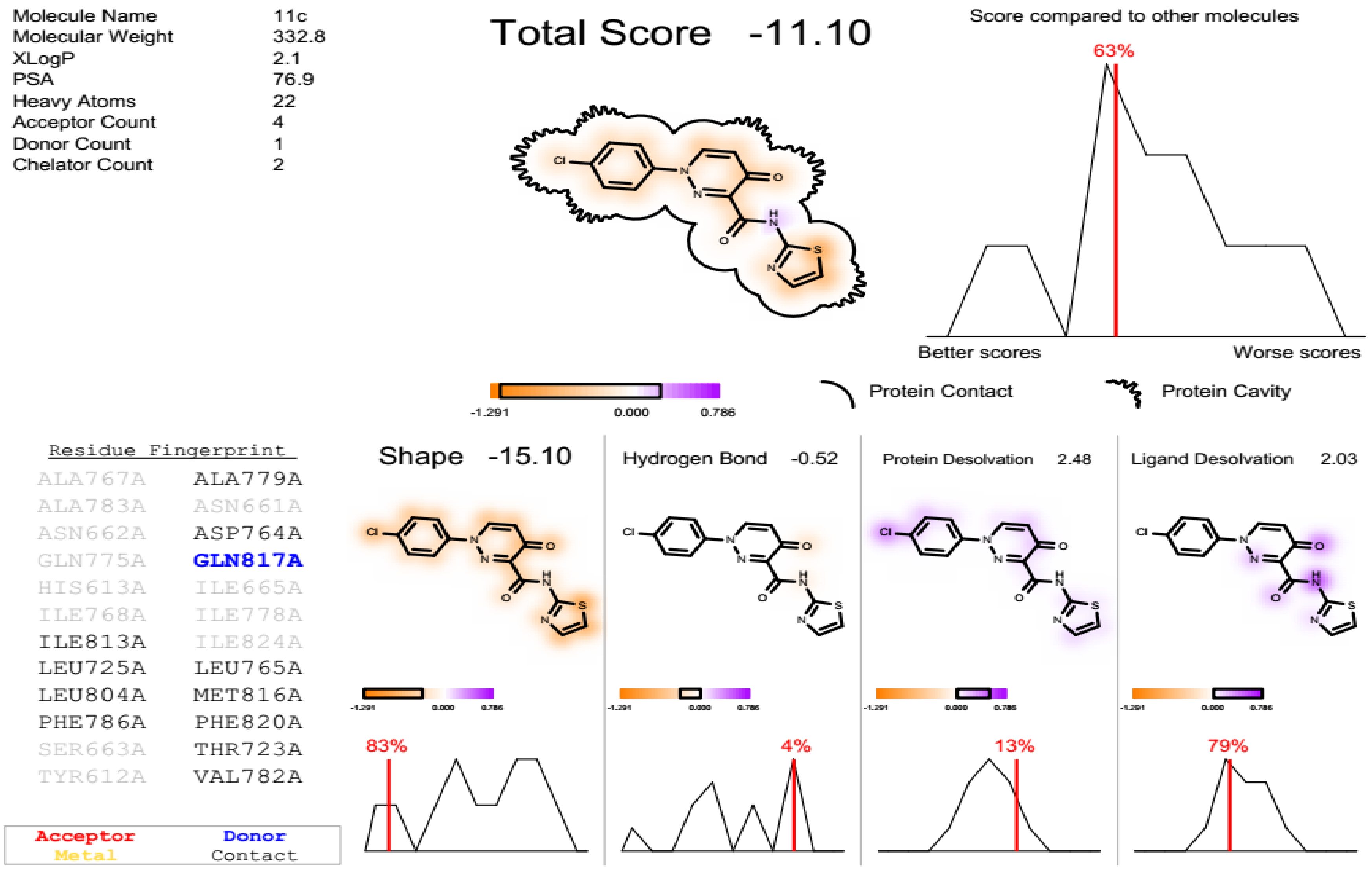

Figure S8. Molecular modeling study: Fred view of compound **11c**.

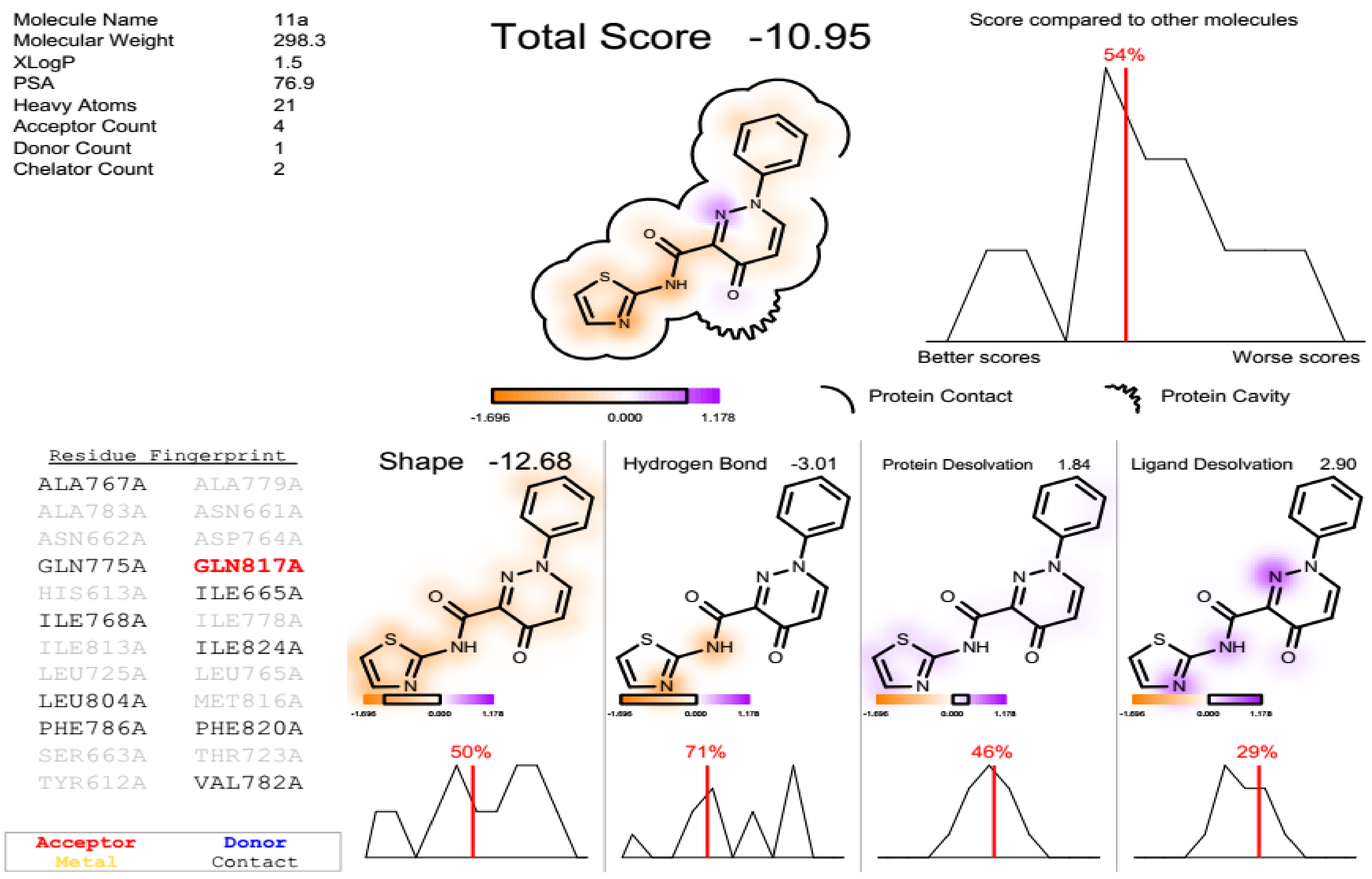

Figure S9. Molecular modeling study: Fred view of compound **11a**.

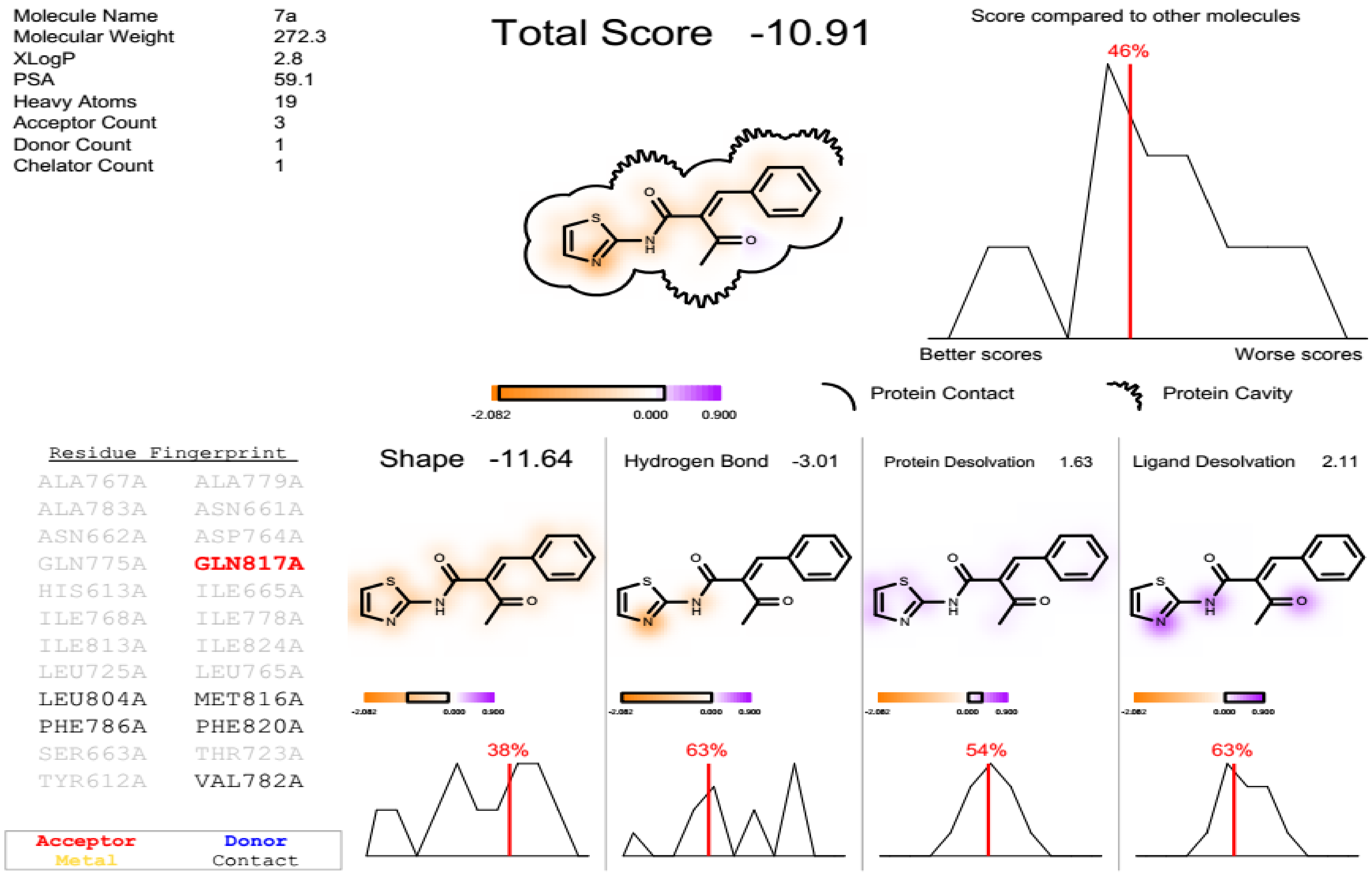

Figure S10. Molecular modeling study: Fred view of compound **7a**.

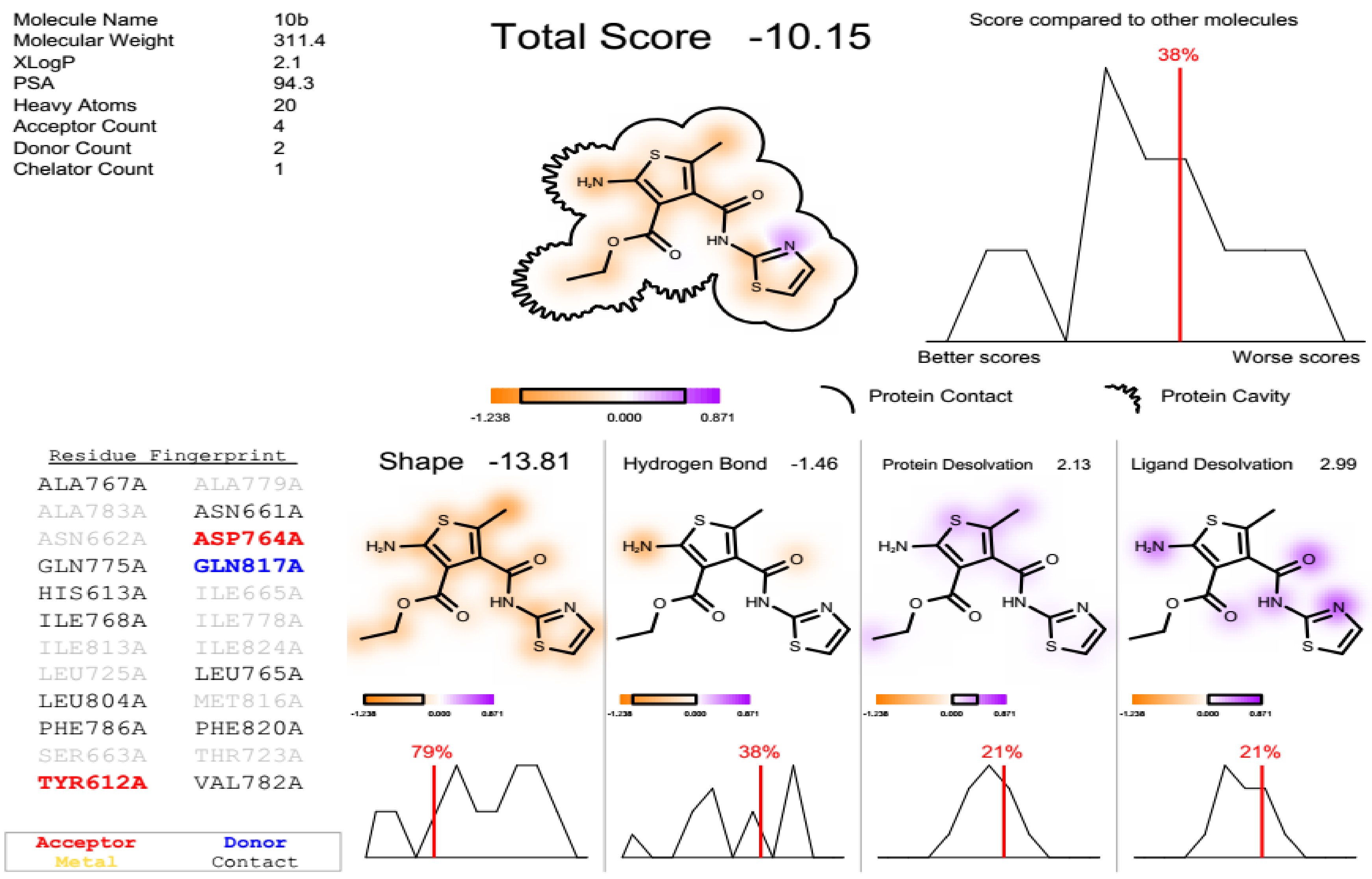

Figure S11. Molecular modeling study: Fred view of compound **10b**.

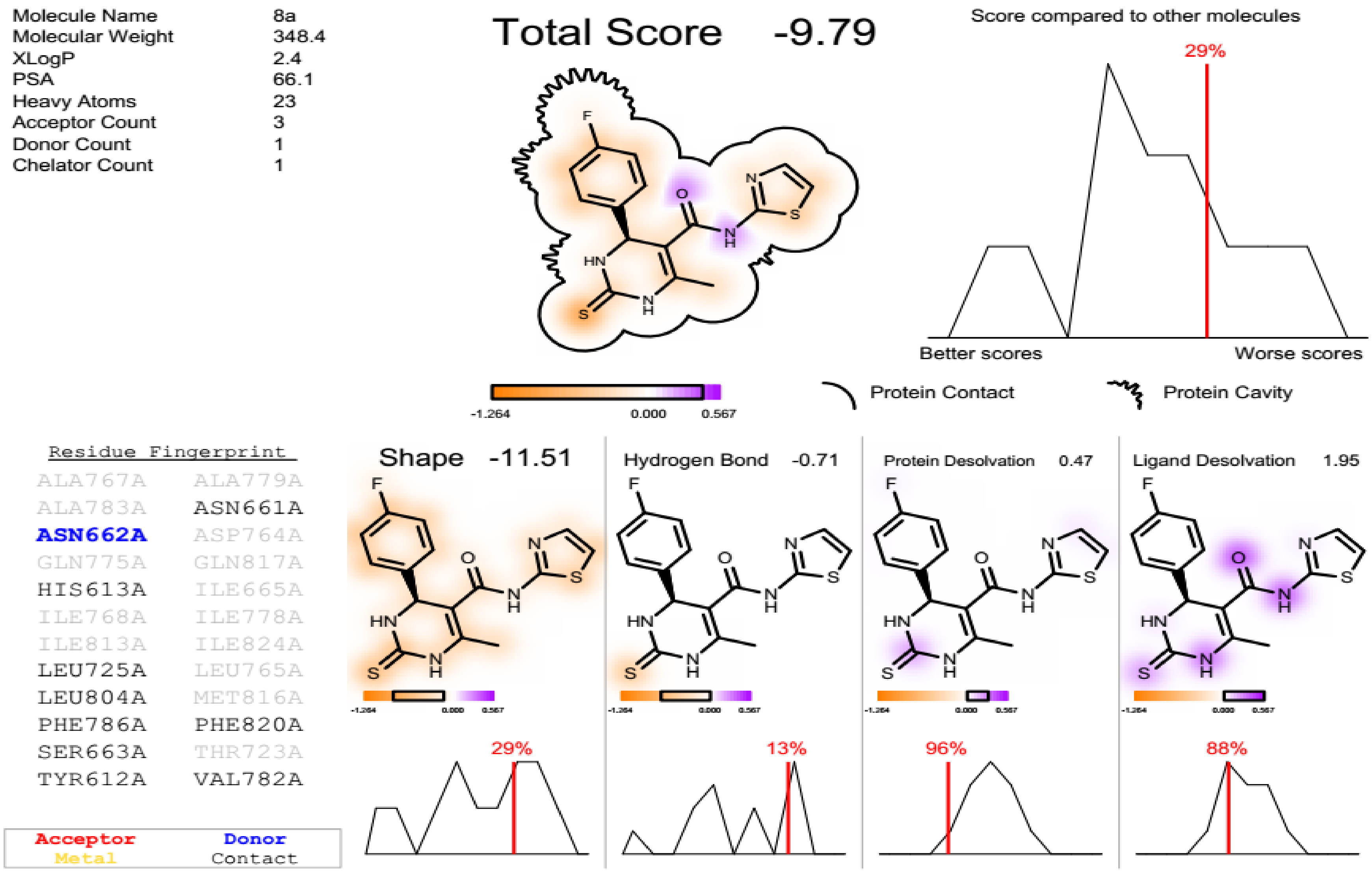

Figure S12. Molecular modeling study: Fred view of compound **8a**.

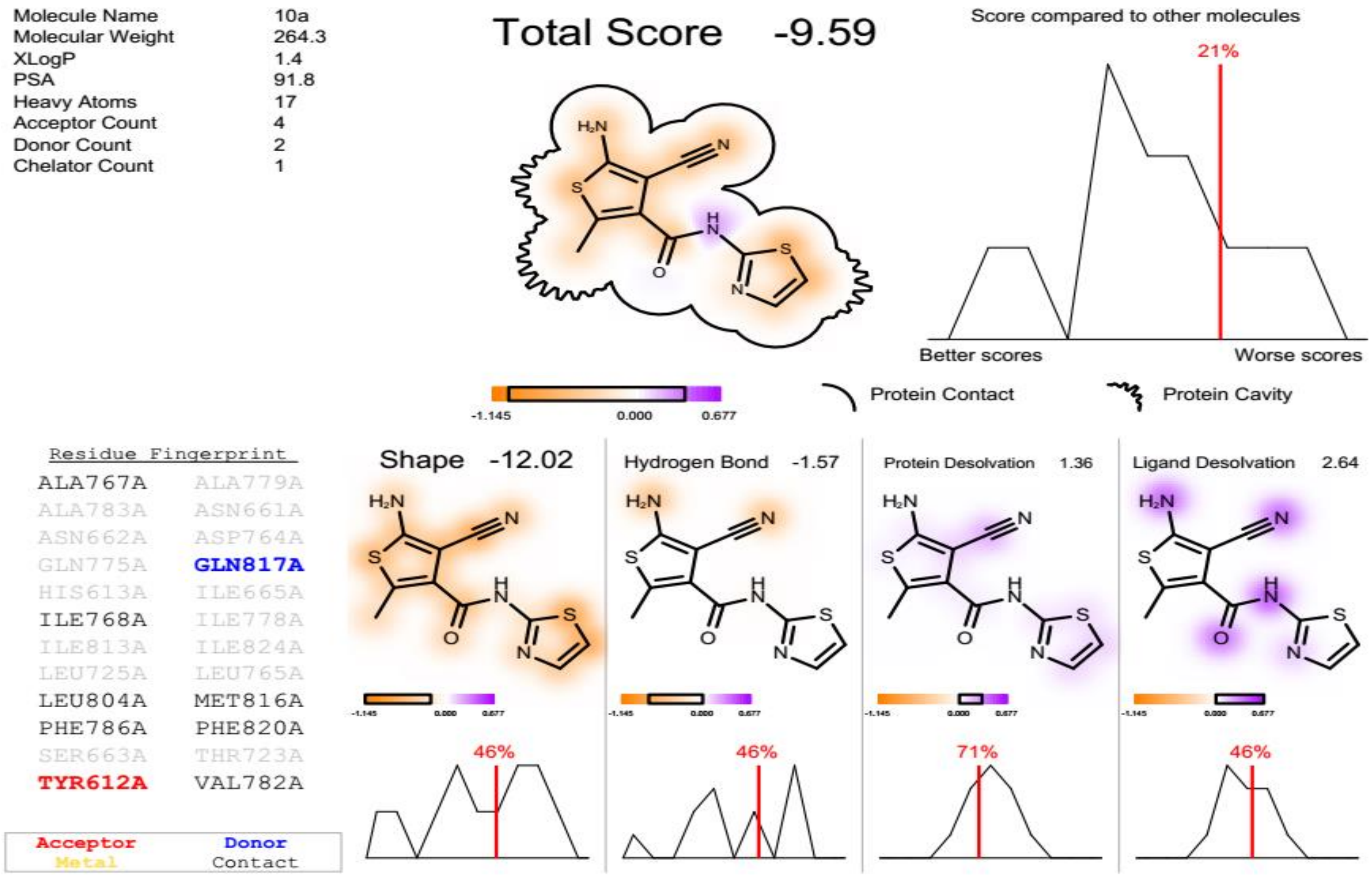

Figure S13. Molecular modeling study: Fred view of compound **10a**.

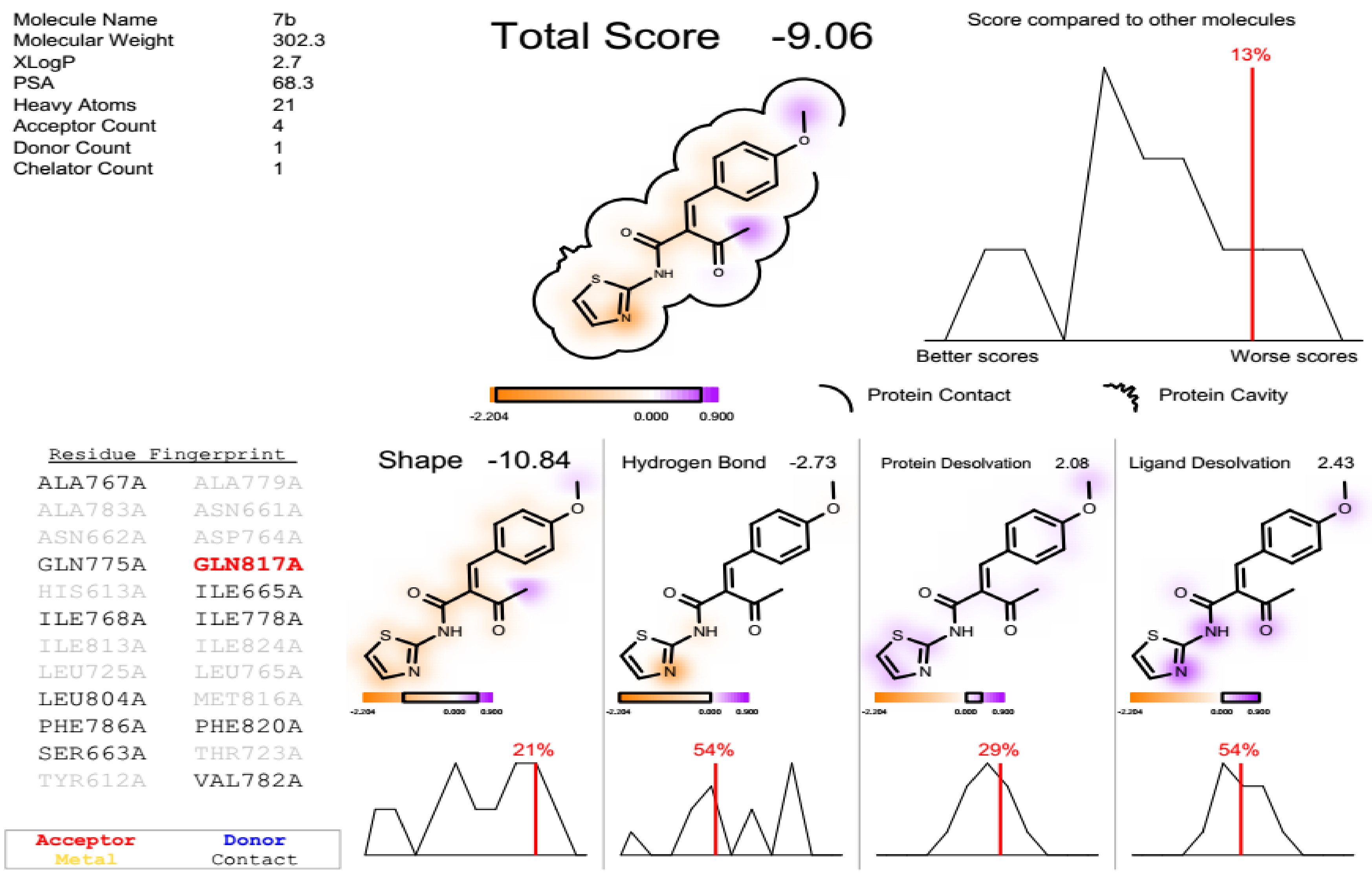

Figure S14. Molecular modeling study: Fred view of compound **7b**.

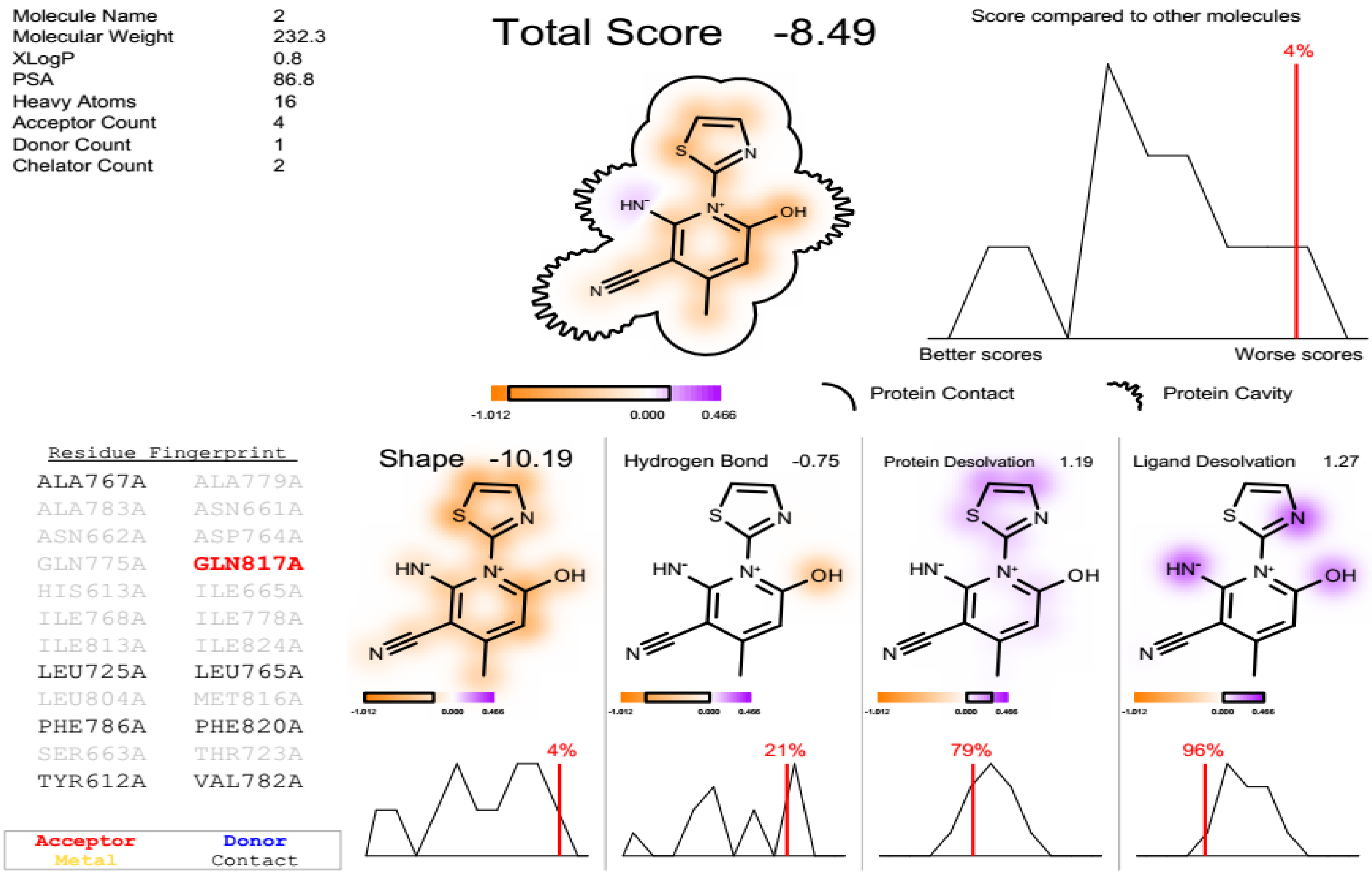

Figure S15. Molecular modeling study: Fred view of compound **2**.

Experimental

All melting points are uncorrected. IR spectra (KBr) were recorded on a FTIR 5300 spectrometer (ν, cm^{-1}). The (1D NMR); ^{1}H NMR, ^{13}C NMR, DEPT 135, NOE ^{13}C NMR and (2D NMR); HH COSY, CH COSY spectra were recorded in DMSO-d_6 and $CDCl_3$ at 400, 500 MHz on JEOL and Broker NMR spectrometer (δ, ppm) using TMS as an internal standard. Mass spectra were obtained on JEOL JMS600 H Root mass spectrometer at 70 ev. Elemental analysis was carried out by the Microanalytical Research Center, Faculty of Science, Cairo University and Microanalytical Research Center, Assiut University Broker Company in Switzerland Center.

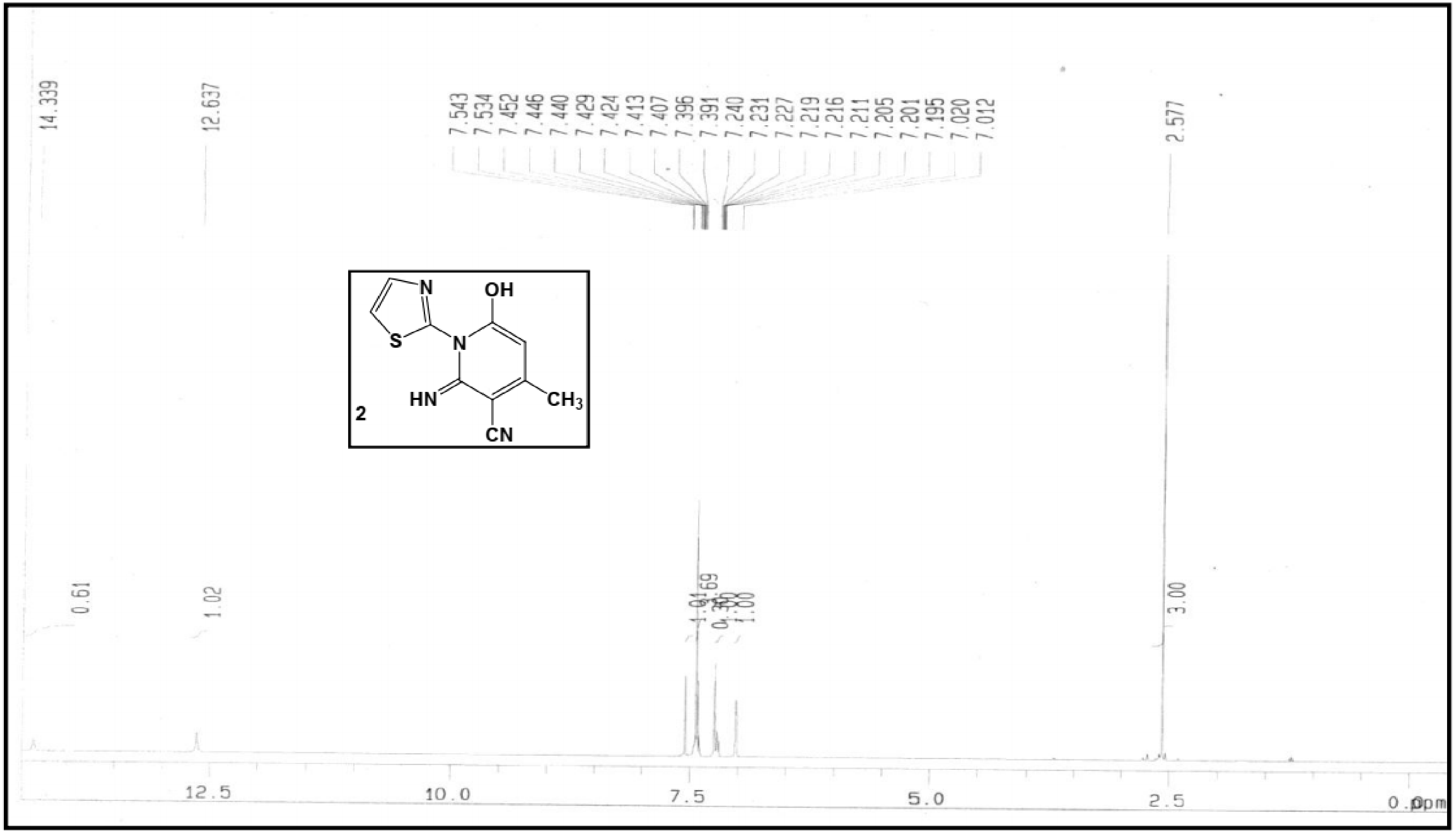

Figure S16. ^{1}H NMR spectrum of compound **2** ($CDCl_3$).

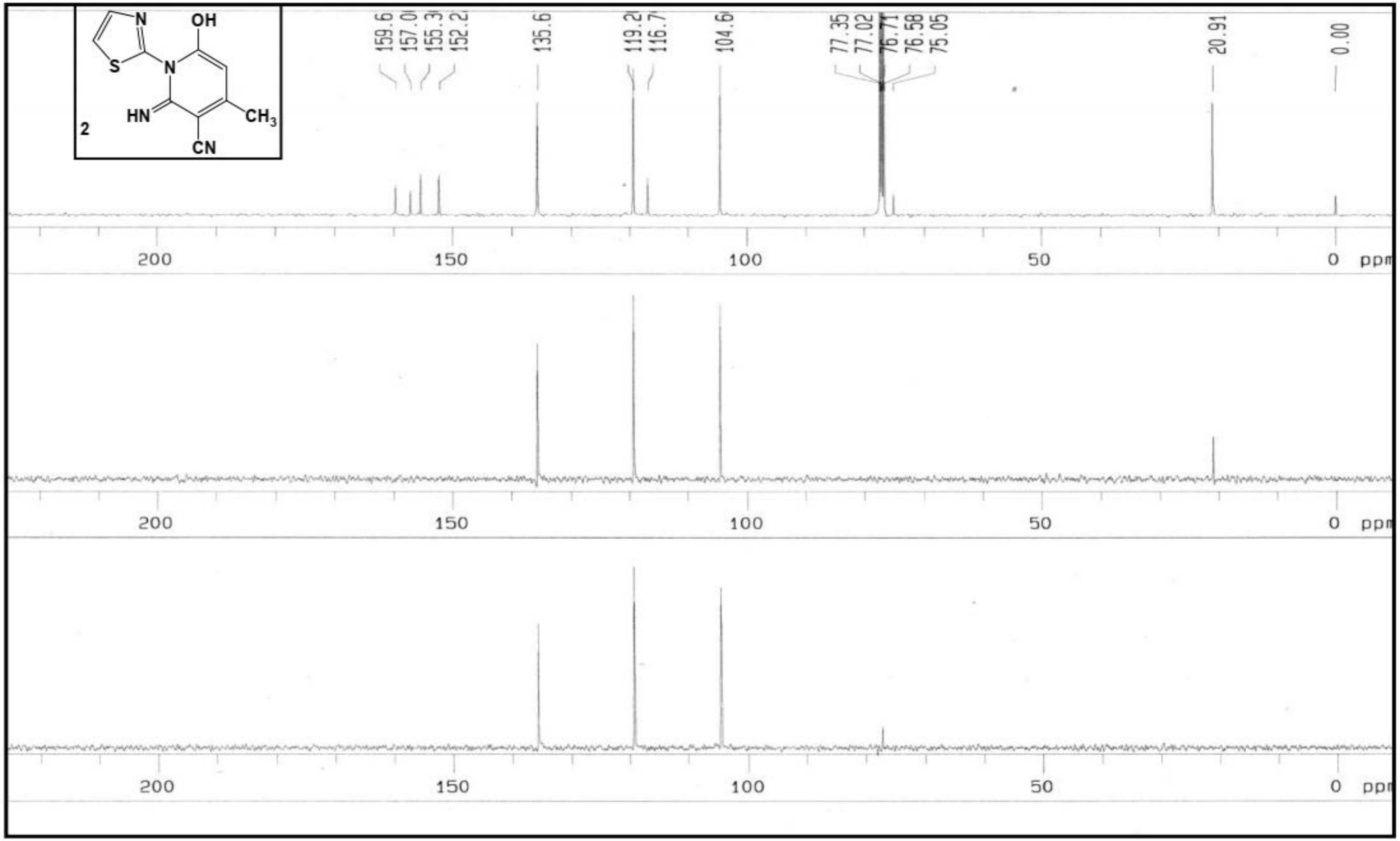

Figure S17. ^{13}C, DEPT-135, 90 NMR spectrum of compound **2** ($CDCl_3$).

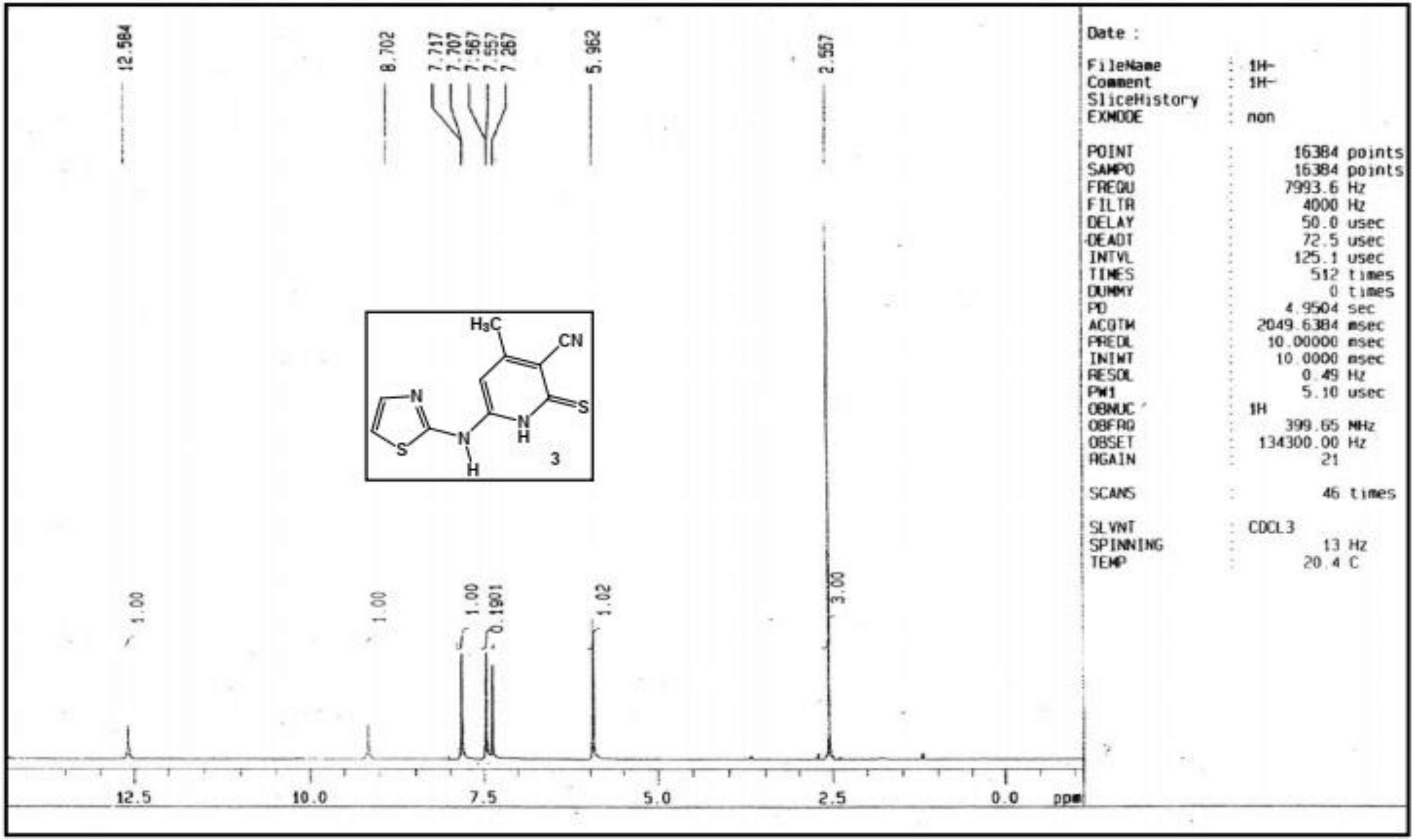

Figure S18. ^{1}H NMR spectrum of compound **3** ($CDCl_3$).

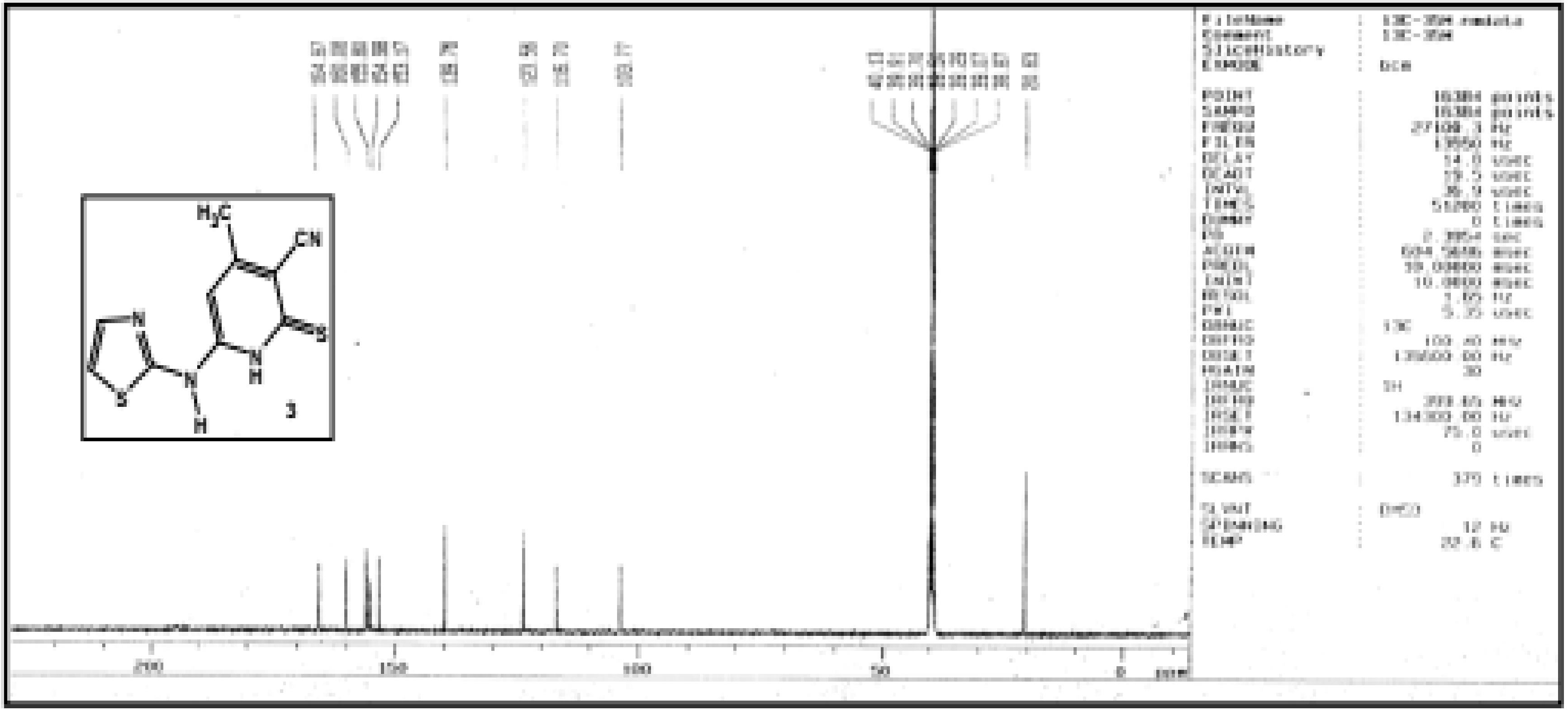

Figure S19. DEPT-135, 90 NMR spectrum of compound **3** ($CDCl_3$).

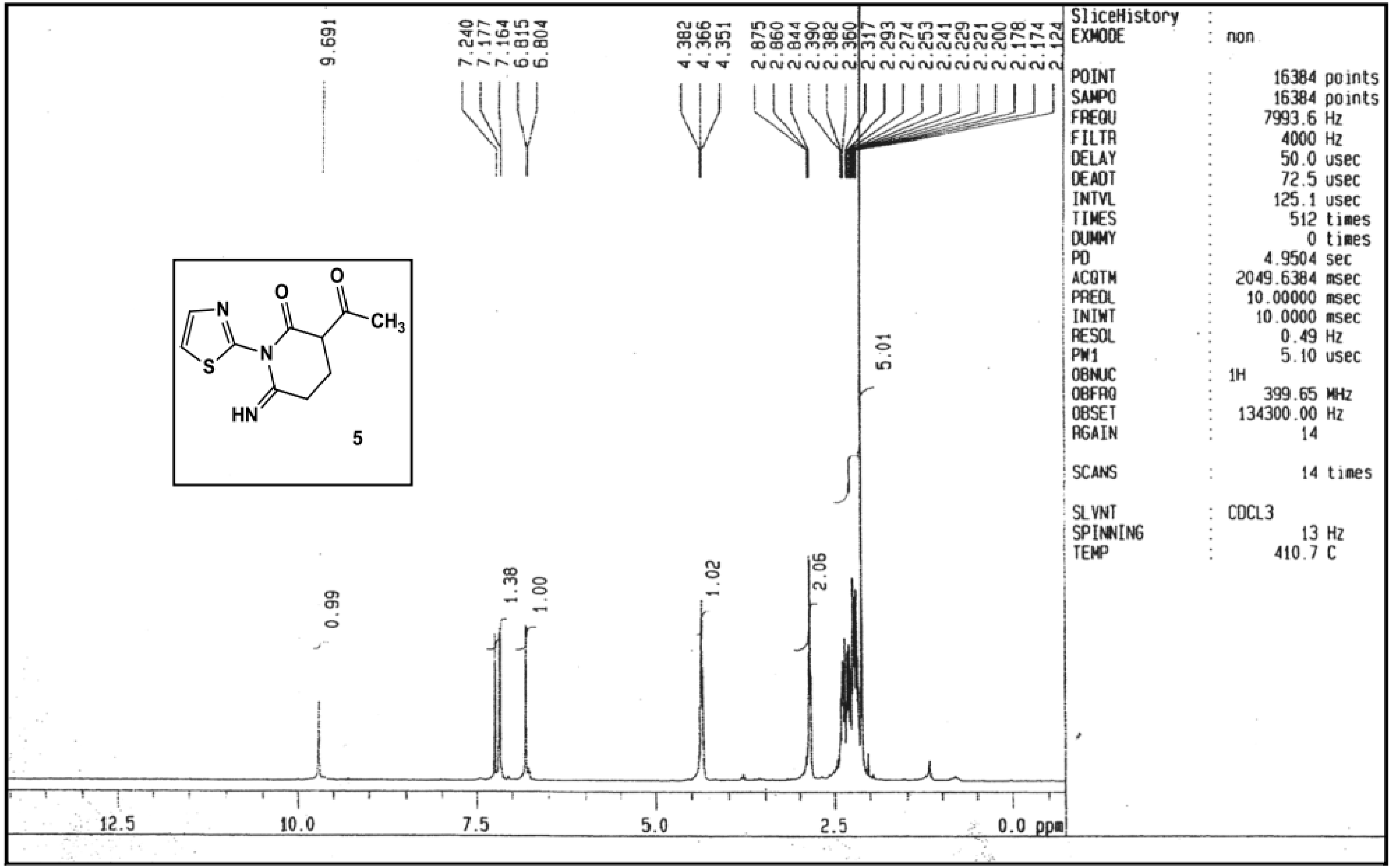

Figure S20. ^{1}H NMR spectrum of compound **5** ($CDCl_3$).

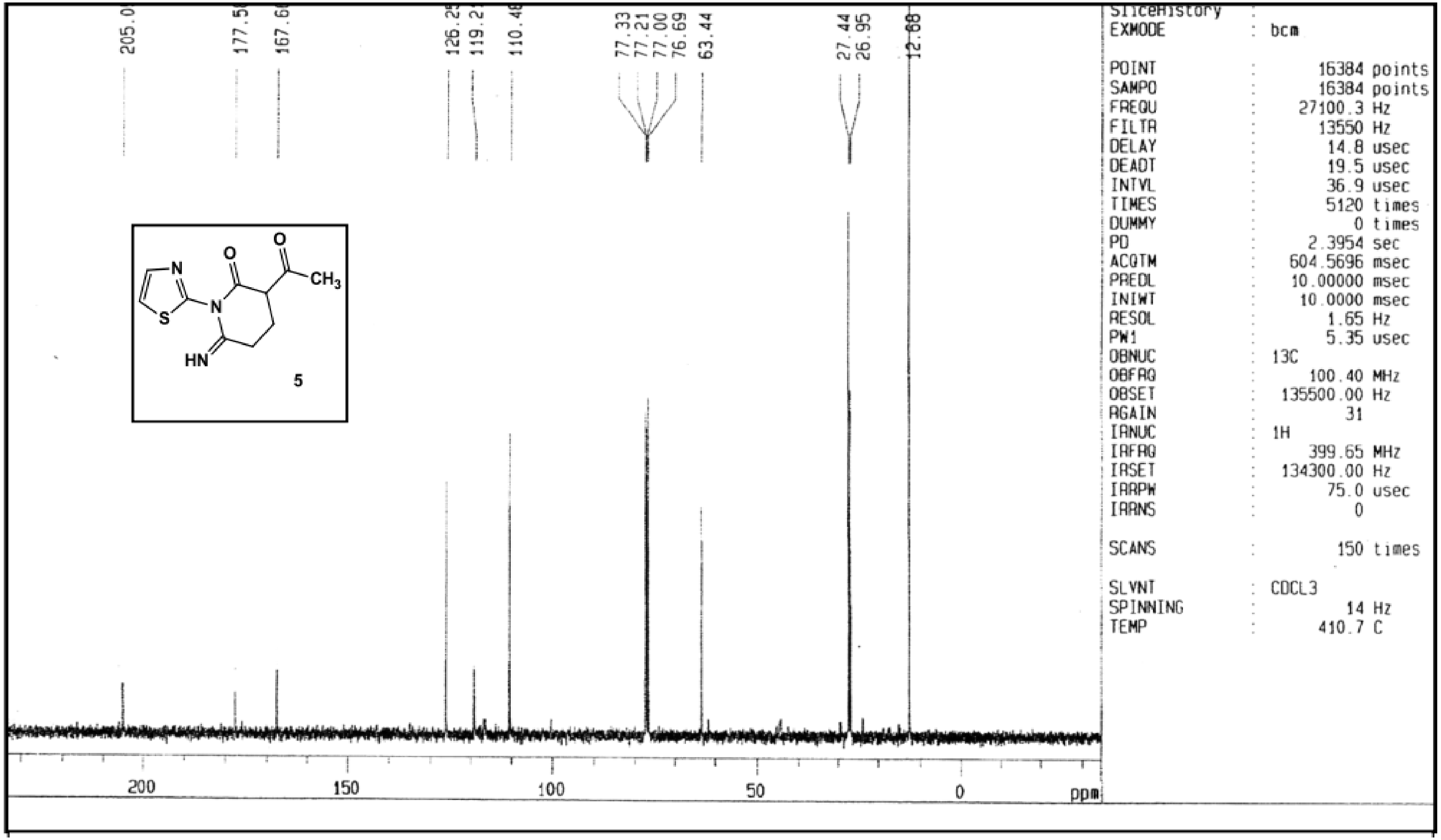

Figure S21. ^{13}C NMR spectrum of compound **5** ($CDCl_3$).

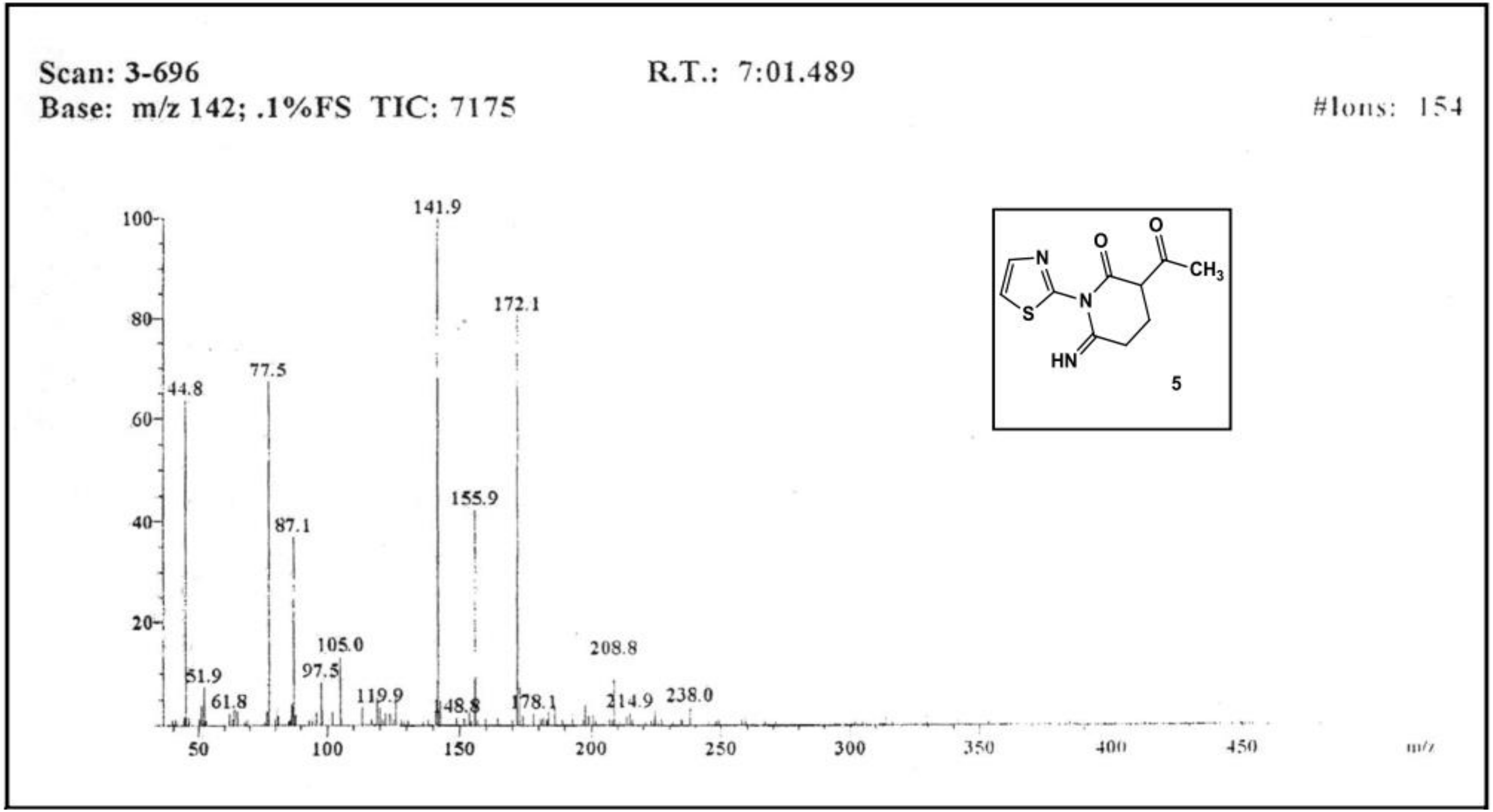

Figure S22. Mass spectrum of compound **5.**

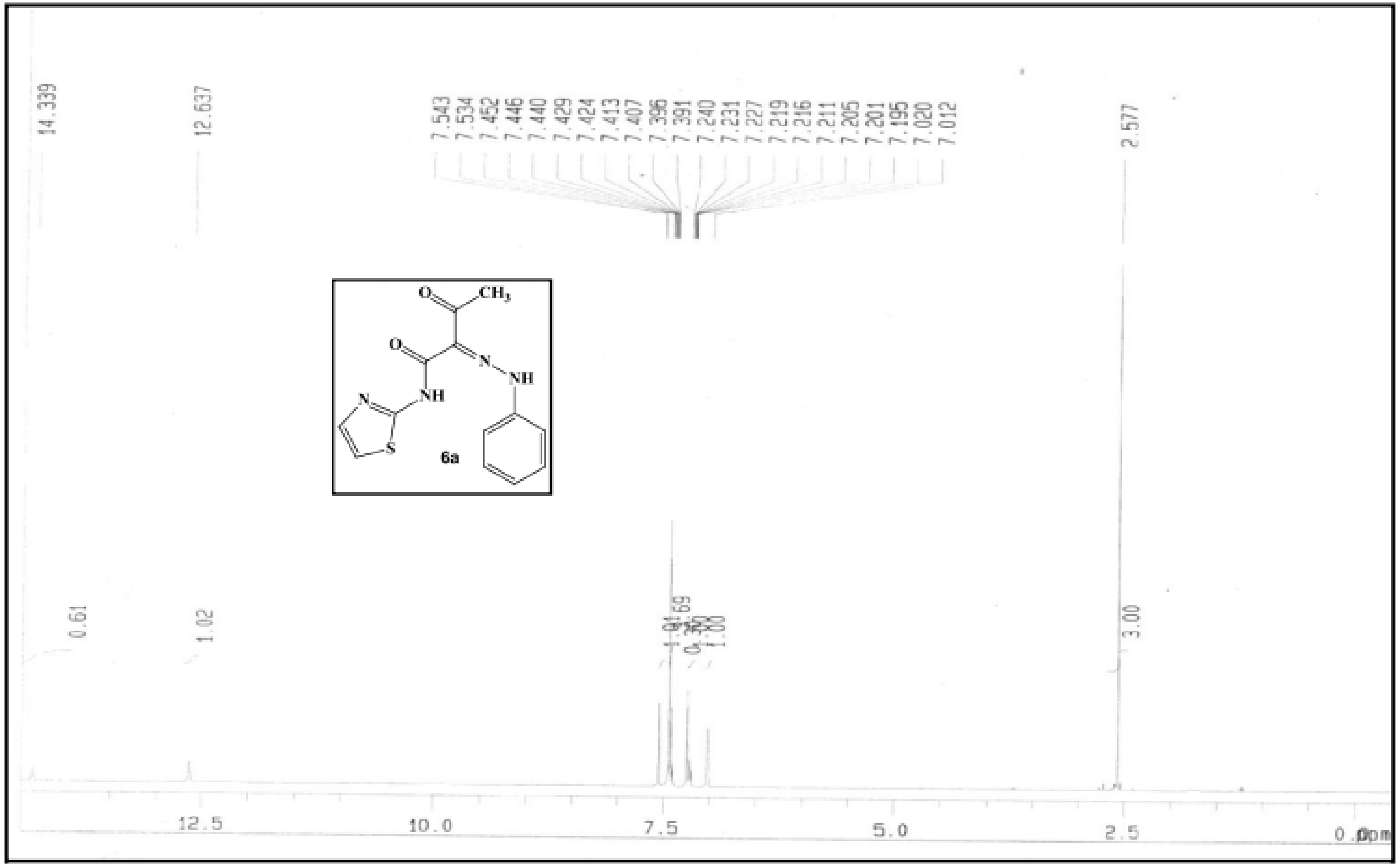

Figure S23. ^{1}H NMR spectrum of compound **6a** ($CDCl_3$).

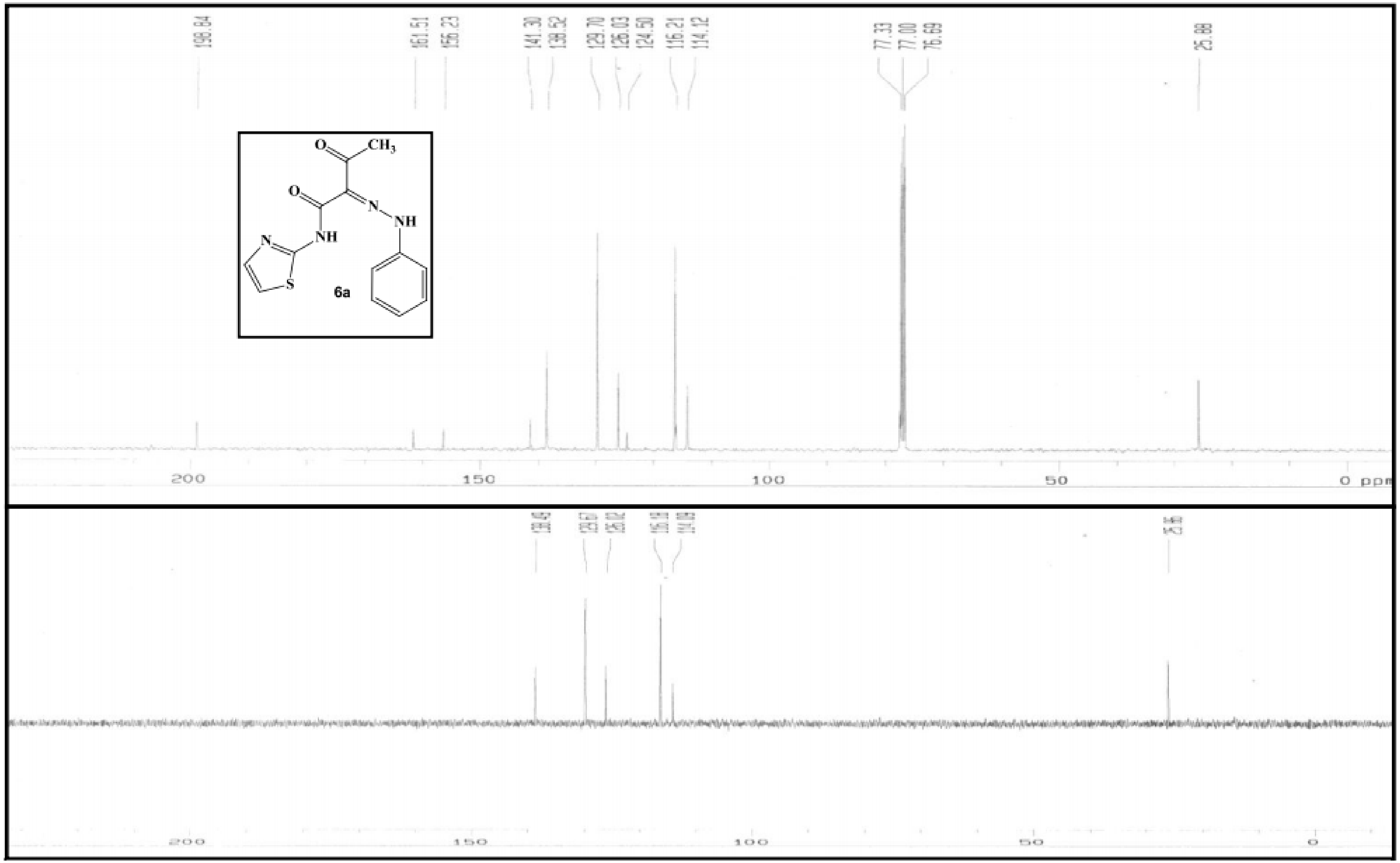

Figure S24. ^{13}C, DEPT-135 NMR spectrum of compound **6a** ($CDCl_3$).

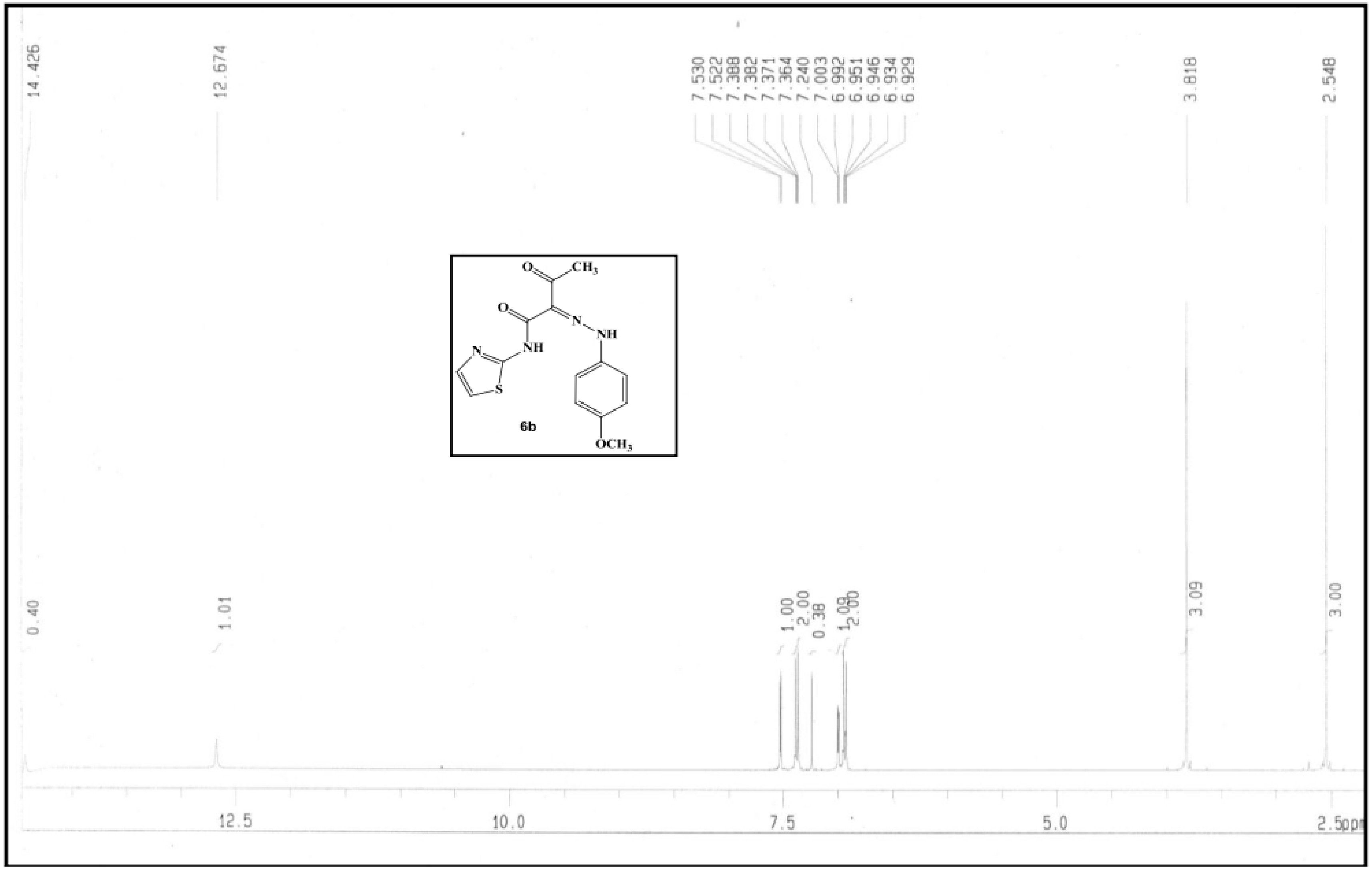

Figure S25. ^{1}H NMR spectrum of compound **6b** ($CDCl_3$).

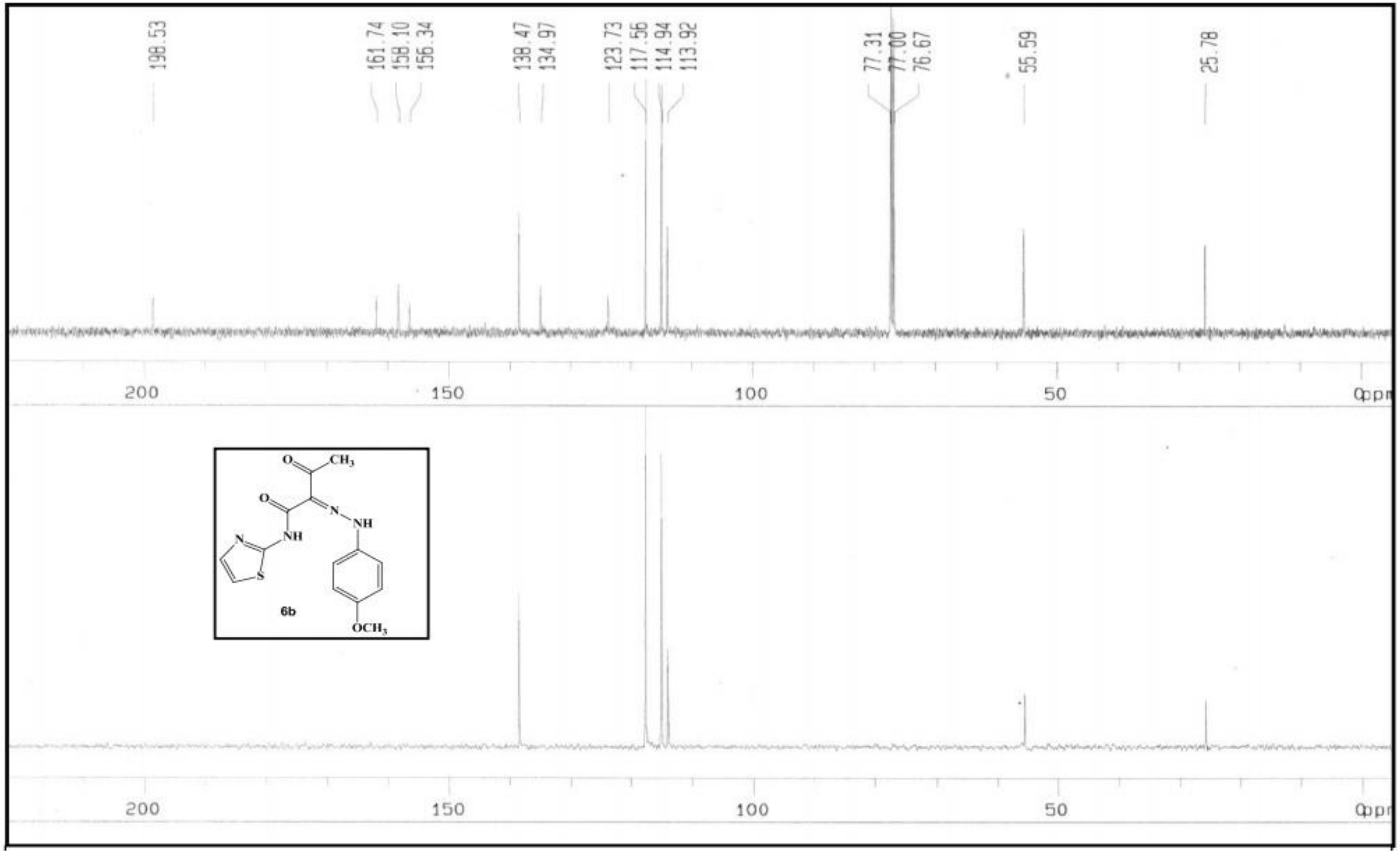

Figure S26. ^{13}C, DEPT-135 NMR spectrum of compound **6b** ($CDCl_3$).

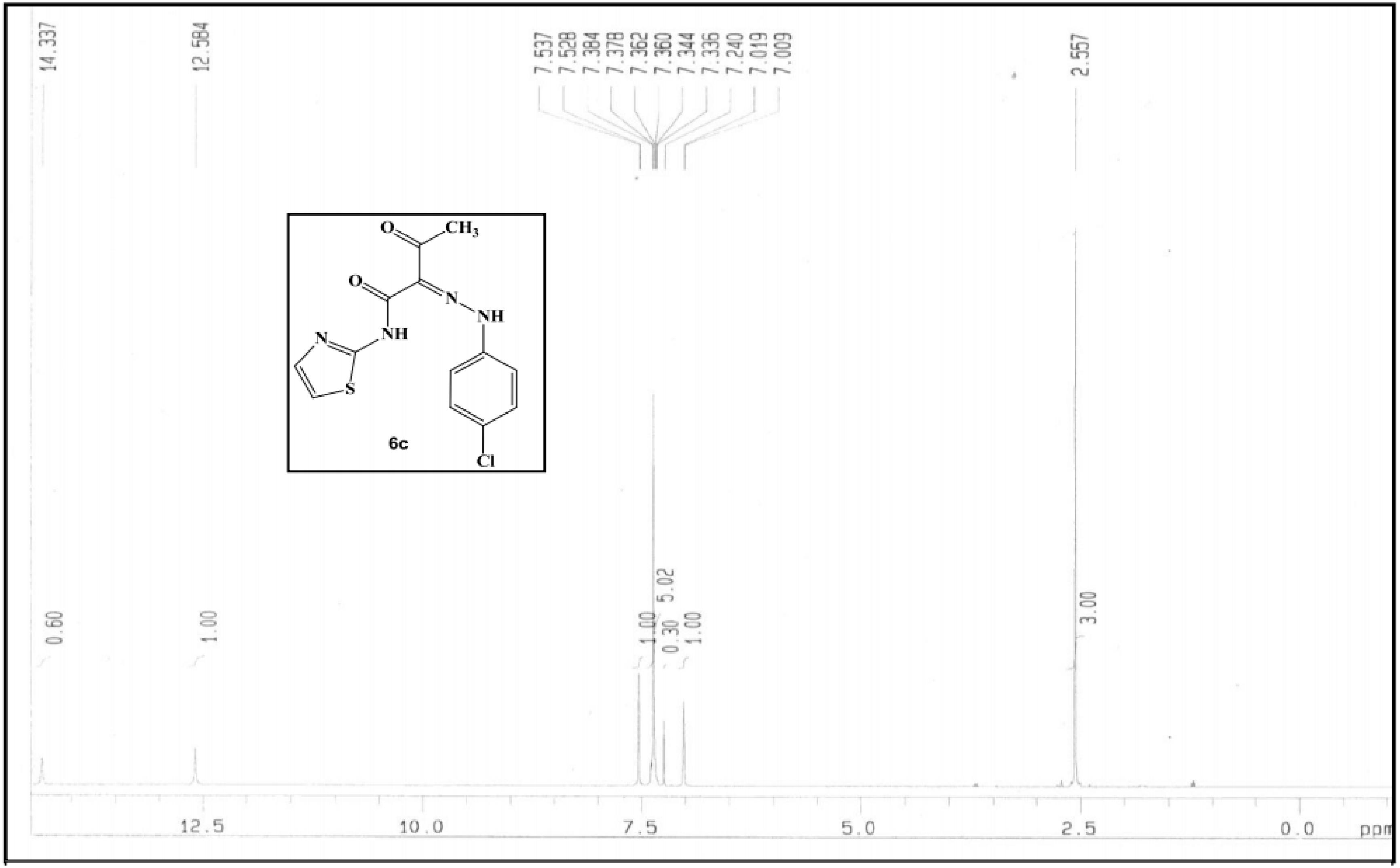

Figure S27. ^{1}H NMR spectrum of compound **6c** ($CDCl_3$).

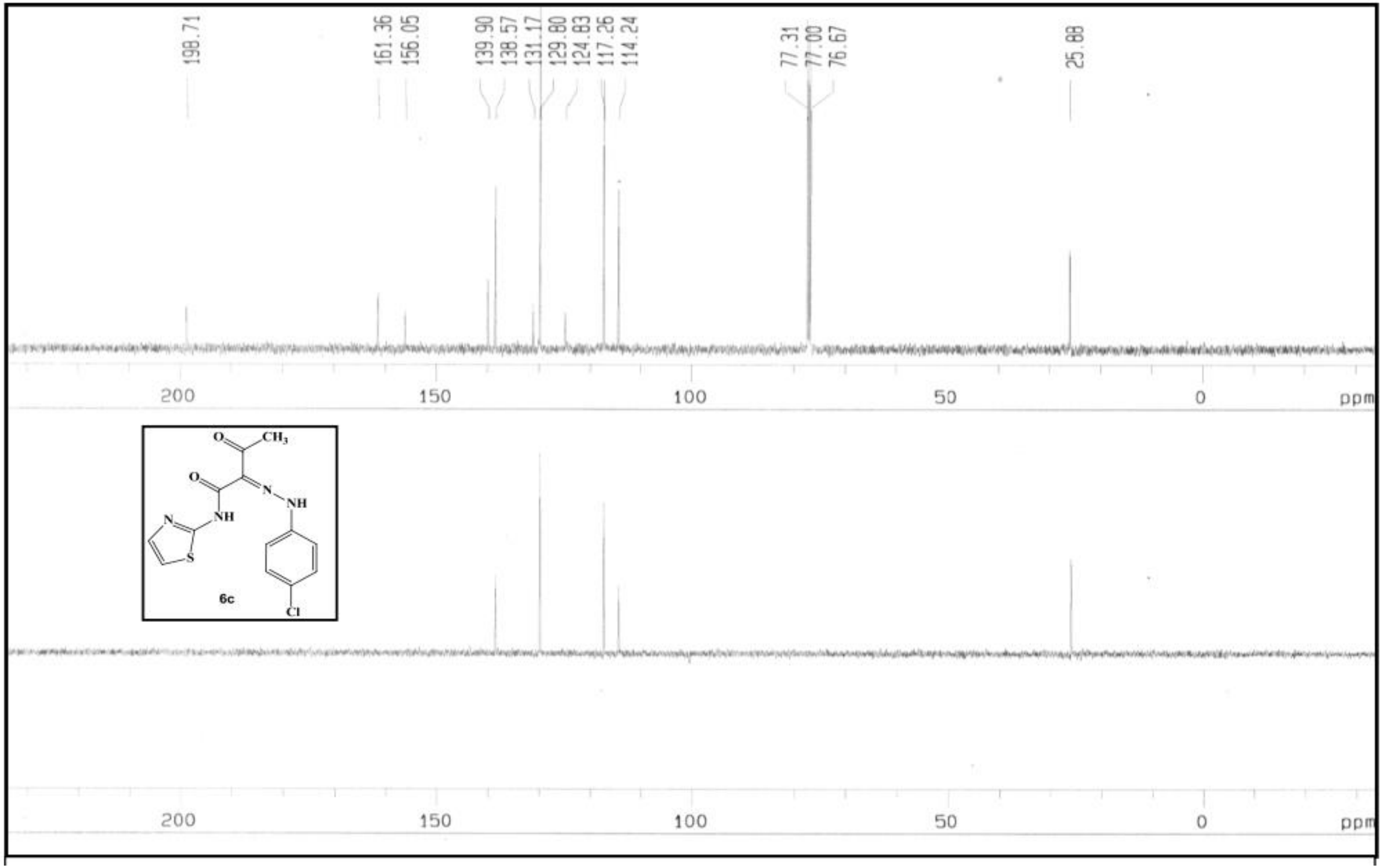

Figure S28. ^{13}C, DEPT-135 NMR spectrum of compound **6c** ($CDCl_3$).

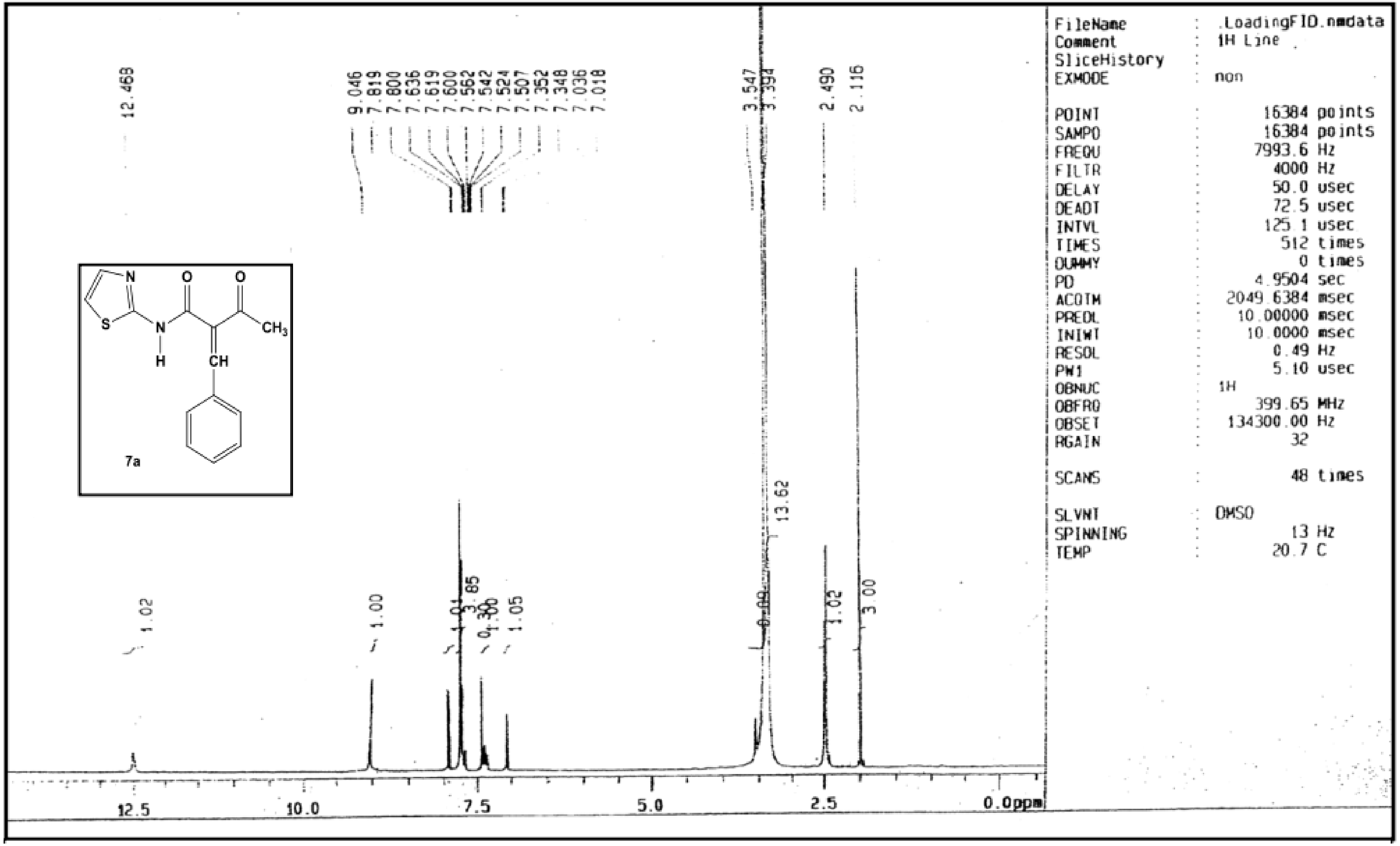

Figure S29. ^{1}H NMR spectrum of compound **7a** (DMSO-d_6).

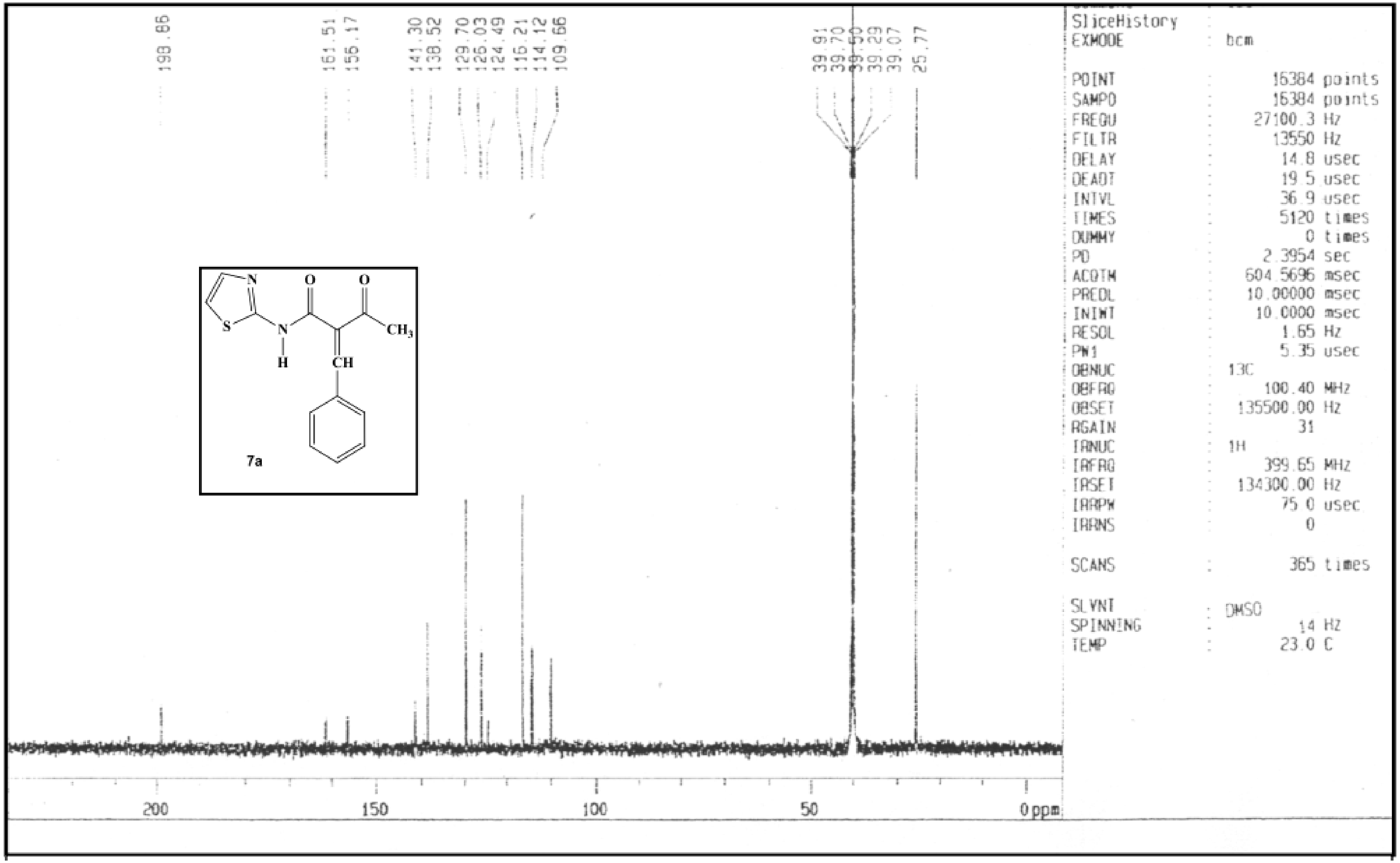

Figure S30. ^{13}C NMR spectrum of compound **7a** (DMSO-d_6).

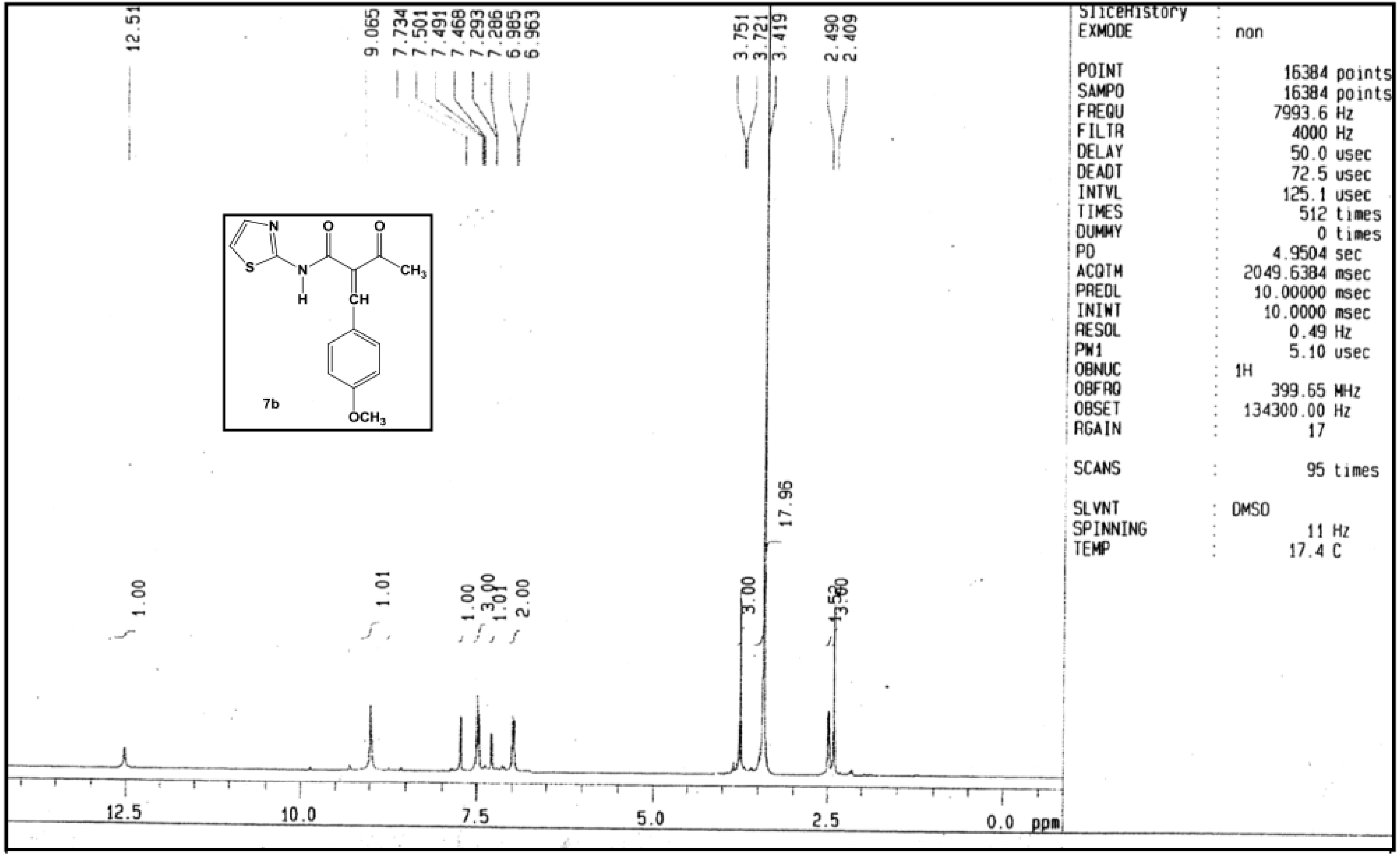

Figure S31. ^{1}H NMR spectrum of compound **7b** (DMSO-d_6).

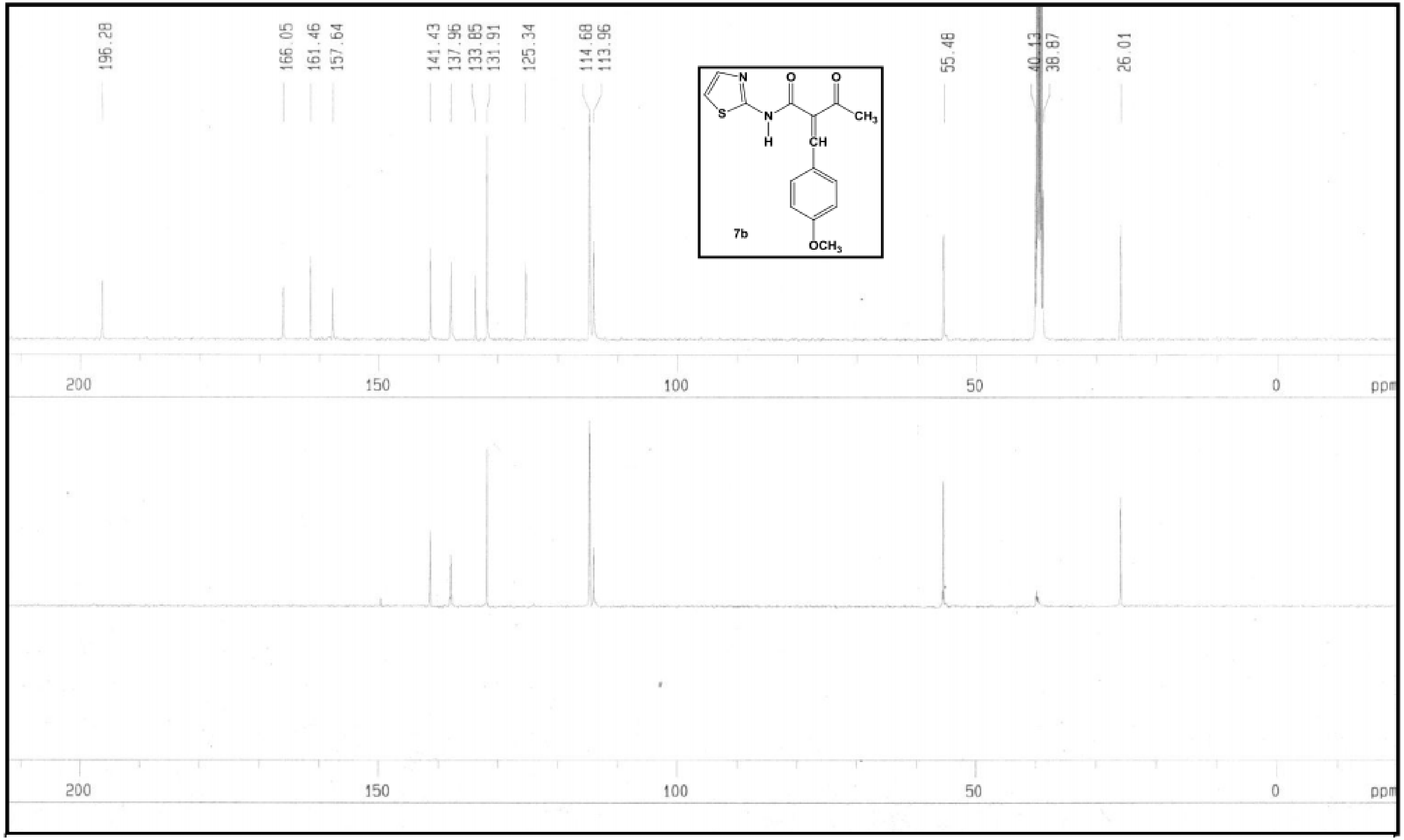

Figure S32. ^{13}C, DEPT-135 NMR spectrum of compound **7b** (DMSO-d_6).

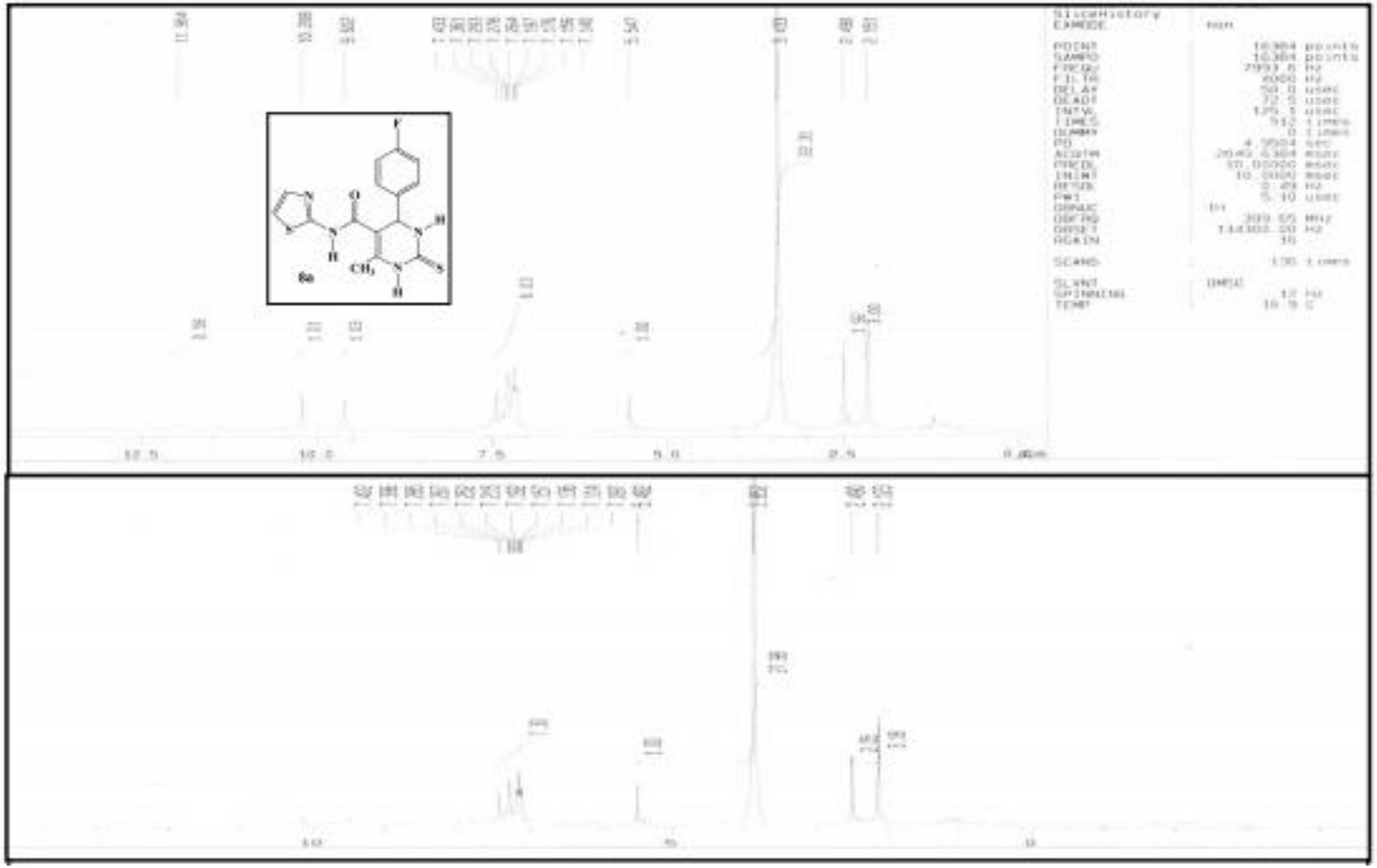

Figure S33. ^{1}H, $^{1}H+D_2O$ NMR spectrum of compound **8a** (DMSO-d_6).

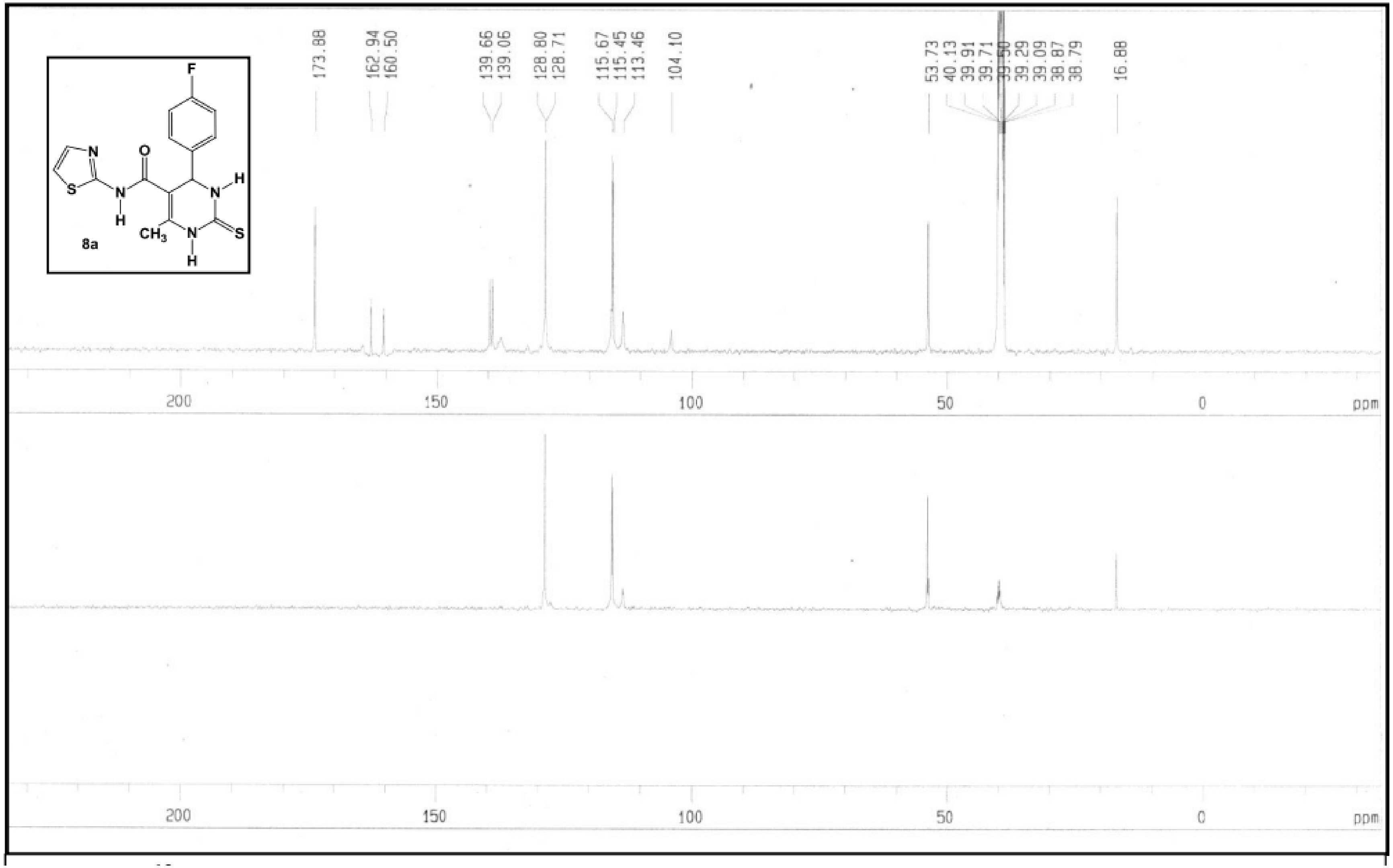

Figure S34. ^{13}C, DEPT-135 NMR spectrum of compound **8a** (DMSO-d_6).

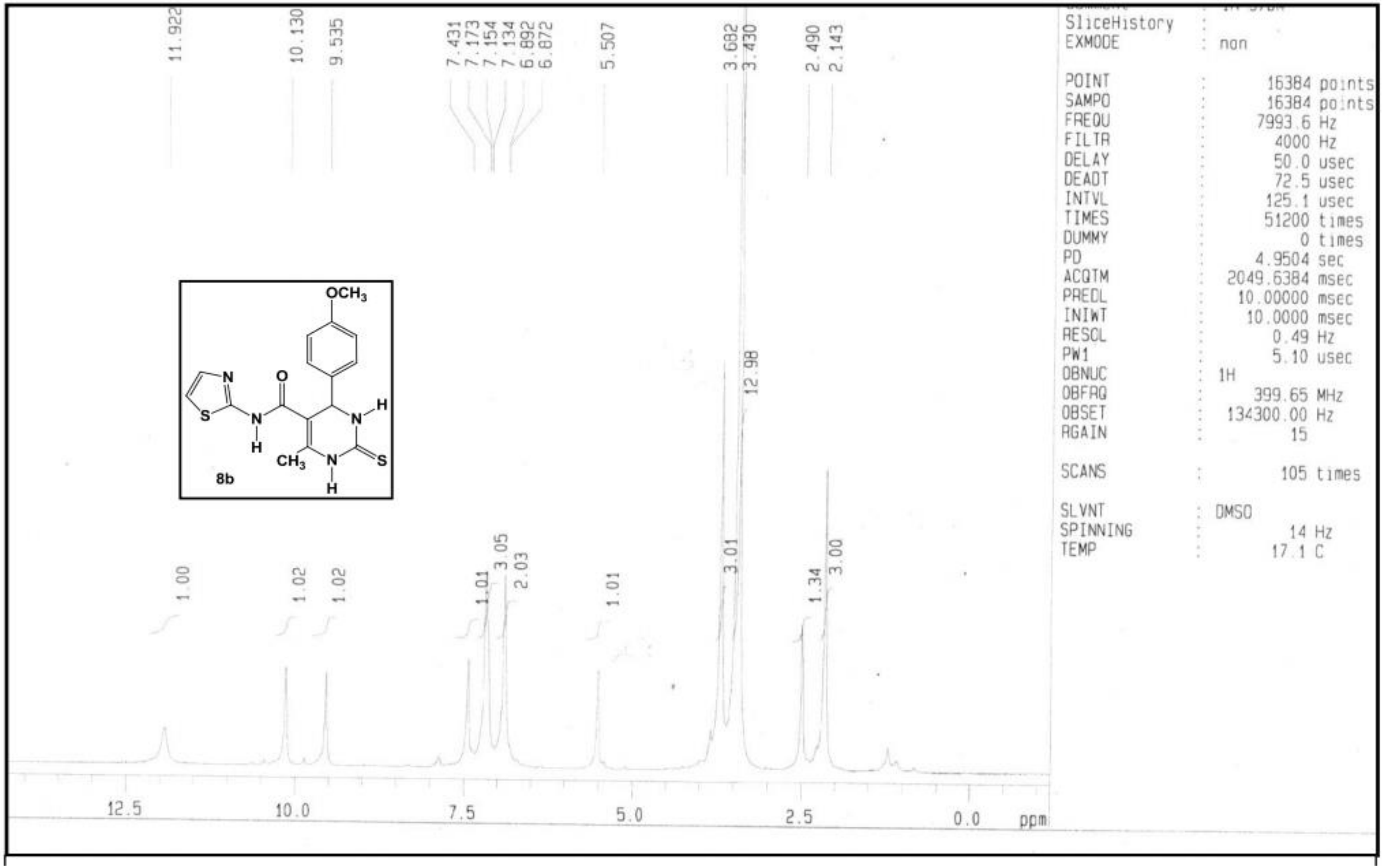

Figure S35. ^{1}H NMR spectrum of compound **8b** (DMSO-d_6).

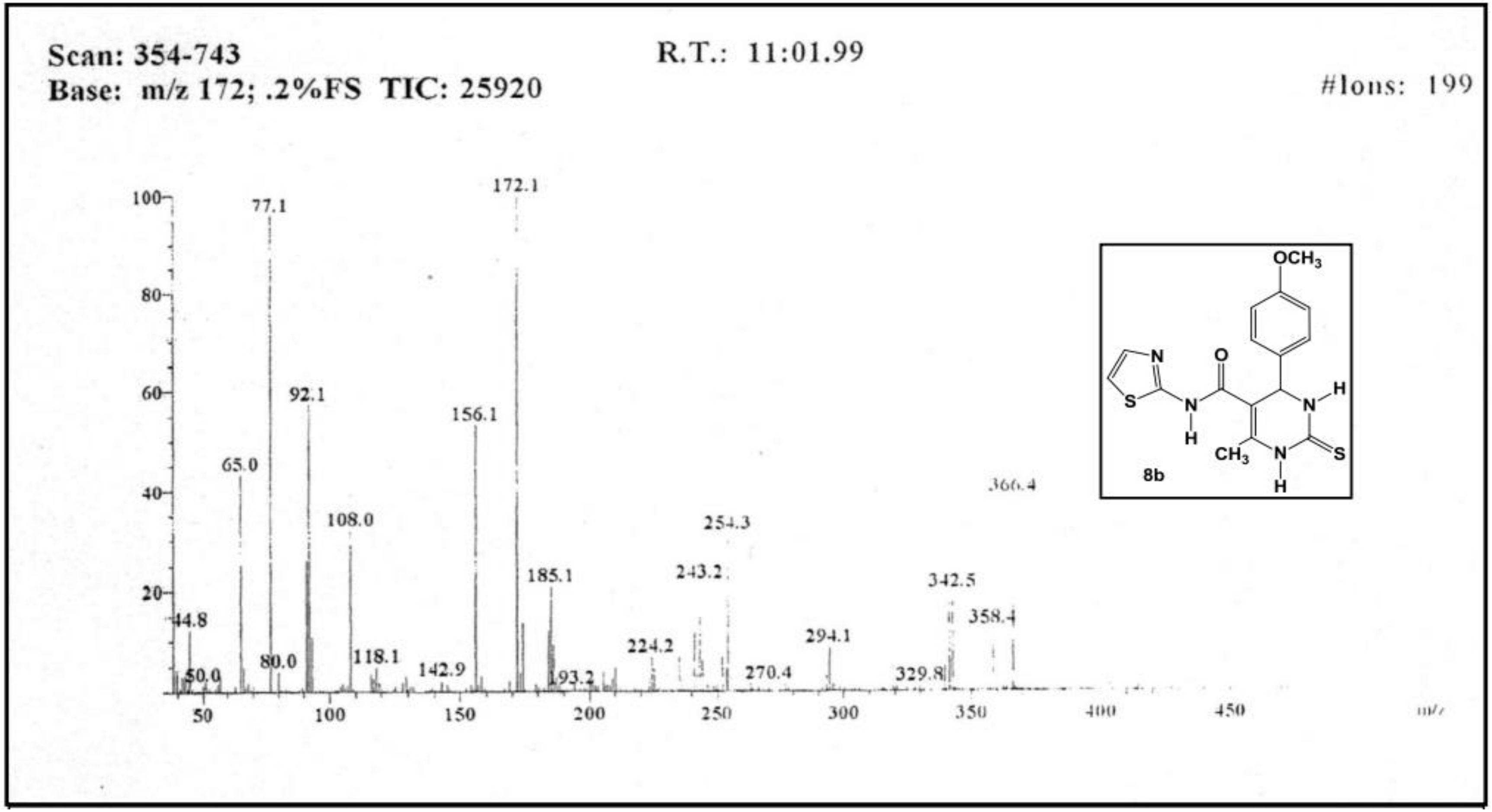

Figure S36. MS spectrum of compound **8b.**

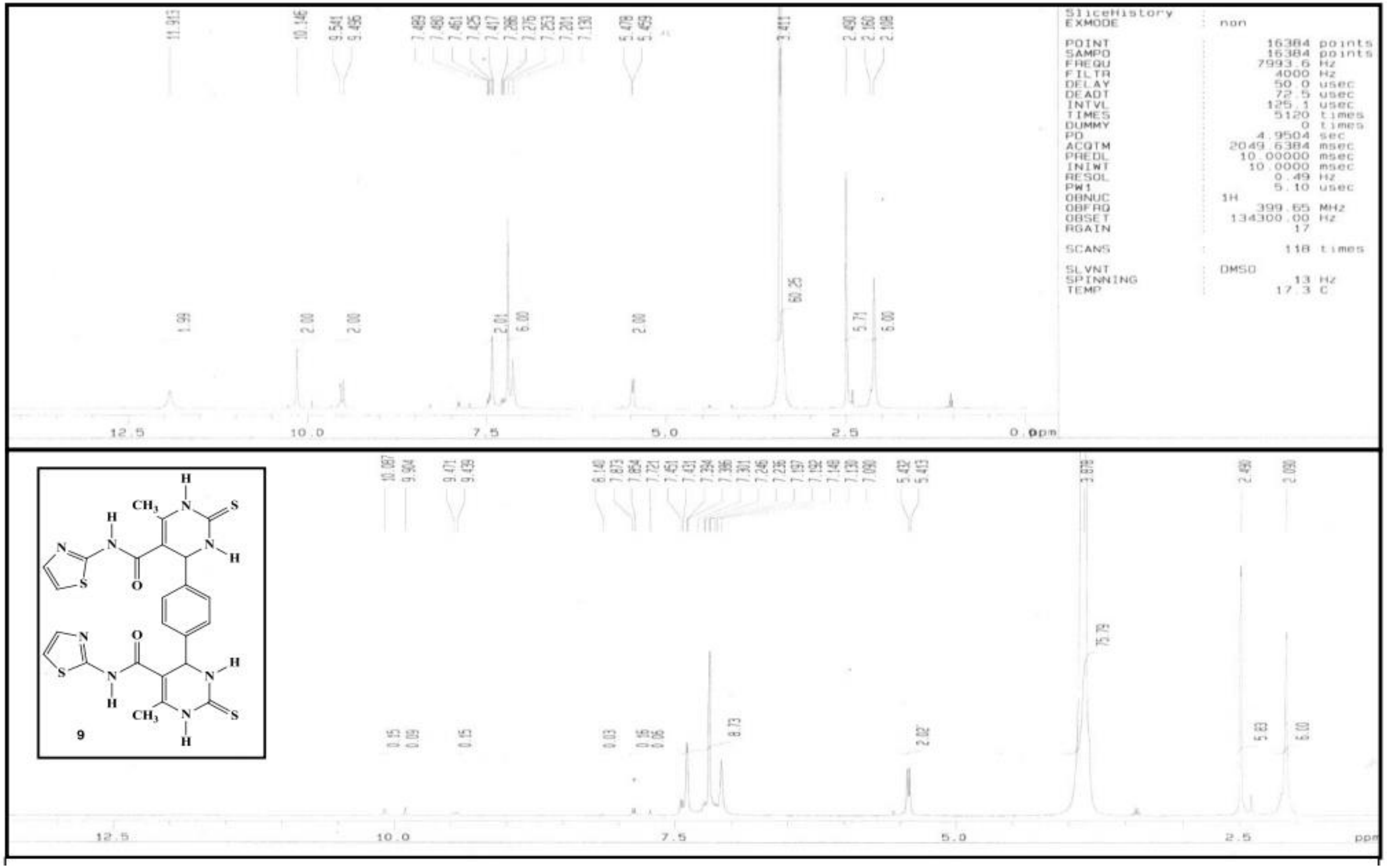

Figure S37. ^{1}H, $^{1}H+D_2O$ NMR spectrum of compound **9** (DMSO-d_6).

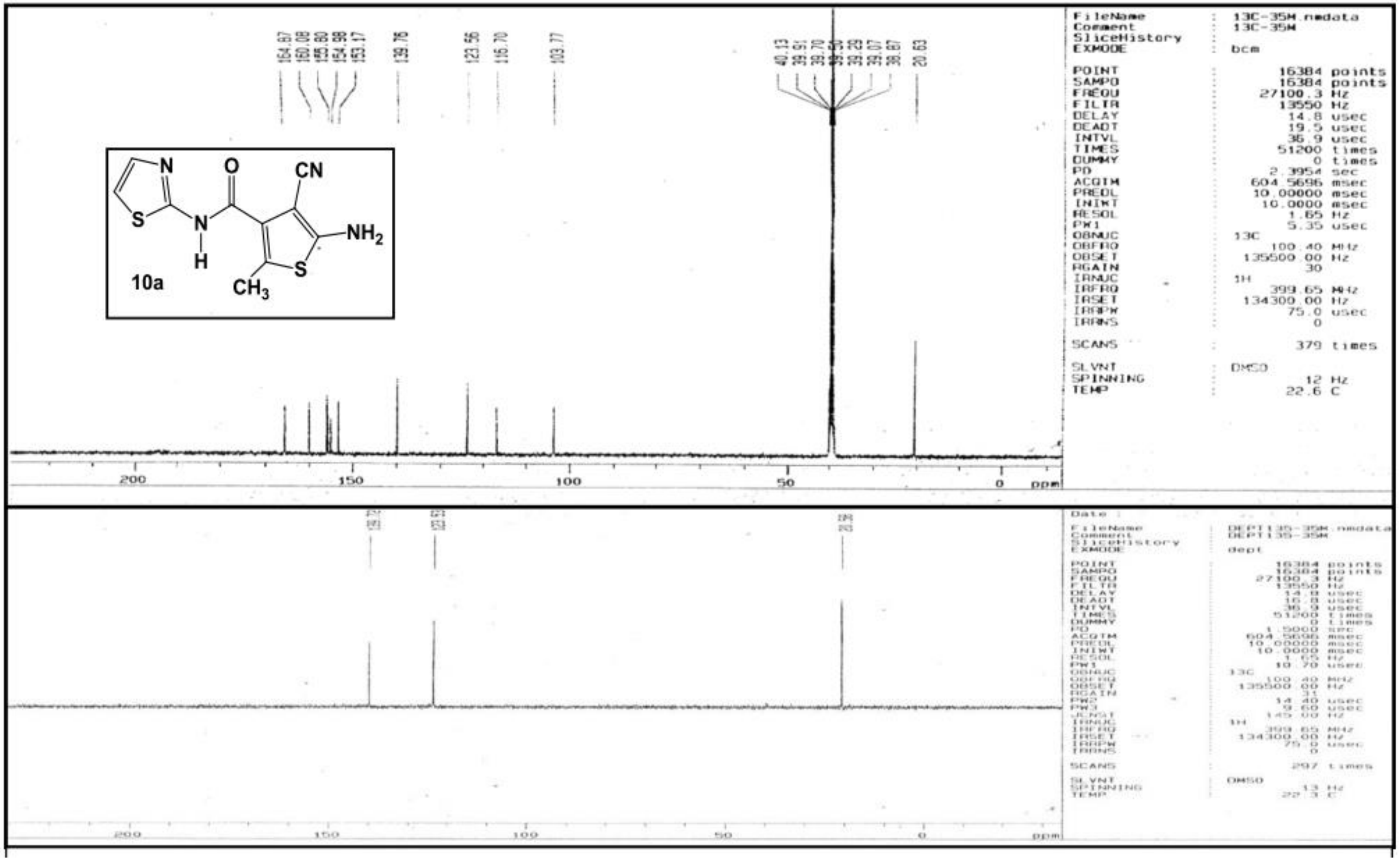

Figure S38. ^{13}C, DEPT-135 NMR spectrum of compound **10a** (DMSO-d_6).

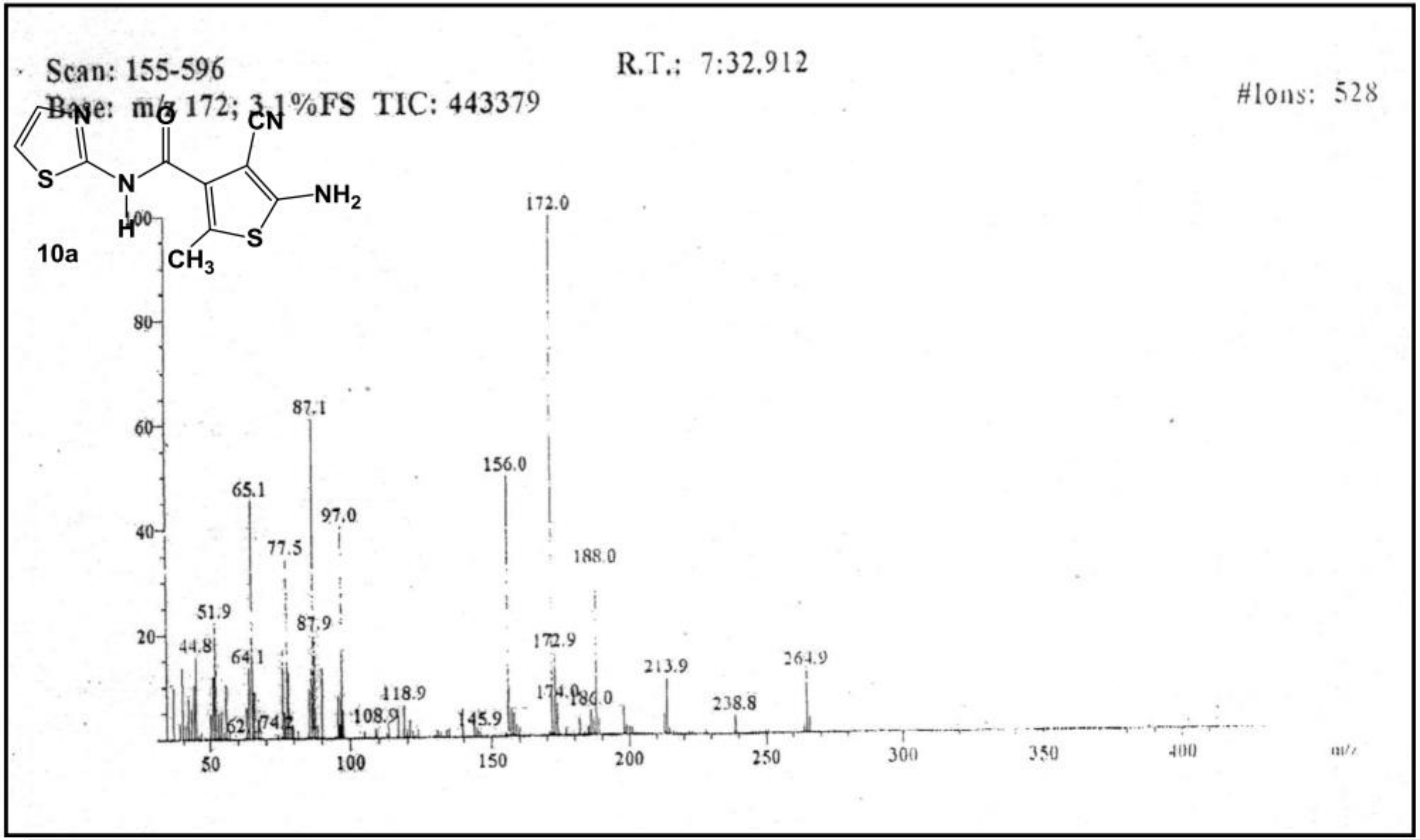

Figure S39. MS spectrum of compound **10a.**

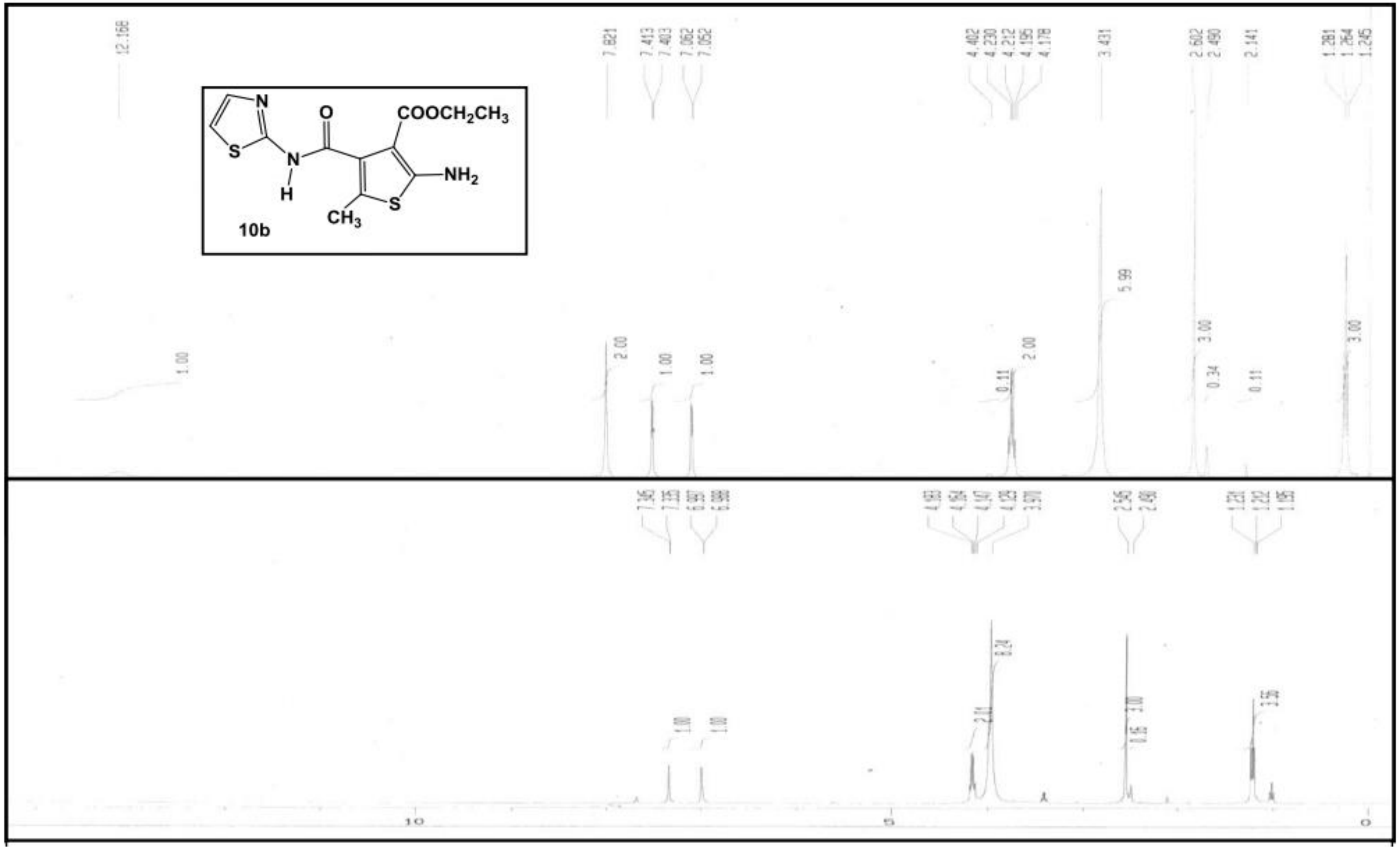

Figure S40. ^1H, $^1H+D_2O$ NMR spectrum of compound **10b** (DMSO-d_6).

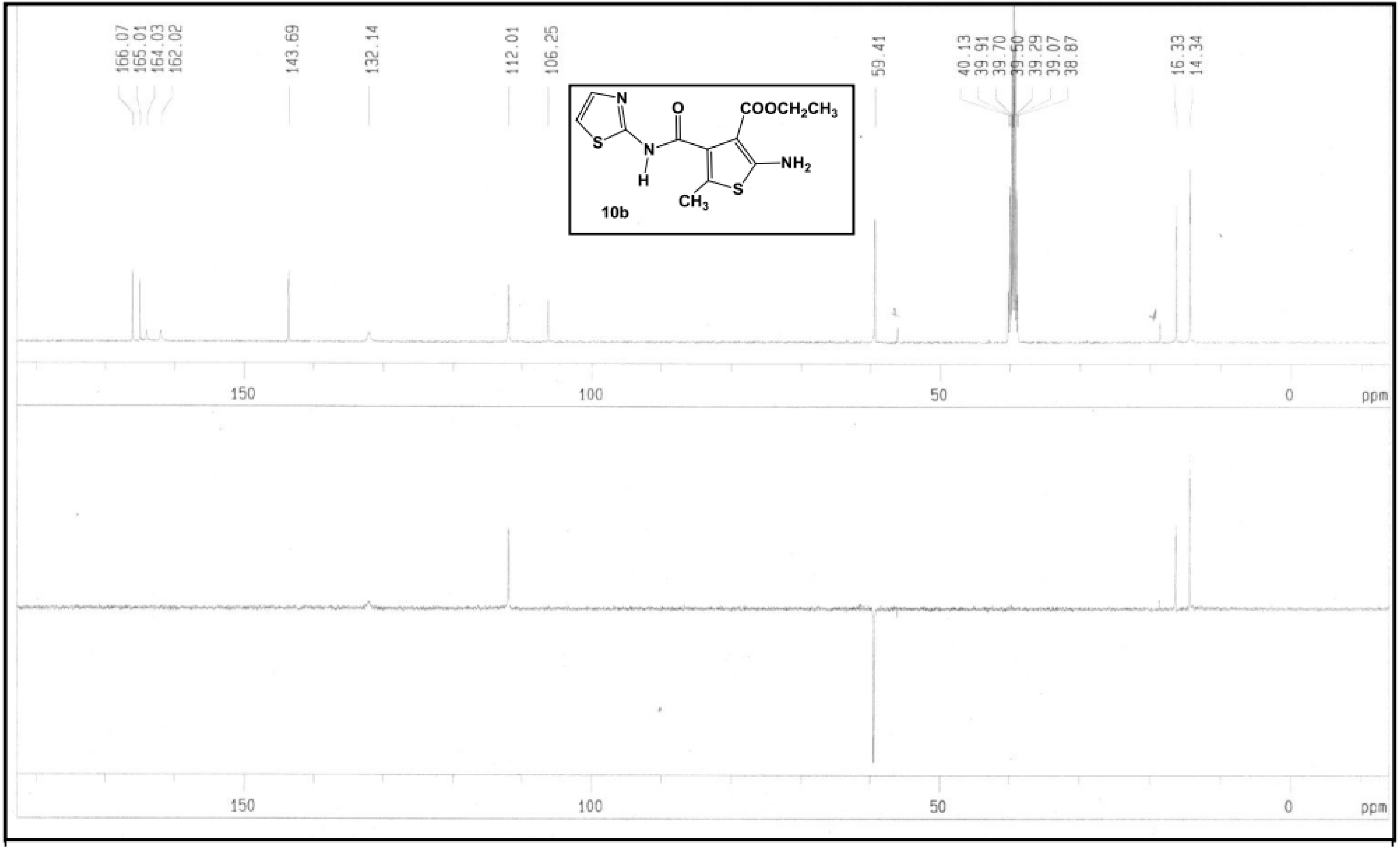

Figure S41. ^{13}C, DEPT-135 NMR spectrum of compound **10b** (DMSO-d_6).

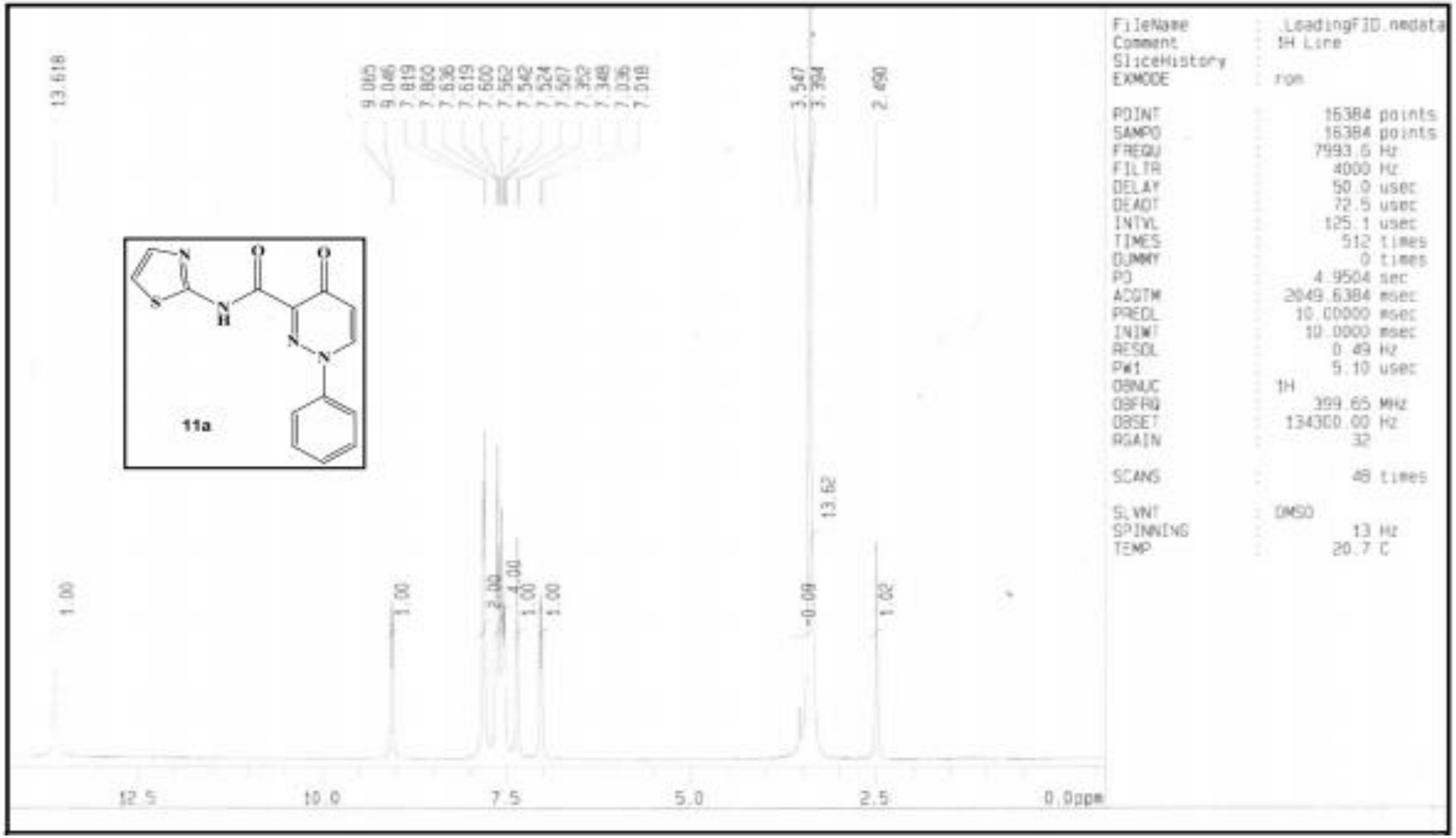

Figure S42. ^{1}H NMR spectrum of compound **11a** (DMSO-d_6).

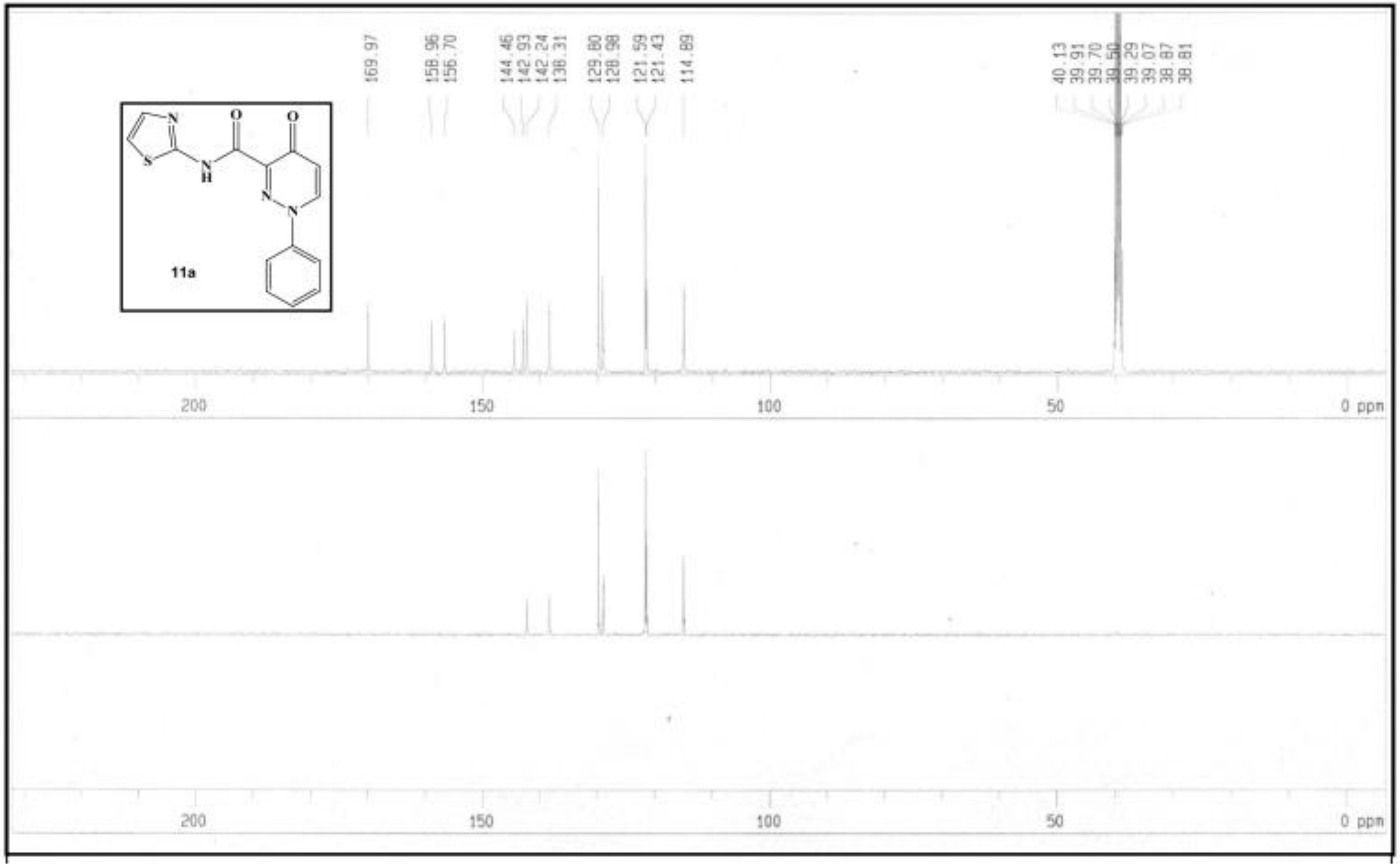

Figure S43. ^{13}C, DEPT-135 NMR spectrum of compound **11a** (DMSO-d_6).

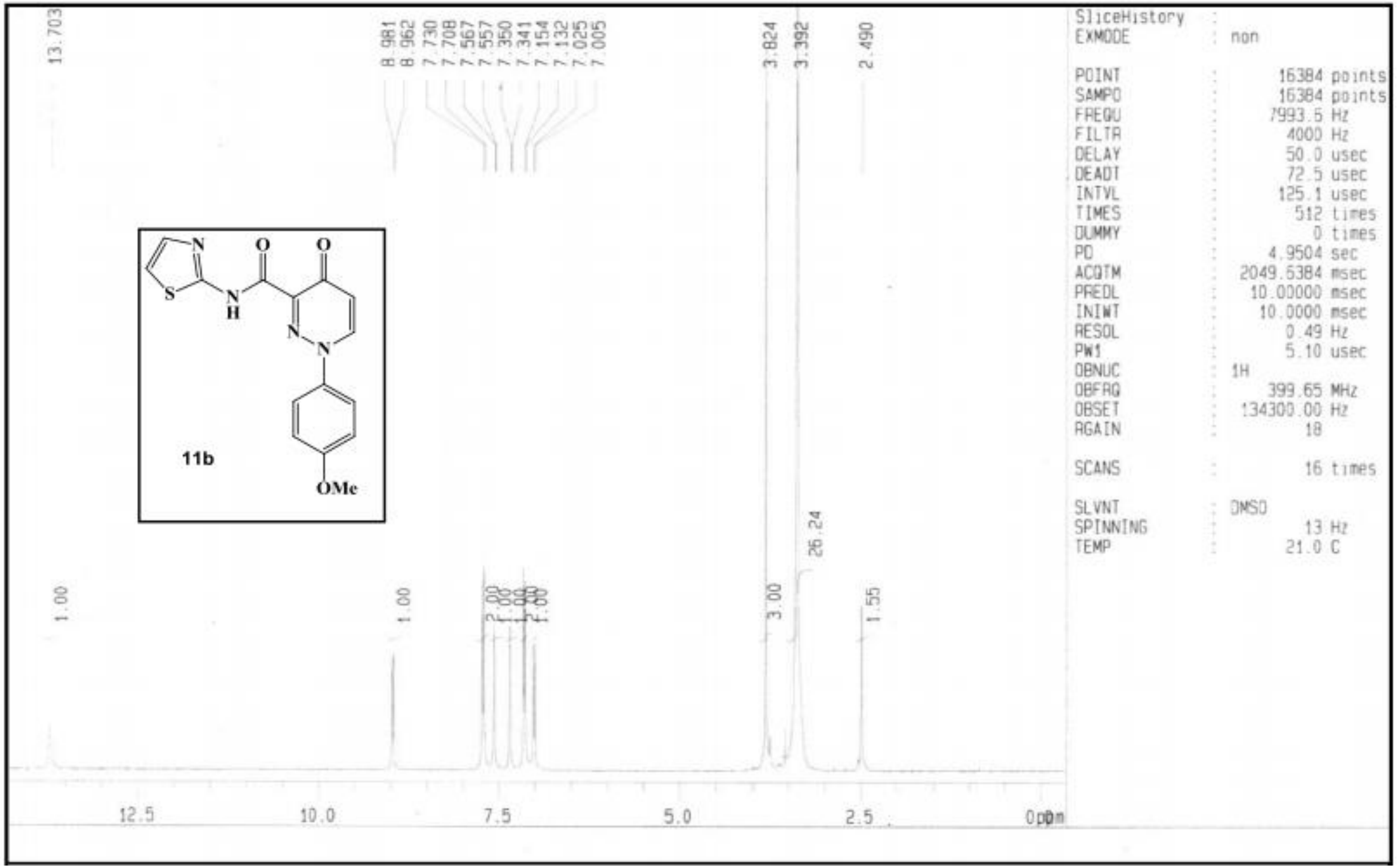

Figure S44. ^{1}H NMR spectrum of compound **11b** (DMSO-d_6).

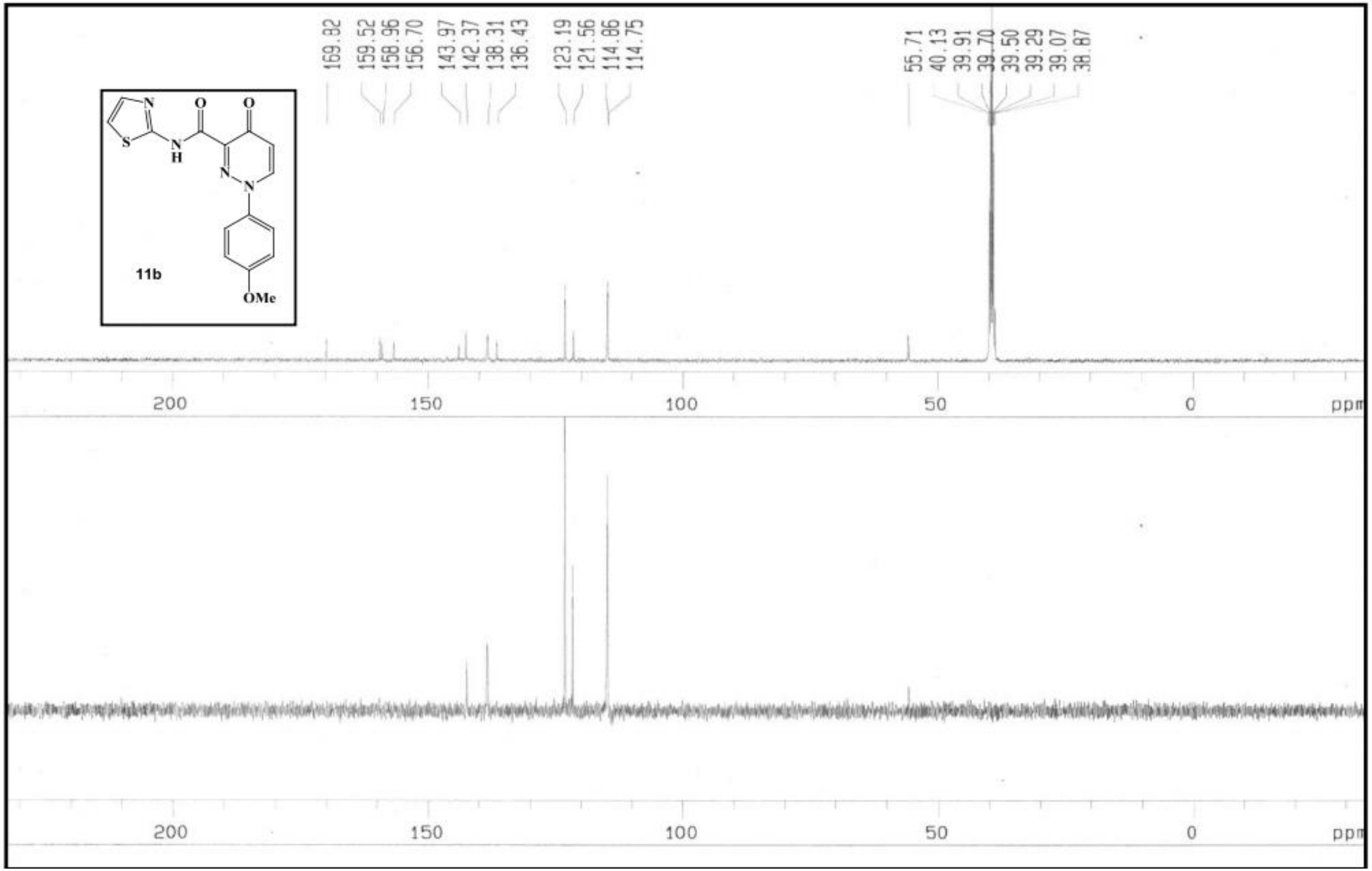

Figure S45. ^{13}C, DEPT-135 NMR spectrum of compound **11b** (DMSO-d_6).

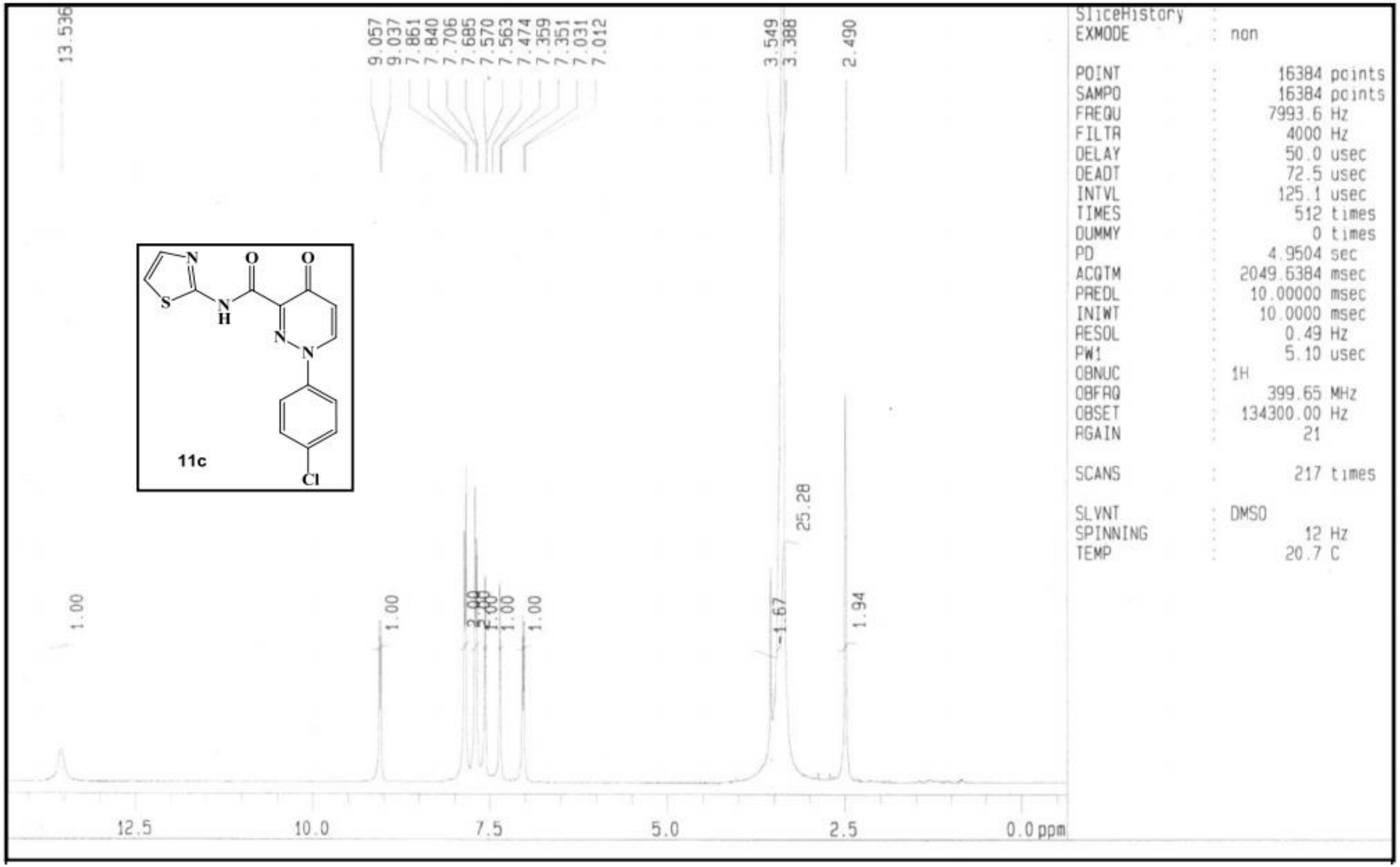

Figure S46. ^{1}H NMR spectrum of compound **11c** (DMSO-d_6).

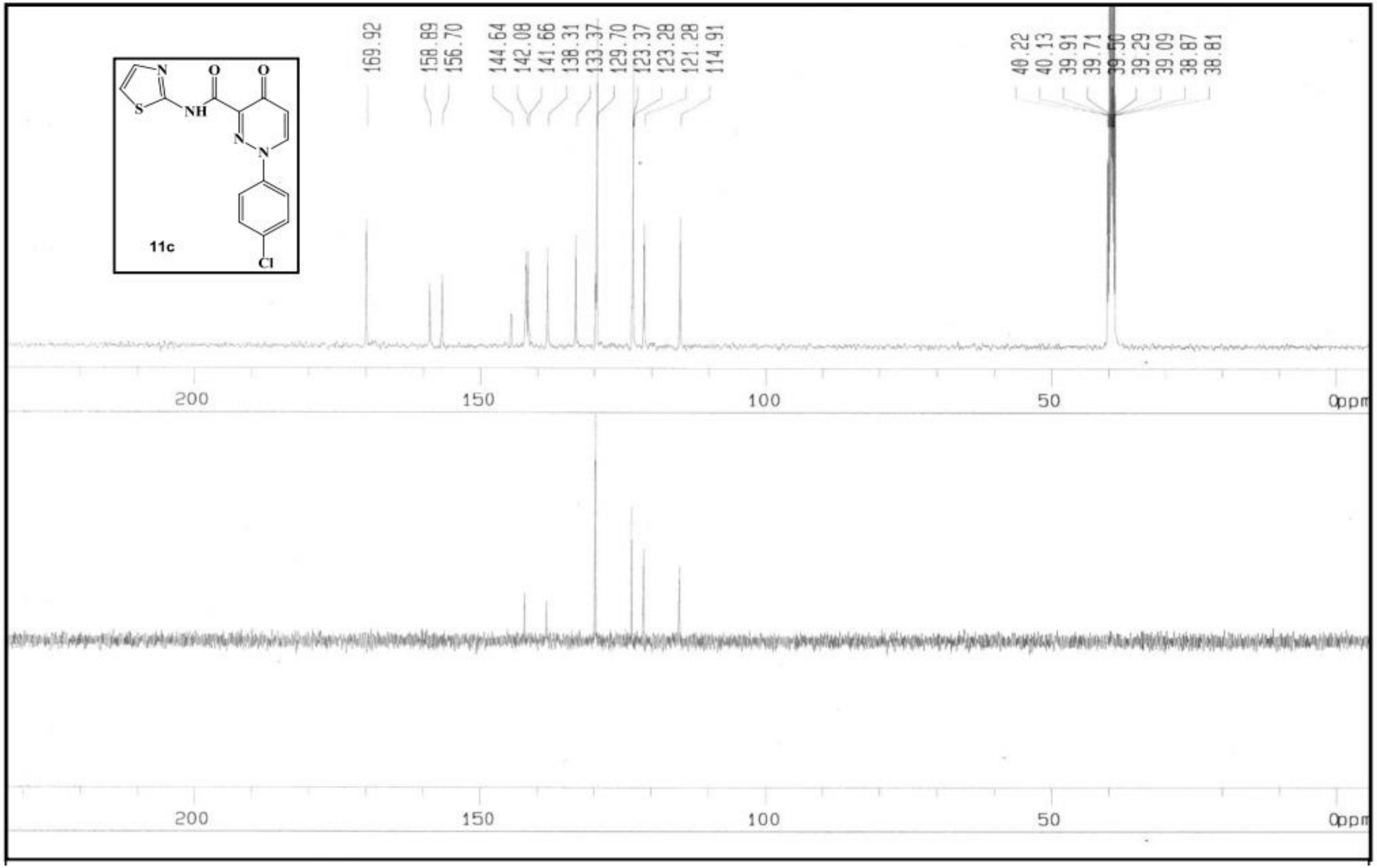

Figure S47. ^{13}C, DEPT-135 NMR spectrum of compound **11c** (DMSO-d_6).

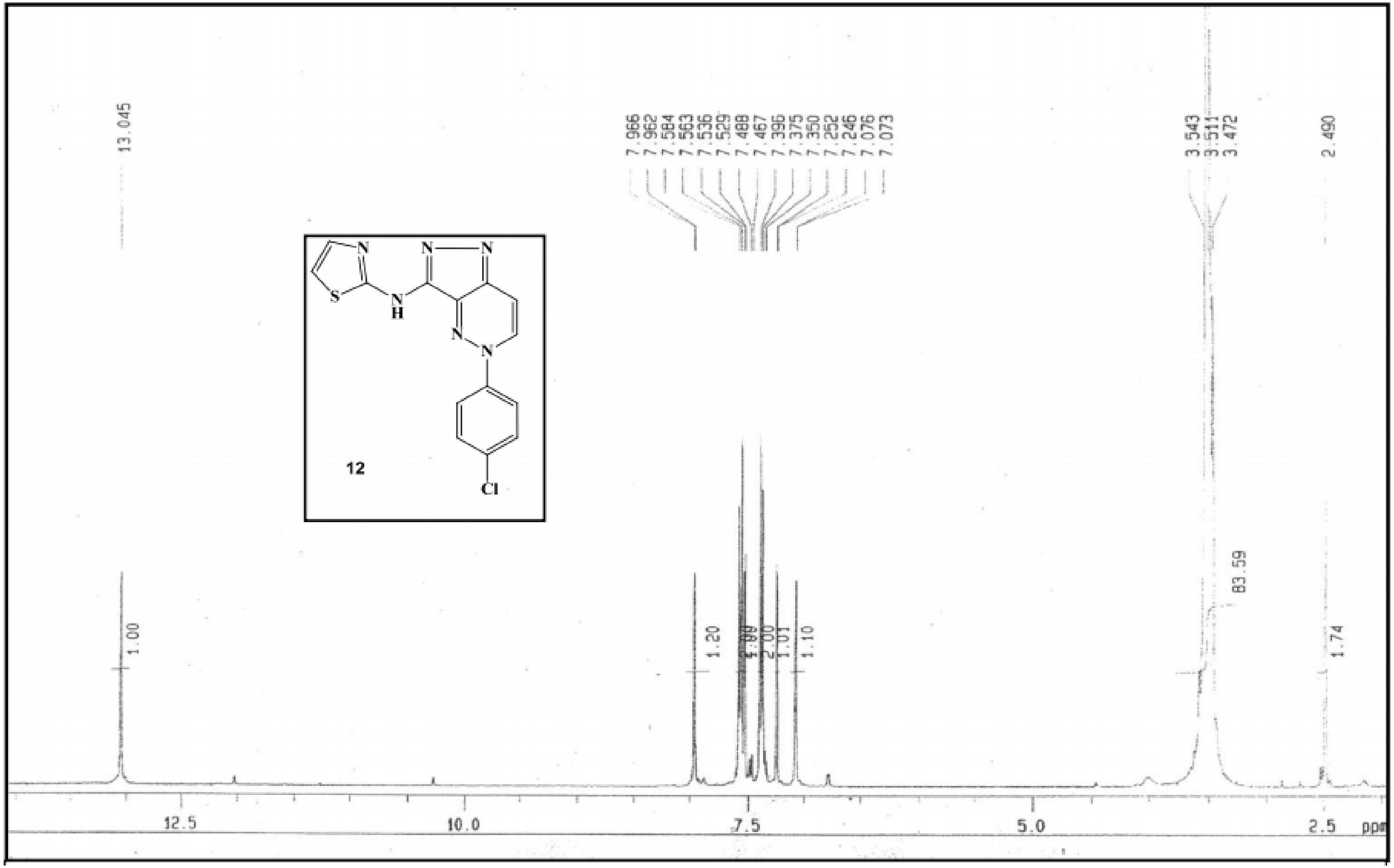

Figure S48. ^{1}H NMR spectrum of compound **12** (DMSO-d_6).

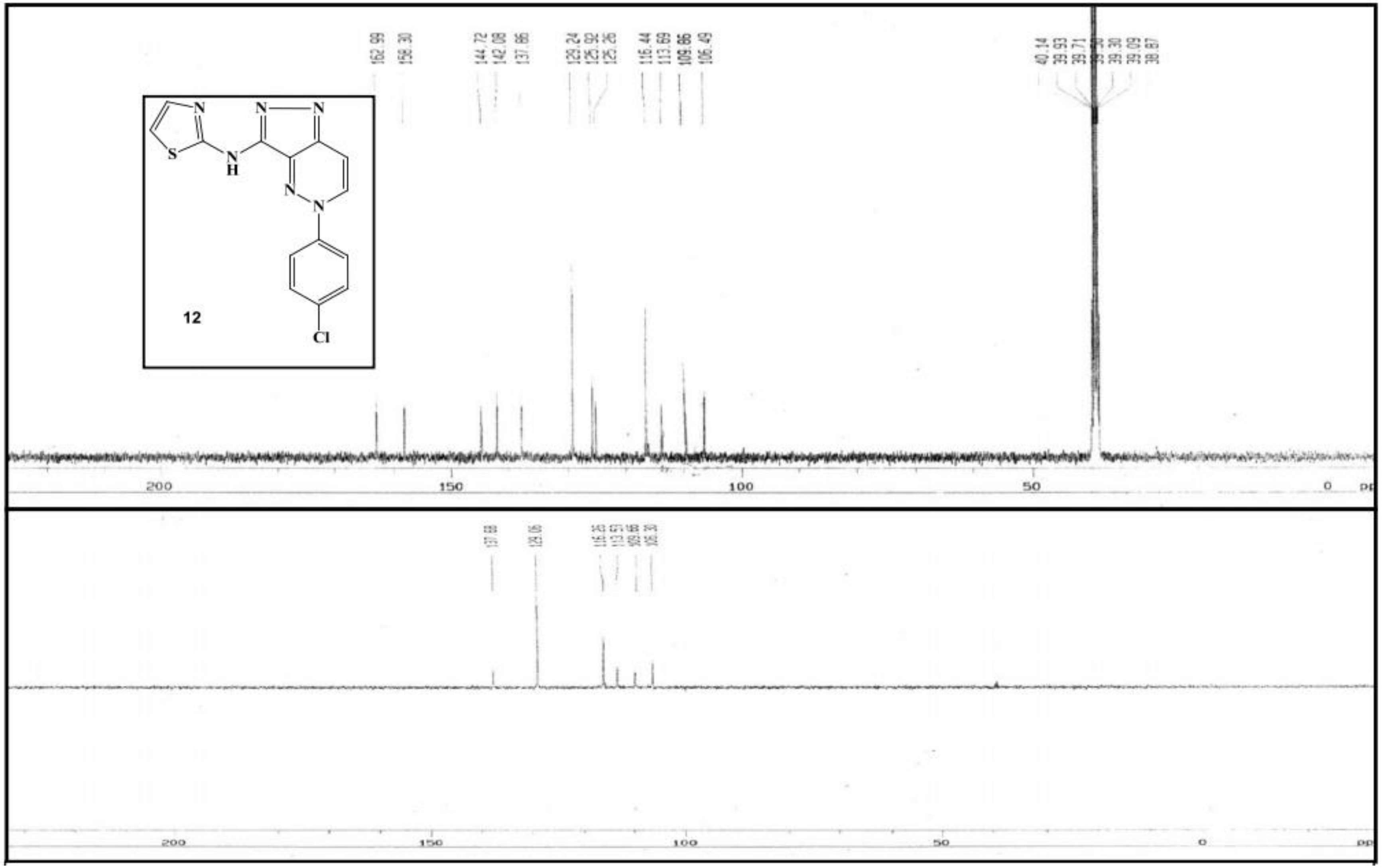

Figure S49. ^{13}C, DEPT-135 NMR spectrum of compound **12** (DMSO-d_6).

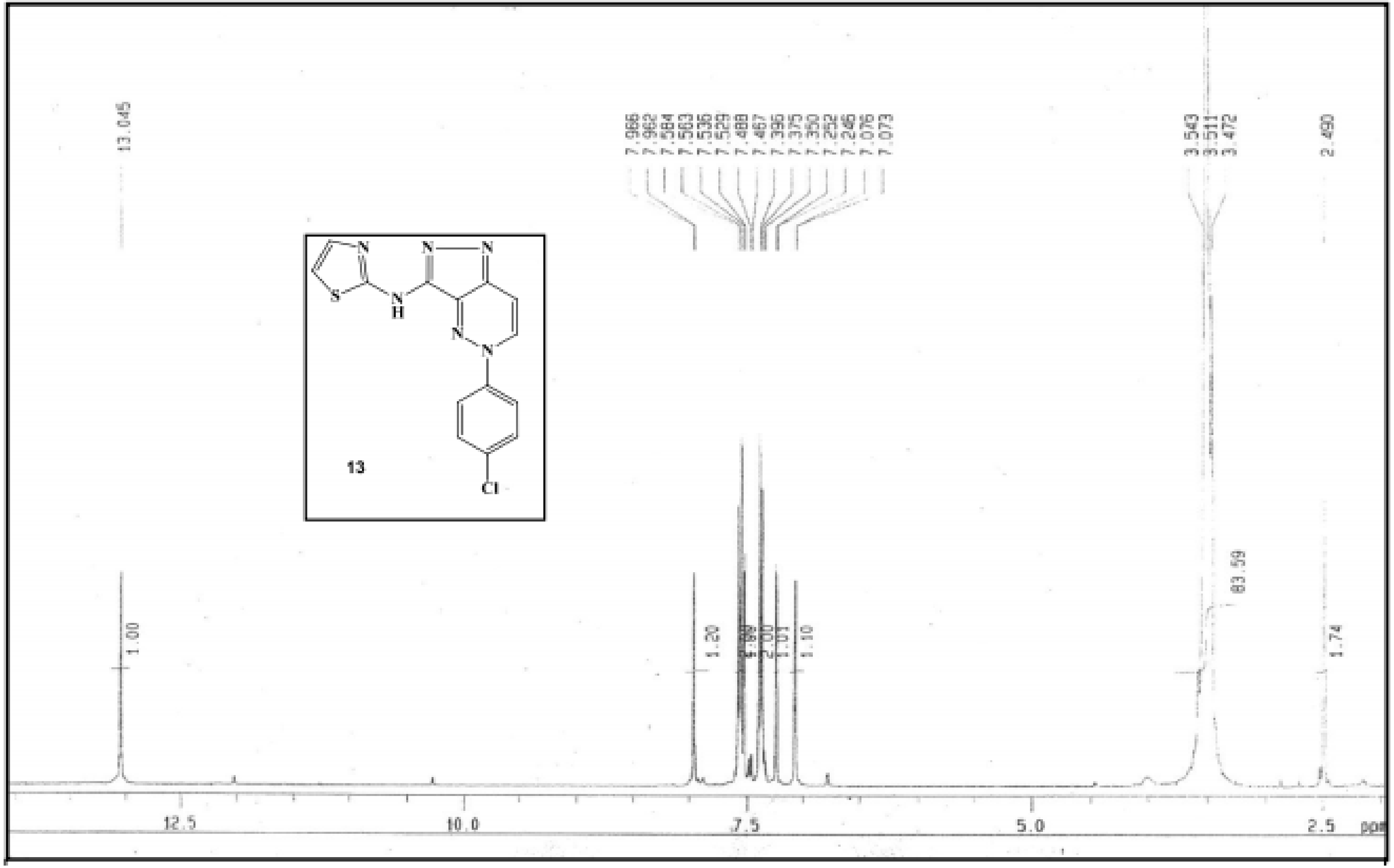

Figure S50. ^{1}H NMR spectrum of compound **13** (DMSO-d_6).

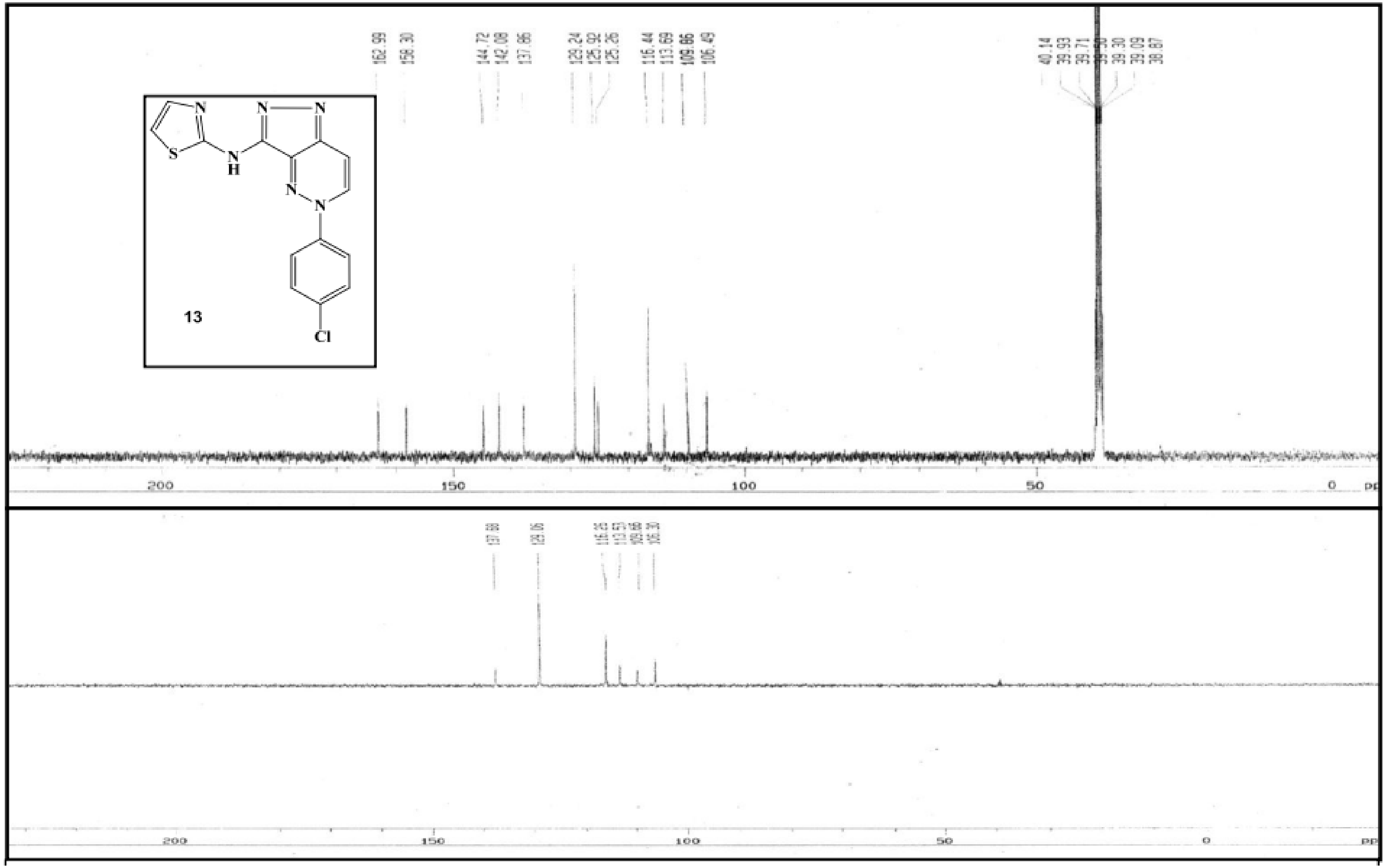

Figure S51. ^{13}C, DEPT-135 NMR spectrum of compound **13** (DMSO-d_6).
